# Intermolecular Synthesis of Coumarins from Acid Chlorides and Unactivated Alkynes through Palladium Catalysis

**DOI:** 10.1021/acs.orglett.5c02391

**Published:** 2025-08-05

**Authors:** Hendrik L. Schmitt, Niels Staeck, Patrick Müller, Michael K. Bogdos, Bill Morandi

**Affiliations:** Laboratorium für Organische Chemie, 27219 ETH Zurich, 8093 Zurich, Switzerland

## Abstract

We describe a modular synthetic pathway to directly obtain coumarins from acid chlorides and alkynes. A Pd catalyst employing 3,5-CF_3_-Ph-DPEPhos as the ancillary ligand was found to unlock this reactivity, enabling the conversion of a variety of 2-methoxy benzoyl chlorides and alkynes to the corresponding coumarins. Besides acid chlorides, the *in situ* generation of this reactive species from the corresponding acid was also possible. Finally, control experiments and preliminary kinetic analyses were performed to understand the role of the catalyst.

## Graphical abstract

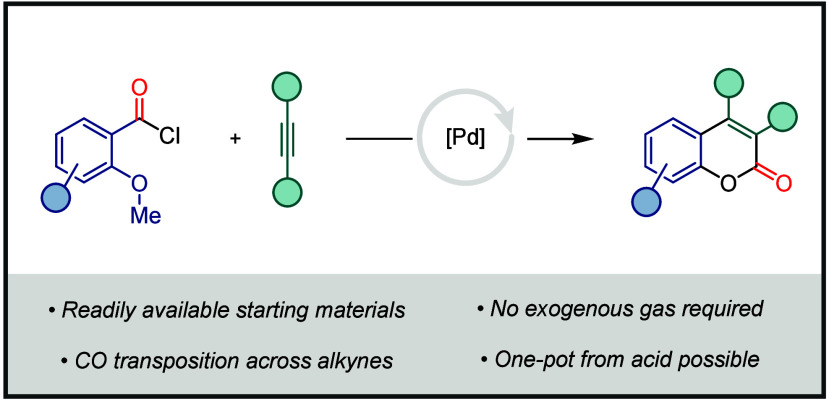

The development of new methods to build complex molecules from simple starting materials is at the core of synthetic organic chemistry. One synthetic motif of interest is the coumarin scaffold, which is commonly encountered in natural products, pharmaceuticals, or small-molecule fluorophores.^1–6^ Particularly 3- and 4-substitued coumarins are of interest due to their relevance in biological applications.^7–10^ To access coumarins, a variety of methods have been developed in the past ranging from traditional condensation approaches^6,7,11–15^ to intramolecular methods with^16–24^ and without^25–33^ transition metal catalysts (Scheme 1, A). Building on these annulation techniques, various intermolecular methods from simple starting materials have also been reported.^16,34–36^ One particularly compelling approach in this context involves the employment of alkynes in a carbofunctionalization reaction to construct a wide variety of 3- and 4-substituted coumarins by leveraging these readily available unsaturated building blocks. Among these reactions, those that do not require specific functional groups on the alkyne (e.g., alkynoic acid derivatives^16,37^ ) are especially advantageous, as they allow access to a more diverse spectrum of coumarin derivatives. While synthetically desirable, examples that enable such modular syntheses of coumarins from simple alkyne starting materials often rely on the use of carbon monoxide,^38–41^ a highly poisonous and flammable gas which requires special handling (Scheme 1, B (a)).^42–44^ Similar procedures that leverage the more benign CO_2_ in a carbocarboxylation of alkynes to obtain coumarins require subsequent steps to obtain the cyclic product.^45^

**Scheme 1:**
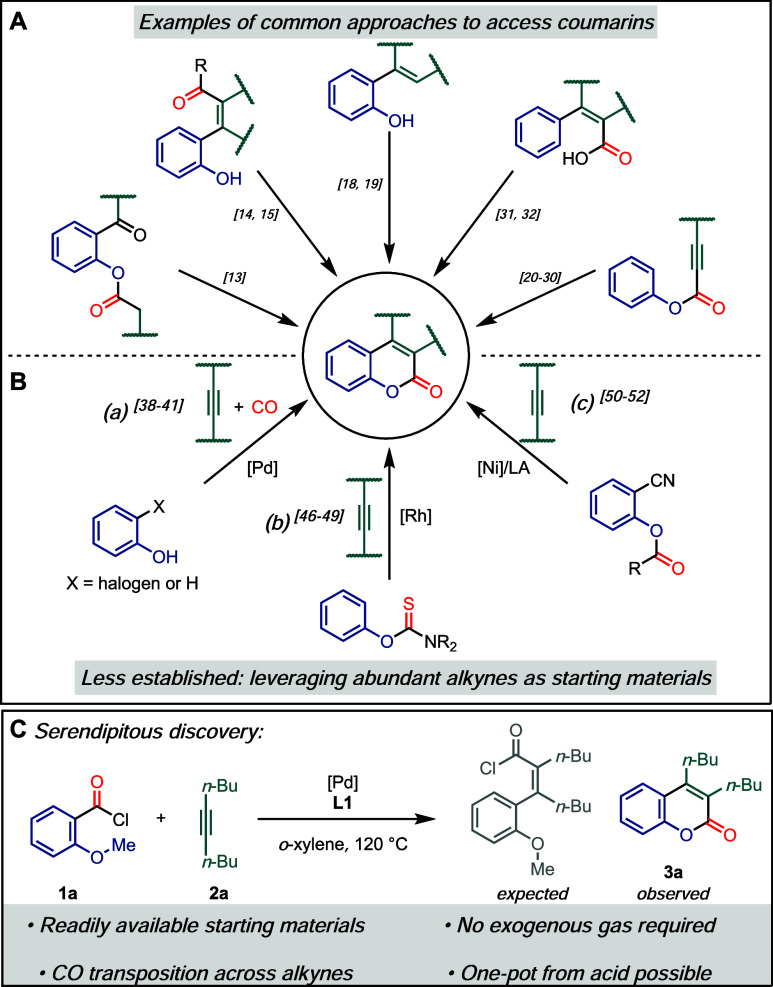
Methods to Access Coumarins and Discovery of Novel Reaction Conditions: **A**, Exemplary Traditional Approaches to Access Coumarins; **B**, Approaches to Access Coumarins Leveraging Intermolecular Reactions with Alkynes; **C**, Discovery of Novel Conditions to Access Coumarins from Anisoyl Chlorides and Alkynes

Beyond methods based on the addition of exogenous gases, only few reactions to obtain coumarins from alkynes in an intermolecular reaction have been reported. One synthetic sequence that achieves such reactivity relies on the Rh-catalyzed C–H activation of aryl thiocarbamates, followed by insertion into an alkyne and final formation of the product through desulfurization of the directing group (Scheme 1, B (b)).^46–49^ This route requires the presence of stoichiometric metal additives, exhibits restrictions with regard to the employed alkyne, and results in the unselective formation of coumarin isomers when substituents occupy positions other than the *para*-position. Another prominent example is the Ni-catalyzed construction from *o*-arylcarboxybenzonitriles, a seminal work in the functionalization of alkynes and activation of strong bonds (Scheme 1, B (c)).^50–52^ However, since Lewis acid additives are required for the activation of the nitrile, and an aryl ester is necessary, the overall atom economy is diminished.^53,54^

Our group has demonstrated the proficiency of acid chlorides to serve as readily available CO-surrogates.^55–58^ Throughout our recent endeavors to develop an intermolecular carbochlorocarbonylation reaction between acid chlorides and alkynes,^59^ we observed that when *ortho*-methoxy acid chloride **1a** was employed, coumarin derivative **3a** was obtained instead of the expected cinnamoyl chloride (Scheme 1, C).

We hypothesized that activation of the *ortho*-methoxy group to form the ester after transposition of the carbonyl across the alkyne occurred, representing a novel pathway to access the coumarin scaffold through intermolecular carbopalladation with an alkyne. This method omits the necessity to use exogenous gases while still allowing for the rapid synthesis of this motif of interest from readily accessible starting materials. Additionally, it does not rely on the addition of stoichiometric metal additives and can be applied to a variety of acid chloride derivatives.

To further improve on our initial findings and to determine the required reaction components for this novel reactivity, several control reactions were conducted (Table 1). Variations in the choice of ligand revealed that our previously employed 3,5-CF_3_-Ph-DPEPhos **L1** was best suited for producing the desired product in high yields. In comparison, the more electron-rich DPEPhos and Xantphos, which do not bear additional CF_3_ groups, were not competent. Comparable ligands such as the 3,5-CF_3_-Ph derivative of Xantphos **L2** also resulted in the formation of the desired product, albeit in reduced yields. Phosphine ligands beyond these bidentate examples did not result in any significant yield (Supporting Information). It was further determined that reaction efficiency could be improved when using [Pd­(allyl)­Cl]_2_ as a precatalyst and toluene as a solvent.

**Table 1:**
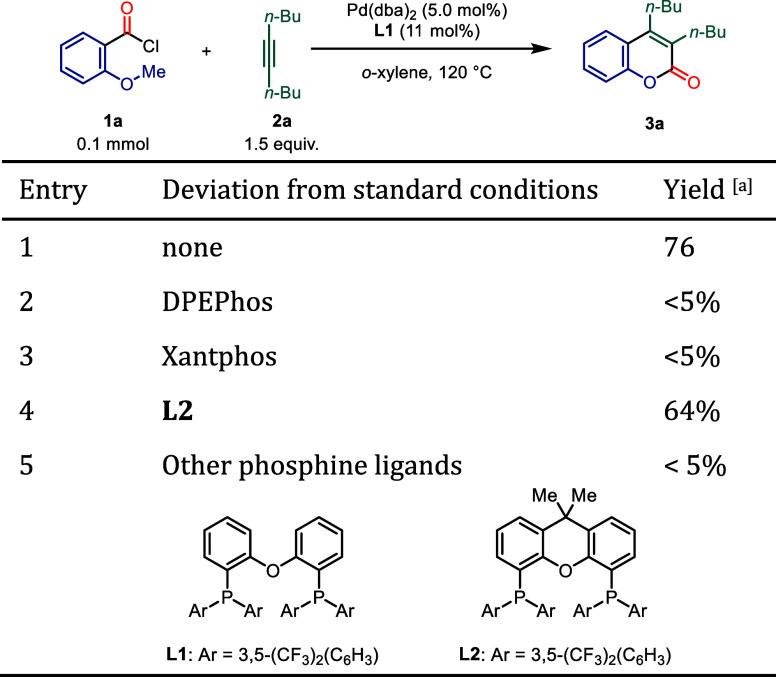
Optimization of Coumarin Formation

a Yields were either determined by GC-analysis with *n*-dodecane as internal standard or quant. ^1^H NMR analysis with 1,3,5-trimethoxy benzene as internal standard (see Supporting Information for more details).

To evaluate the scope of the reaction, a variety of 2-methoxy aroyl chloride derivatives and alkynes were tested. The reaction exhibited a tolerance toward a broad variety of functional groups including alkyl (**3b**, **3c**), halogen (**3d**, **3e**), or ether substituents (**3f**, **3g**, **3h**) and strongly electron-withdrawing moieties such as the trifluoromethyl (**3i**) and nitro group (**3j**). Furthermore, a sulfone (**3k**) and a pyrrole (**3l**) yielded the desired coumarins in 50% and 29% yield, respectively. To gain access to hydroxy-coumarins, we envisioned a two-step sequence, since the combination of an unprotected hydroxy group next to an acid chloride would not be possible on the substrate. Initially, boronic ester derivative **1m** would be employed to yield the corresponding coumarin, which, when oxidized with hydrogen peroxide, would furnish the product of interest. Through this two-step process hydroxycoumarin **3m** could be obtained in a 40% yield over two steps, highlighting the tolerance of our reaction toward boronic esters and providing a pathway to hydroxy coumarins. Next, a variety of alkynes were tested. Symmetrical alkynes **3n**, **3o**, and **3p** yielded the respective products, further exemplifying the tolerance for alkyl chlorides as in **3n**. When nonsymmetrical alkynes were subjected to the reaction conditions, mixtures of regioisomers were observed, which were readily separable on silica. Besides internal alkynes (**3q**), terminal alkynes were also tolerated (**3r**). When a TMS-acetylene derivative was employed instead, the reaction resulted in the sole formation of the 3-TMS-substituted product **3s**, which could then be protodesilylated to access 4-substituted products selectively (82%, for details see the Supporting Information). Ynamides also resulted in the selective formation of one isomer of coumarins in high yields (**3t**). The trends for the observed selectivity of the reaction for nonsymmetrical alkynes are in line with previous observations.^55^ (For an extended scope with limitations and discussion of reduced yields see the Supporting Information.)

Finally, we explored a potential one-pot method using *ortho*-methoxy benzoic acids as substrates (Scheme 2, bottom). From *ortho*-anisic acid, chlorodipyrrolidino­carbenium hexafluorophosphate (PyClU), and 1,8-bis­(*N,N*-dimethylamino)­naphthalene (proton sponge), the acid chloride was generated *in situ*, consistent with previous reports.^60^ The corresponding coumarin was obtained in comparable yield to using 2-methoxy benzoyl chloride **1a**.

**Scheme 2:**
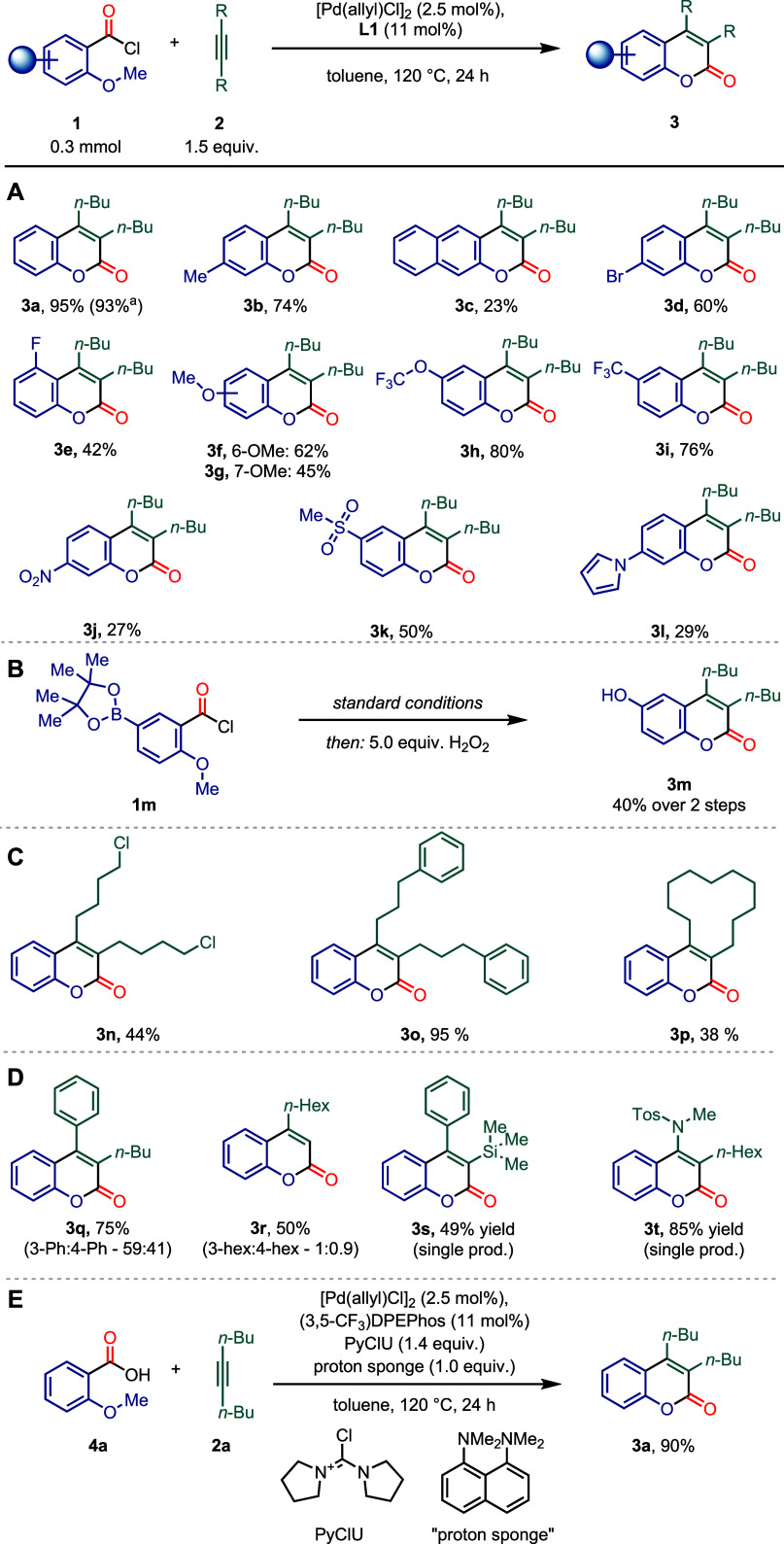
Scope. Yields Reported as Isolated Yields: **A,** Aryl Scope; **B**, Boryl Substrate with Subsequent Oxidation; **C,** Symmetrical Alkynes; **D,** Nonsymmetrical Alkynes; **E,** One-Pot Procedure from Acid

a 1 mmol scale.

To expand our understanding of the reaction, we conducted control reactions. Given the observed strong preference for the employed 3,5-CF_3_-Ph-DPEPhos ligand, we hypothesized that the coumarin synthesis proceeds via a mechanism that shares at least some elementary steps with our previously reported carbochlorocarbonylation, which likewise exhibited a strong dependence on this ligand.^59^

To elaborate on this, the reaction was followed over time to identify intermediate species in the reaction mixture upon MeOH quenching (Scheme 3, A). Besides the quenched starting material (**5a**) and product (**3a**), we also noted the formation of cinnamoyl ester (**6a**). The latter formed rapidly from the onset of the reaction but decreased once the coumarin production commenced, after a short induction period. This confirms that the carbochlorocarbonylation product (**6a**) is an accessible intermediate in the reaction. To determine whether this intermediate is crucial to access the coumarin or whether an independent mechanism from the starting materials to the product operates, we tried to fit simplified differential equation models (see Supporting Information for details). The best fit was obtained when the carbochlorocarbonylation product was assumed to serve as an intermediate from which annulation to the coumarin occurs directly.

**Scheme 3:**
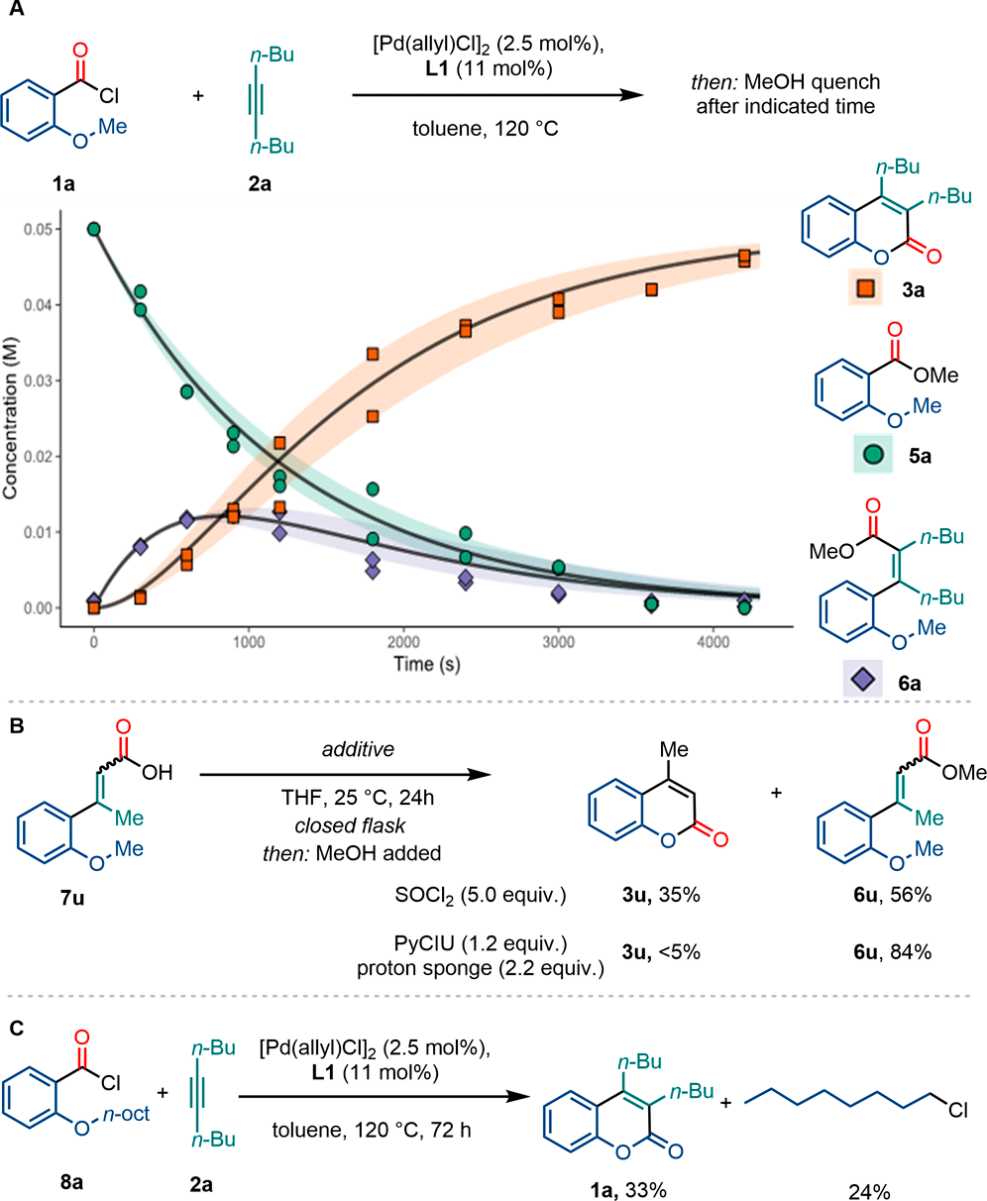
Reaction Profile with Kinetic Modeling and Control Reactions: **A,** Reaction Profile; the Data Set Is Constructed by Combining the Data Obtained through Two Independent Experiments; the Black Curve Represents the Predictions of the Model; the Ribbons Represent the Bounds of the Predictions Derived from the 99.7% Confidence Interval (See Supporting Information for Details); **B,** Acid Enabled Cyclization to Coumarins from Cinnamoyl Chlorides; **C,** Alkyl Fate Experiments^a^

a Yields for all experiments were determined by quant. NMR analysis with 1,3,5-trimethoxy benzene as internal standard (see Supporting Information for details).

Based on the observed reaction profile, we were interested in whether the putative cyclization from the transiently formed cinnamoyl chloride to the coumarin is catalyst dependent. We, therefore, synthesized the acid chloride analogue of the cinnamic acid **7u** as a mechanistic probe. When **7u** was converted *in situ* to the acid chloride, besides the expected cinnamoyl chloride, coumarin **3u** was obtained (Scheme 3, B). When the acid chloride formation was attempted with PyCIU, in the presence of “proton sponge” as a base, no coumarin and only **6u** could be observed. This indicates that annulation from *ortho-*methoxy cinnamoyl chlorides is accessible in the presence of an acid additive, e.g., HCl, which is the byproduct from *in situ* generation of acid chloride formation with SOCl_2_ in the experiment above. We thus surmise that HCl, which might form transiently from γ-elimination of cinnamoyl chlorides under our reaction conditions, could be involved in the final cyclization step.^59^ Alternatively, the ester formation from acid chlorides and ethers might originate from other Lewis acidic species in the reaction mixture, as precedented in the literature.^61–64^

Finally, to determine the fate of the alkyl residue on the ether, *n*-octyl derivative **8a** was synthesized. Upon subjecting this substrate to our reaction conditions, the formation of octyl chloride as a byproduct was observed, highlighting the importance of the chloride anion in yielding the desired ester functionality (Scheme 3, C).

In conclusion, we report a novel method to access coumarins through an intermolecular process from readily available *ortho*-methoxy benzoyl chlorides and alkynes. We demonstrate that the required acid chloride functionality can be generated *in situ* and allows for the direct synthesis of a broad variety of coumarins. Mechanistic experiments reveal that the observed ligand dependency might originate from the necessity to form cinnamoyl chloride intermediates and results in the formation of an alkyl chloride byproduct. The final annulation to the product might occur through an (Lewis-) acid-mediated pathway.

## Supplementary Material



## Data Availability

The data underlying this study are available in the published article and in its Supporting Information and are openly available via Zenodo at 10.5281/zenodo.15631805.

## Notes

General reaction procedures, characterization data of products, ^1^H, ^13^C, and ^19^F NMR spectra, and HRMS data of products, as well as additional optimization and mechanistic data (PDF)
