# Supplementary material for: Intermolecular Synthesis of Coumarins from Acid Chlorides and Unactivated Alkynes through Palladium Catalysis
Source: Org Lett. 2025 Aug 5;27(32):8869–74. doi: 10.1021/acs.orglett.5c02391 (PMC12362583; doi:10.1021/acs.orglett.5c02391)
Supplement: Supplementary file 1 [file ol5c02391_si_001.pdf]

Intermolecular Synthesis of Coumarins from Acid Chlorides and Unactivated Alkynes through Palladium Catalysis

Hendrik L. Schmitt[a], Niels Staeck[a], Patrick Müller[a], Michael K. Bogdos[a], Bill Morandi[a]

H. L. Schmitt, N. Staeck, P. Müller, M. K. Bogdos, B. Morandi.
Laboratorium für Organische Chemie, ETH Zürich
Vladimir-Prelog-Weg 3, HCI, 8093 Zürich, Switzerland

E-mail: bill.morandi@org.chem.ethz.ch

Table of Contents

1. General Information 3
2. Optimization of reaction parameters 4

Ligand Screening 4

Metal Source 6

Solvent and temperature 6

Ligand/Catalyst Loading/Molarity 7

3. Scope 8

Extended Scope 8

General procedure 1 9

In situ acid chloride generation procedure 23

Protodesilylation 23

4. Starting material synthesis 25

General procedure 2 25

Other compounds 25

5. Mechanistic investigations 27

Reaction profile 27

Ring closure from cinnamoyl chlorides 30

Coumarin formation in presence of base 32

Alkyl fate 33

6. References 34
7. NMR spectra 36

General Information

Unless otherwise noted, all reactions were carried out under argon in oven-dried 4 mL screw-cap glass vials using anhydrous solvents. The anhydrous solvents were prepared by distillation over appropriate drying agents or by using a solvent purification system (LC Technology Solutions, Inc.) under N_2 atmosphere (H_2O content: below 10 ppm, as determined by Karl Fischer titration) and stored over molecular sieves prior to use. All commercially available compounds were used as received from common suppliers (Sigma-Aldrich, Strem Chemicals, abcr, TCI, Fluorochem, Acros Organics, Alfa Aesar and Apollo Scientific).

Thin layer chromatography (TLC): Aluminum TLC plate, silica gel coated with fluorescent indicator F254 (TLC Silica gel 60 F254, Merck). Visualization was accomplished using UV light (254 nm) or $KMnO_4$ stain.

Flash column chromatography: SiliaFlash P60 silica gel (60 Å, 40−63 μm, SiliCycle Inc.) with reagent grade solvents.

NMR: Spectra were recorded on Bruker AVANCE III 400, Neo 400, Neo 500, or 600 spectrometers at room temperature, unless indicated otherwise; the chemical shifts are reported with respect to internal solvent: δH = 7.26 ppm, and δC = 77.16 (t) ppm ($CDCl_3$); δH = 7.16 ppm, and δC = 128.06 (t) ppm (C_6D_6); δH = 2.50 (p) ppm, and δC = 39.52 (hept) ppm (DMSO-d_6). Multiplicities are indicated by s (singlet), d (doublet), t (triplet), q (quartet), p (quintet), h (sextet), hept (septet), m (multiplet), br (broad), or combinations thereof.

GC/FID and GC/MS: Shimadzu GC-2025 (capillary column: Macherey-Nagel OPTIMA 5, 30.0 m × 0.25 mm × 0.25 μm; carrier gas: H_2); To determine GC yields, calibration curves were generated using *n*-dodecane as an internal standard. Shimadzu GCMS-QP2020 (capillary column: Macherey-Nagel OPTIMA 5, 30.0 m × 0.25 mm × 0.25 μm; carrier gas: He).

High-resolution MS (HRMS): Thermo scientific Q-Exactive GC Orbitrap for EI. Bruker Daltonics maXis ESI-QTOF or solariX ESI-FTICR-MS for ESI. HRMS data were obtained by the mass spectrometry service (MoBiAS, Molecular and Biomolecular Analysis Service) in the Laboratorium für Organische Chemie at ETH Zürich.

Optimization of reaction parameters

Ligand Screening

Table S1: Ligand optimization.

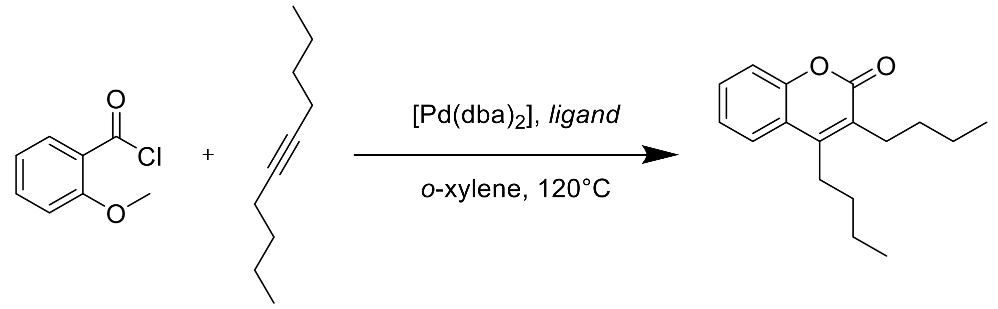

Entry	Ligand	Yield [%][a]
**1**	Xantphos	4
**2**	*t*BuXantphos	2
**3**	DPEPhos	0
**4**	DBFPhos	1
**5**	rac-BINAP	1
**6**	Dppf	0
**7**	BISBI	0
**8**	RuPhos	3
**9**	$(3,5\text{-}CF_3Ph)_3P$	2
**10**	PCy_3	0
**11**	PPh_3	0
**12**	3,5-Me-Xantphos	1
**13**	3,5-CF_3-Xantphos	64
**14**	3,5-CF_3-DPEphos	76

[a] The GC yields are based on the moles of the limiting reagent (0.10 mmol, 1.00 equiv.) versus n-dodecane. Typical screening conditions: *o*-anisoyl chloride (0.10 mmol, 1.00 equiv.), 5-decyne (0.15 mmol, 1.50 equiv.), metal source (5.00 µmol, 5.00 mol%), ligand (11.0 µmol, 11.0 mol%), *o*-xylene (0.17 mL, 0.60 M), 120 °C, 24h

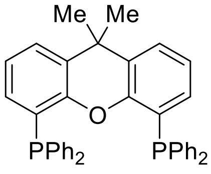

Xantphos

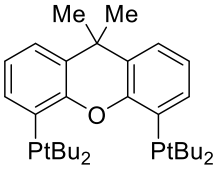

tBu-Xantphos

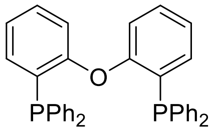

DPEPhos

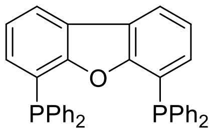

DBFPhos

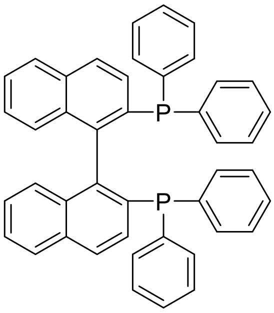

BINAP

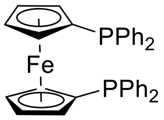

Dppf

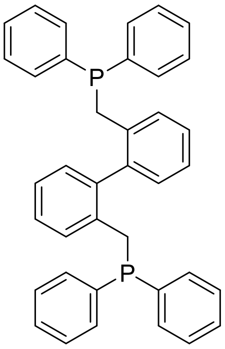

BISBI

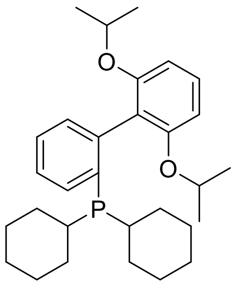

RuPhos

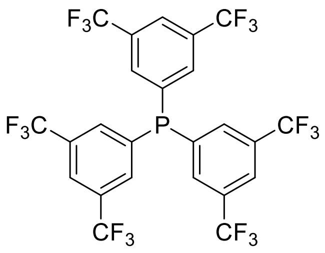

$(3,5\text{-}CF_3Ph)_3P$

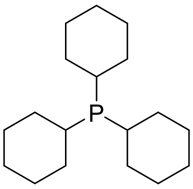

PCy_3

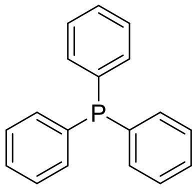

PPh_3

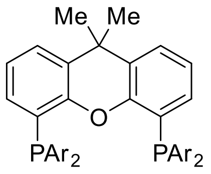

3,5-Me-Xantphos: Ar = 3,5-MePh
3,5-CF3-Xantphos: Ar = 3,5-CF_3Ph

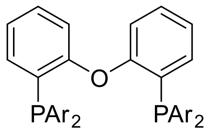

3,5-CF3-DPEphos
Ar = 3,5-CF_3Ph

Ligands were either obtained commercially, or (in case of the Xantphos and DPEPhos derivatives) synthesized according to our previous report.[1]

Metal Source

Table S2: Precatalyst optimization.

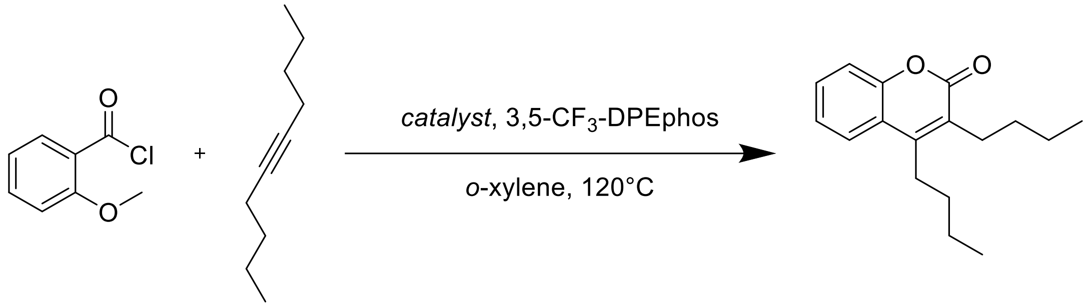

Entry	catalyst	Yield [%][a]
**1**	$Pd(dba)_2$ (5.0 mol%)	76
**2**	$Pd_2(dba)_3$ (2.5 mol%)	57
**3**	$[Pd(allyl)Cl]_2$ (2.5 mol%)	68
**4**	$Pd(OAc)_2$ (5.0 mol%)	0
**5**	$Ni(COD)_2$ (5.0 mol%)	1
**6**	$[Rh(COD)Cl]_2$ (2.5 mol%)	0

[a] The GC yields are based on the moles of the limiting reagent (0.10 mmol, 1.00 equiv.) versus n-dodecane. Typical screening conditions: *o*-anisoyl chloride (0.10 mmol, 1.00 equiv.), 5-decyne (0.15 mmol, 1.50 equiv.), metal source (5.00 µmol, 5.00 mol%), ligand (11.0 µmol, 11.0 mol%), *o*-xylene (0.17 mL, 0.60 M), 120 °C, 24h

Note: even though $[Pd(allyl)Cl]_2$ did not outperform $Pd(dba)_2$ it was used in the subsequent optimization, as it allowed for a simplified reaction set up (due to preferential solubility of the precatalyst). Quantitative yields could still be achieved upon changing the solvent.

Solvent and temperature

Table S3: Solvent and temperature optimization.

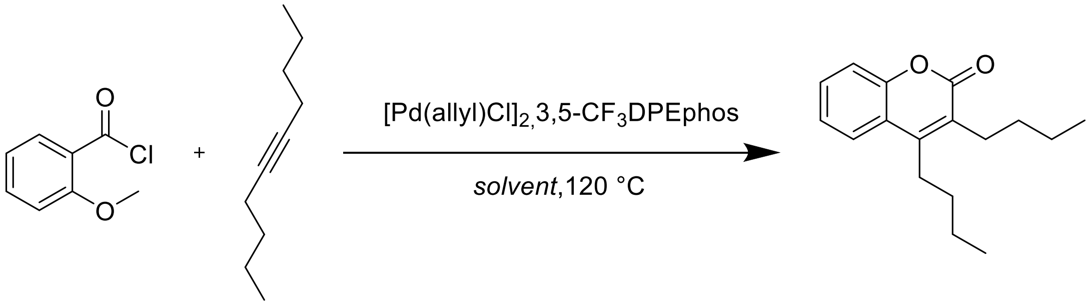

Entry	Solvent	Yield [%][a]
**1**	toluene	quant.
**2**	1,4-dioxane	78
**3**	chlorobenzene	33
**4**	toluene, 100 °C	92

[a] The GC yields are based on the moles of the limiting reagent (0.10 mmol, 1.00 equiv.) versus n-dodecane. Typical screening conditions: *o*-anisoyl chloride (0.10 mmol, 1.00 equiv.), 5-decyne (0.15 mmol, 1.50 equiv.), metal source (5.00 µmol, 5.00 mol%), ligand (11.0 µmol, 11.0 mol%), *o*-xylene (0.17 mL, 0.60 M), 120 °C, 24h

Ligand/Catalyst Loading/Molarity

Table S4: Concentration optimization.

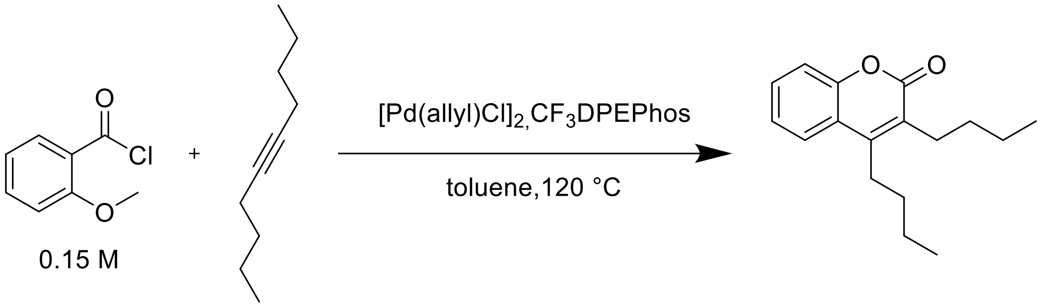

Entry	Loading	Yield [%][a]
**1**	5 mol% Pd and 11 mol% L	quant.
**2**	2.5 mol% Pd and 6 mol% L	5
**3**	5 mol% Pd and 6 mol% L	1.5
**4**	5 mol% Pd and 20 mol% L	quant.
**5**	1.1 equiv. alkyne	1.5
**6**	0.3 M	70

[a] The GC yields are based on the moles of the limiting reagent (0.10 mmol, 1.00 equiv.) versus n-dodecane. Typical screening conditions: *o*-anisoyl chloride (0.10 mmol, 1.00 equiv.), 5-decyne (0.15 mmol, 1.50 equiv.), metal source (5.00 µmol, 5.00 mol%), ligand (11.0 µmol, 11.0 mol%), *o*-xylene (0.17 mL, 0.60 M), 120 °C, 24h

Scope

Extended Scope

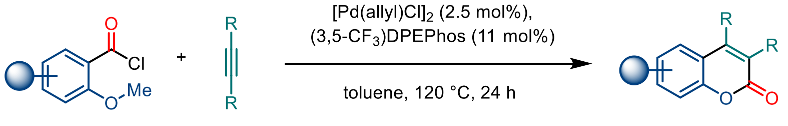

1 0.3 mmol | **2** 1.5 equiv. | **3**

successful examples:

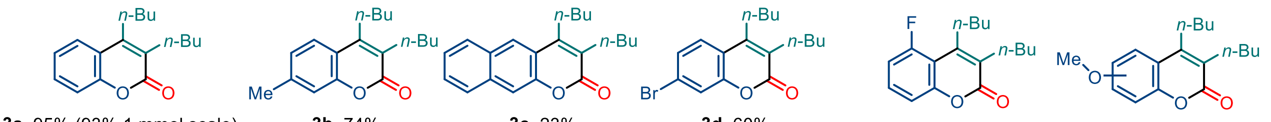

3a, 95% (93% 1 mmol scale) | **3b**, 74% | **3c**, 23% | **3d**, 60% | **3e**, 42% | **3f**, 6-OMe:62% **3g**, 7-OMe: 45%

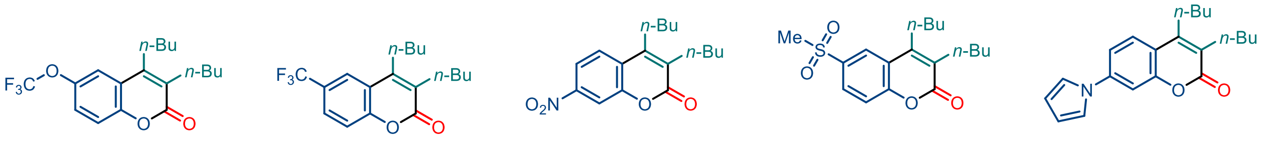

3h, 80% | **3i,** 76% | **3j,** 27% | **3k,** 50% | **3l,** 29%

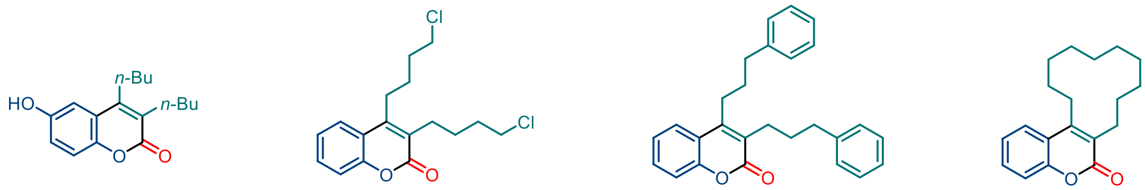

3m 40% over 2 steps | **3n,** 44% | **3o,** 95 % | **3p,** 38 %

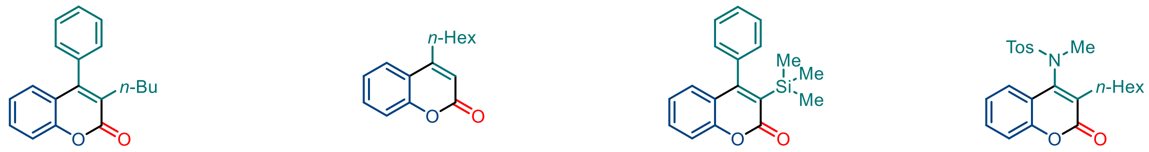

3q, 75% (3-Ph:4-Ph - 59:41) | **3r**, 50% (3-hex:4-hex - 1:0.9) | **3s,** 49% yield (single prod.) | **3t,** 85% yield (single prod.)

limitations / unsuccessful (or low yielding) substrate classes:

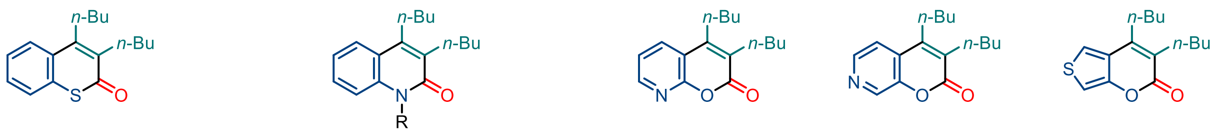

thioether substrates to obtain thiocoumarins | *amino substrates to obtain quinolinones* | *tested heterocylic precursors*

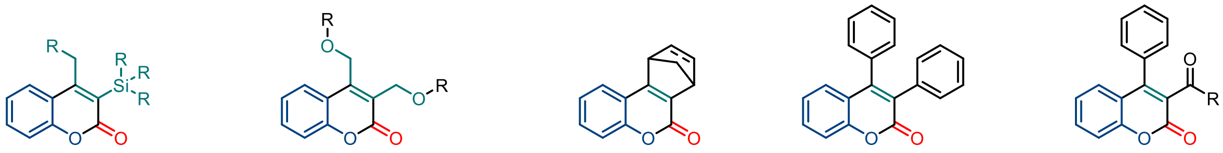

alkyl silyl alkynes | *alkynes with heteroatoms in propargylic positions* | *norbornene/ norbornadiene* | *diphenyl acetylenes* | *conjugated alkynes*

General procedure 1

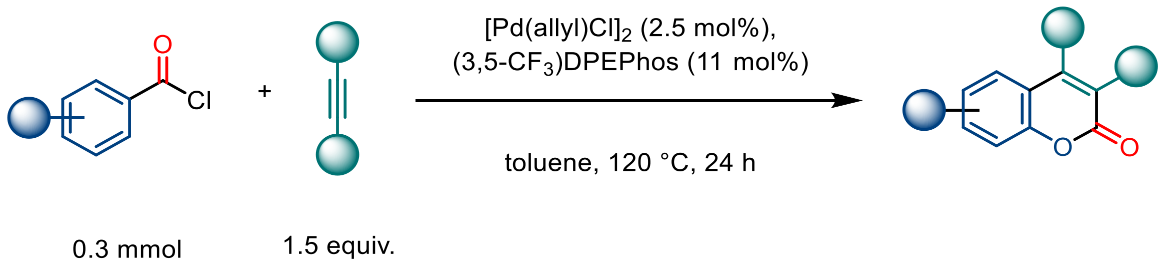

In a glovebox, to an oven dried 4 mL vial with a stir bar was added [Pd(allyl)Cl]$_2$ (2.74 mg, 7.50 µmol, 2.50 mol%,), followed by 3,5-CF_3DPEPhos (35.7 mg, 33.0 µmol, 11.0 mol%), and toluene (0.5 mL, 0.6 M). The mixture was stirred for 10 min before addition of alkyne (450 µmol, 1.50 equiv.) and acid chloride (300 µmol, 1.00 equiv.). The vial was then capped and removed from the glovebox and placed in a preheated stir plate at 120 °C, 1000 rpm for 24h/48h. Upon completion the solution was quenched with MeOH, filtered through a plug of silica, rinsed with EtOAc and volatiles were removed *in vacuo*. Subsequently purification was achieved by means of column chromatography or preparative TLC.

3,4-dibutyl-2H-chromen-2-one (3a)

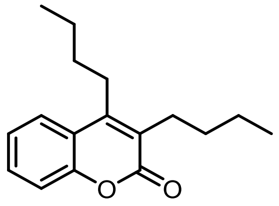

The title compound was synthesized according to general procedure 1 from 2-methoxy benzoyl chloride and 5-decyne. Purification by preparative TLC (SiO_2, 5% EtOAc in hexane) afforded **3a** as a yellow solid (77.5 mg, 286 µmol, 95%).

When the scale of general procedure 1 was increased to 1.00 mmol, the product could still be obtained in comparable yields (240 mg, 929 µmol, 93%).

^{1}H NMR (500 MHz, $CDCl_3$) δ 7.60 (d, J = 8.0, 1.4 Hz, 1H), 7.46 (m, 1H), 7.34 – 7.27 (m, 2H), 2.87 – 2.75 (m, 2H), 2.64 (m, 2H), 1.66 – 1.40 (m, 8H), 1.02 (t, J = 7.2 Hz, 3H), 0.98 (t, J = 7.2 Hz, 3H).

^{13}C{^{1}H} (125 MHz, $CDCl_3$) δ 161.9, 152.7, 150.1, 130.3, 126.6, 124.5, 124.1, 119.9, 117.0, 31.7, 31.3, 28.3, 27.5, 23.3, 23.1, 14.0, 14.0.

HRMS (ESI, m/z): $[M+Na]^+$ calcd. for $C_{17}H_{22}NaO_2$, 281.1512; found 281.1508.

The obtained analytical data matches with previous reports.[2]

3,4-dibutyl-7-methyl-2H-chromen-2-one (3b)

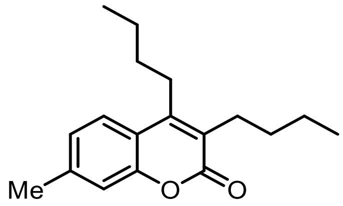

The title compound was synthesized according to general procedure 1 from 2-methoxy-4-methylbenzoyl chloride and 5-decyne. Purification by preparative TLC (SiO_2, 5% EtOAc in hexane) afforded **3b** as a white-yellow solid (60.4 mg, 222 μmol, 74%).

^{1}H NMR (400 MHz, $CDCl_3$) δ 7.44 (d, J = 8.1 Hz, 1H), 7.11 – 7.08 (m, 1H), 7.06 (m, 1H), 2.81 – 2.70 (m, 2H), 2.64 – 2.54 (m, 2H), 2.41 (s, 3H), δ 1.62 – 1.37 (m, 8H).), 0.98 (t, J = 7.2 Hz, 3H), 0.94 (t, J = 7.3 Hz, 3H).

^{13}C{^{1}H} (101 MHz, $CDCl_3$) δ 162.2, 152.7, 150.2, 141.3, 125.4, 125.2, 124.3, 117.5, 117.2, 31.7, 31.3, 28.4, 27.5, 23.3, 23.1, 21.5, 14.0, 14.0.

HRMS (ESI, m/z): $[M+H]^+$ calcd. For $C_{18}H_{25}O_2$, 273.1849; found 273.1848.

3,4-dibutyl-2H-benzo[g]chromen-2-one (3c)

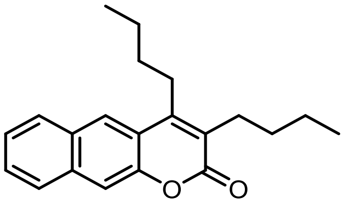

The title compound was synthesized according to general procedure 1, however running the reaction for 48h instead of 24h, from 3-methoxy-2-naphthoyl chloride and 5-decyne. Purification by preparative TLC (SiO_2, 12% EtOAc in hexane) afforded **3c** as a white solid (21.4 mg, 69.0 μmol, 23%).

^{1}H NMR (400 MHz, $CDCl_3$) δ 8.05 (s, 1H), 7.92 (d, J = 8.2 Hz, 1H), 7.84 (d, J = 8.2 Hz, 1H), 7.68 (s, 1H), 7.61 – 7.51 (m, 1H), 7.47 (ddd, J = 8.1, 6.8, 1.3 Hz, 1H), 3.01 – 2.80 (m, 2H), 2.80 – 2.54 (m, 2H), 1.76 – 1.64 (m, 2H), 1.59 (q, J = 7.3 Hz, 4H), 1.50 – 1.40 (m, 2H), 1.04 (t, J = 7.2 Hz, 3H), 0.98 (t, J = 7.2 Hz, 3H).

$^{13}C\{^1H\}$ (101 MHz, $CDCl_3$) δ 161.9, 149.8, 149.6, 134.0, 130.2, 128.6, 127.8, 127.3, 127.2, 125.5, 124.6, 120.1, 112.7, 31.8, 31.4, 28.5, 27.8, 23.4, 23.2, 14.1, 14.0.

HRMS (ESI, m/z): $[M+H]^+$ calcd. For $C_{21}H_{25}O_2$, 309.1849; found 309.1850.

Note: A reduced yield was observed for this substrate due to the formation of a Friedel-Crafts acylation product with the naphtyl's 1-position.

7-bromo-3,4-dibutyl-2H-chromen-2-one (3d)

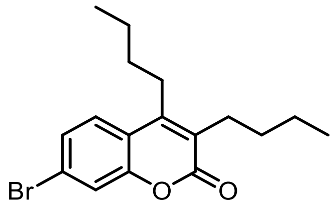

The title compound was synthesized according to general procedure 1 from 4-bromo-2-methoxybenzoyl chloride and 5-decyne. Purification by preparative TLC (SiO_2, 4% EtOAc in hexane) afforded **3d** as a white solid (61.0 mg, 181 µmol, 60%).

^1H NMR (400 MHz, $CDCl_3$) δ 7.47 (d, J = 1.9 Hz, 1H), 7.43 (d, J = 8.6 Hz, 1H), 7.38 (dd, J = 8.6, 1.9 Hz, 1H), 2.79 – 2.72 (m, 2H), 2.64 – 2.55 (m, 2H), 1.61 – 1.47 (m, 8H), 1.00 (t, J = 7.1 Hz, 3H), 0.96 (t, J = 7.2 Hz, 3H).

$^{13}C\{^1H\}$ (101 MHz, $CDCl_3$) δ 161.3, 153.0, 149.5, 127.5, 127.0, 125.7, 123.9, 120.2, 119.0, 31.7, 31.2, 28.4, 27.7, 23.3, 23.1, 14.1, 14.0.

HRMS (ESI, m/z): $[M+H]^+$ calcd. For $C_{17}H_{22}BrO_2$, 337.0798; found 337.0802.

3,4-dibutyl-5-fluoro-2H-chromen-2-one (3e)

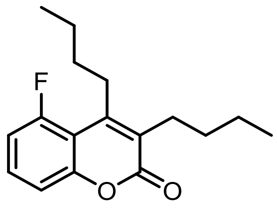

The title compound was synthesized according to general procedure 1, however running the reaction for 48h instead of 24h, from 2-fluoro-6-methoxybenzoyl chloride and 5-decyne.

Purification by preparative TLC (SiO_2, 10% Et_2O in hexane) afforded **3e** as a yellow solid (34.7 mg, 126 μmol, 42%).

^{1}H NMR (500 MHz, $CDCl_3$) δ 7.37 (m, 1H), 7.14 – 7.07 (m, 1H), 6.95 (m, 1H), 2.92 – 2.83 (m, 2H), 2.66 – 2.58 (m, 2H), 1.62 – 1.37 (m, 8H), 0.97 (m, 6H).

^{13}C{^{1}H} (125 MHz, $CDCl_3$) δ 161.0, 159.0 (d, *J* = 254.7 Hz), 153.5 (d, *J* = 6.3 Hz), 148.8 (d, *J* = 5.4 Hz), 130.2 (d, *J* = 11.3 Hz), 127.4 (d, *J* = 1.8 Hz), 113.2 (d, *J* = 3.6 Hz), 111.6 (d, *J* = 25.0 Hz), 110.1 (d, *J* = 12.8 Hz), 31.9 (d, *J* = 3.4 Hz), 31.2, 30.9 (d, *J* = 11.4 Hz), 26.9, 23.2, 23.0, 13.9, 13.8.

^{19}F{^{1}H} (376 MHz, $CDCl_3$) δ -112.6

HRMS (ESI, m/z): $[M+H]^+$ calcd. For $C_{17}H_{22}FO_2$, 277.1598; found 277.1601.

3,4-dibutyl-6-methoxy-2H-chromen-2-one (3f)

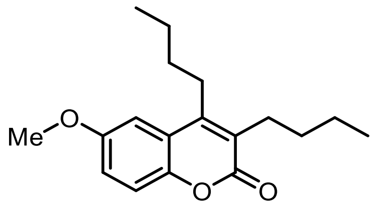

The title compound was synthesized according to general procedure 1 from 2,4-dimethoxybenzoyl chloride and 5-decyne. Purification by preparative TLC (SiO_2, 20% EtOAc in hexane) afforded **3f** as a yellow solid (53.9 mg, 187 μmol, 62%).

^{1}H NMR (400 MHz, $CDCl_3$) δ 7.26 – 7.22 (m, 1H), 7.07 – 6.99 (m, 2H), 3.85 (s, 3H), 2.98 – 2.67 (m, 2H), 2.68 – 2.46 (m, 2H), 1.65 – 1.39 (m, 8H), 1.01 (t, *J* = 7.1 Hz, 3H), 0.96 (t, *J* = 7.2 Hz, 3H).

^{13}C{^{1}H} (101 MHz, $CDCl_3$) δ 162.1, 155.9, 149.7, 147.1, 127.1, 120.5, 117.9, 116.9, 108.4, 56.0, 31.5, 31.3, 28.4, 27.7, 23.3, 23.1, 14.1, 14.0.

HRMS (ESI, m/z): $[M+H]^+$ calcd. For $C_{18}H_{25}O_3$, 289.1798; found 289.1799.

3,4-dibutyl-7-methoxy-2H-chromen-2-one (3g)

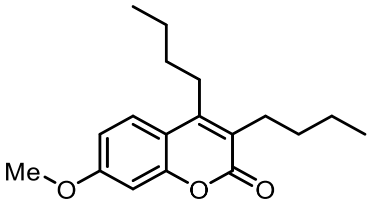

The title compound was synthesized according to general procedure 1 from 2,5-dimethoxybenzoyl chloride and 5-decyne. Purification by preparative TLC (SiO_2, 20% EtOAc in hexane) afforded **3g** as a yellow solid (39.1 mg, 136 μmol, 45%).

^{1}H NMR (400 MHz, $CDCl_3$) δ 7.47 (d, J = 8.8 Hz, 1H), 6.84 (dd, J = 8.8, 2.6 Hz, 1H), 6.80 (d, J = 2.5 Hz, 1H), 3.85 (s, 3H), 2.79 – 2.71 (m, 2H), 2.62 – 2.54 (m, 2H), 1.63 – 1.37 (m, 8H), 0.99 (t, J = 7.2 Hz, 3H), 0.95 (t, J = 7.3 Hz, 3H).

^{13}C{^{1}H} (101 MHz, $CDCl_3$) δ 162.4, 161.6, 154.3, 150.4, 125.6, 123.4, 113.5, 112.2, 100.8, 55.8, 31.8, 31.4, 28.5, 27.4, 23.3, 23.1, 14.1, 14.0.

HRMS (ESI, m/z): $[M+Na]^+$ calcd. For $C_{18}H_{24}NaO_3$, 311.1618; found 311.1618.

3,4-dibutyl-6-(trifluoromethoxy)-2H-chromen-2-one (3h)

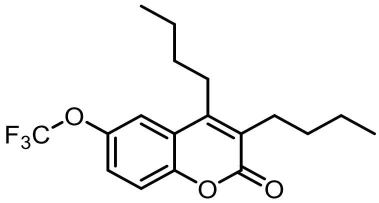

The title compound was synthesized according to general procedure 1 from 2-methoxy-5-(trifluoromethoxy)benzoyl chloride and 5-decyne. Purification by preparative TLC (SiO_2, 5% EtOAc in hexane) afforded **3h** as a yellow solid (82.1 mg, 240 μmol, 80%).

^{1}H NMR (500 MHz, $CDCl_3$) δ 7.40 (dq, J = 1.7, 0.9 Hz, 1H), 7.35 – 7.27 (m, 2H), 2.83 – 2.70 (m, 2H), 2.69 – 2.54 (m, 2H), 1.74 – 1.50 (m, 6H), 1.48 – 1.39 (m, 2H), 1.01 (t, J = 7.1 Hz, 3H), 0.96 (t, J = 7.2 Hz, 3H).

^{13}C{^{1}H} (125 MHz, $CDCl_3$) δ 161.3, 150.9, 149.0, 145.2 (q, J = 2.1 Hz), 128.1, 123.3, 120.9, 120.6 (q, J = 257.6 Hz), 118.4, 117.2, 31.5, 31.2, 28.4, 27.7, 23.2, 23.1, 14.0, 13.9.

^{19}F{^{1}H} (376 MHz, $CDCl_3$) δ -58.3.

HRMS (ESI, m/z): $[M+H]^+$ calcd. for $C_{18}H_{22}F_3O_3$, 343.1516; found 343.1507.

3,4-dibutyl-6-(trifluoromethyl)-2H-chromen-2-one (3i)

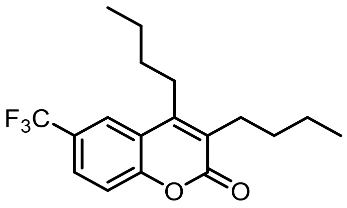

The title compound was synthesized according to general procedure 1, however running the reaction for 48h instead of 24h, from 2-methoxy-5-(trifluoromethyl)benzoyl chloride and 5-decyne. Purification by preparative TLC (SiO_2, 10% Et_2O in hexane) afforded **3i** as a yellow oil (74.4 mg, 228 μmol, 76%).

^{1}H NMR (500 MHz, $CDCl_3$) δ 7.82 (s, 1H), 7.68 (dd, J = 8.6, 2.1 Hz, 1H), 7.39 (d, J = 8.6 Hz, 1H), 2.87 – 2.75 (m, 2H), 2.68 – 2.58 (m, 2H), 1.65 – 1.37 (m, 8H), 1.01 (t, J = 7.2 Hz, 3H), 0.96 (t, J = 7.2 Hz, 3H).

^{13}C{^{1}H} (125 MHz, $CDCl_3$) δ 161.0, 154.6 (d, J = 1.3 Hz), 149.2, 128.2, 127.0 (q, J = 3.5 Hz), 127.1 – 126.1 (q, J = 33.0 Hz), 123.9 (q, J = 272.0 Hz), 122.1 (q, J = 4.0 Hz), 120.0, 117.8, 31.6, 31.2, 28.2, 27.7, 23.2, 23.1, 14.0, 13.9.

^{19}F{^{1}H} (376 MHz, $CDCl_3$) δ -62.0

HRMS (ESI, m/z): $[M+H]^+$ calcd. For $C_{18}H_{22}F_3O_2$, 327.1566; found 327.1570.

3,4-dibutyl-7-nitro-2H-chromen-2-one (3j)

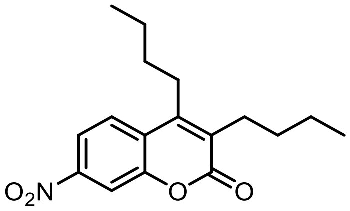

The title compound was synthesized according to general procedure 1, however running the reaction for 48h instead of 24h, from 2-methoxy-4-nitrobenzoyl chloride and 5-decyne. Purification by preparative TLC (SiO_2, 20% Et_2O in hexane) afforded **3j** as a white-yellow solid (83.1 mg, 66.0 μmol, 27%).

^{1}**H NMR** (400 MHz, $CDCl_3$) δ 8.11 (m, 2H), 7.76 – 7.69 (d, *J* = 8.4 Hz, 1H), 2.88 – 2.78 (m, 2H), 2.70 – 2.60 (m, 2H), 1.65 – 1.39 (m, 8H), 1.02 (t, *J* = 7.1 Hz, 3H), 0.97 (t, *J* = 7.2 Hz, 3H).

^{13}**C{^{1}H}** (101 MHz, $CDCl_3$) δ 160.6, 152.4, 148.5, 148.2, 130.5, 125.6, 125.1, 118.7, 112.7, 31.5, 31.1, 28.6, 28.0, 23.3, 23.1, 14.0, 14.0.

HRMS (ESI, m/z): $[M+H]^+$ calcd. For $C_{17}H_{22}NO_4$, 304.1543; found 304.1544.

Note: The observed reduced yield might originate from solubility issues of the starting material, or the strong electronic effect of the nitro group.

3,4-dibutyl-6-(methylsulfonyl)-2H-chromen-2-one (3k)

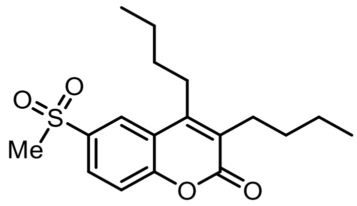

The title compound was synthesized according to general procedure 1 from 2-methoxy-5-(methylsulfonyl)benzoyl chloride and 5-decyne. Purification by preparative TLC (SiO_2, 30% EtOAc in hexane) afforded **3k** as a white solid (50.7 mg, 151 μmol, 50%).

^{1}**H NMR** (400 MHz, $CDCl_3$) δ 8.18 (d, *J* = 2.1 Hz, 1H), 7.98 (dd, *J* = 8.6, 2.1 Hz, 1H), 7.46 (d, *J* = 8.6 Hz, 1H), 3.09 (s, 3H), 2.90 – 2.80 (m, 2H), 2.76 – 2.49 (m, 2H), 1.62 – 1.51 (m, 6H), 1.48 – 1.39 (m, 2H), 1.01 (t, *J* = 7.1 Hz, 3H), 0.96 (t, *J* = 7.3 Hz, 3H).

^{13}**C{^{1}H}** (101 MHz, $CDCl_3$) δ 160.6, 155.8, 149.2, 136.4, 128.9, 128.8, 124.9, 120.5, 118.5, 44.9, 31.6, 31.1, 28.3, 27.8, 23.2, 23.1, 14.0, 13.9.

HRMS (ESI, m/z): $[M+H]^+$ calcd. for $C_{18}H_{25}O_4S$, 337.1468; found 337.1467.

3,4-dibutyl-7-(1H-pyrrol-1-yl)-2H-chromen-2-one (3l)

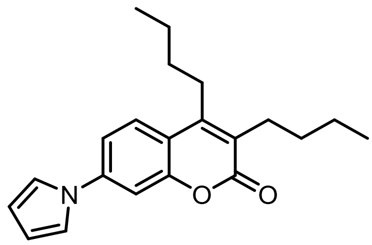

The title compound was synthesized according to general procedure 1 from 2-methoxy-4-(1H-pyrrol-1-yl)benzoyl chloride and 5-decyne. Purification by preparative TLC (SiO_2, 80% DCM in hexane) afforded **3l** as a white solid (28.6 mg, 88.4 μmol, 29%).

^{1}H NMR (400 MHz, $CDCl_3$) δ 7.65 – 7.58 (m, 1H), 7.39 – 7.30 (m, 2H), 7.21 – 7.10 (m, 2H), 6.48 – 6.34 (m, 2H), 2.85 – 2.73 (m, 2H), 2.67 – 2.56 (m, 2H), 1.63 – 1.51 (m, 6H), 1.50 – 1.40 (m, 2H), 1.02 (t, J = 7.2 Hz, 3H), 0.97 (t, J = 7.3 Hz, 3H).

^{13}C{^{1}H} (101 MHz, $CDCl_3$) δ 161.8, 153.6, 149.8, 142.0, 125.9, 125.7, 119.2, 117.3, 115.7, 111.7, 107.7, 31.7, 31.3, 28.5, 27.5, 23.3, 23.1, 14.1, 14.0.

HRMS (ESI, m/z): $[M+H]^+$ calcd. for $C_{21}H_{26}NO_2$, 324.1958; found 324.1961.

Note: The observed reduced yield might originate from instability of the pyrrole motif under our reaction conditions. The starting material already underwent a color change over time from a white solid to a blackish oil under prolonged room temperature storage conditions.

3,4-dibutyl-7-(1H-pyrrol-1-yl)-2H-chromen-2-one (3m)

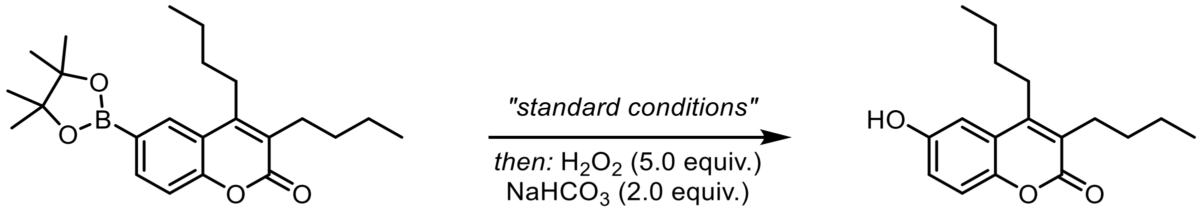

The title compound was synthesized according to general procedure 1 from 2-methoxy-5-(4,4,5,5-tetramethyl-1,3,2-dioxaborolan-2-yl)benzoyl chloride and 5-decyne. Upon completion of the reaction, volatiles were removed *in vacuo* and the obtained residue redissolved in a 1:1 THF:H_2O mixture (1 mL). Then, H_2O_2 (35% solution in water, 131 μL, 1.50 mmol, 5.00 equiv.) was added dropwise, followed by $NaHCO_3$ (50.4 mg, 0.60 mmol, 2.00 equiv.). The resulting mixture was stirred at room temperature for 1 hour, diluted with water (5 mL) and extracted with DCM (4 × 10 mL). The combined organic residues were dried over $MgSO_4$ and volatiles were removed *in vacuo*. Purification by preparative TLC (SiO_2, 25% EtOAc in hexane) afforded **3m** as a brown solid (32.6 mg, 119 μmol, 40%).

^{1}H NMR (400 MHz, $CDCl_3$) δ 7.18 (d, J = 8.8 Hz, 1H), 7.06 (d, J = 2.7 Hz, 1H), 7.00 (dd, J = 8.8, 2.8 Hz, 1H), 2.78 – 2.70 (m, 2H), 2.65 – 2.57 (m, 2H), 1.63 – 1.37 (m, 8H), 0.97 (m, 6H).

$^{13}C\{^1H\}$ (101 MHz, $CDCl_3$) δ 162.6, 152.3, 150.1, 146.9, 126.9, 120.7, 118.5, 118.0, 109.8, 31.5, 31.3, 28.6, 27.6, 23.4, 23.1, 14.1, 14.0.

HRMS (ESI, m/z): $[M+Na]^+$ calcd. For $C_{17}H_{22}NaO_3$, 297.1461; found 297.1459.

3,4-bis(4-chlorobutyl)-2H-chromen-2-one (3n)

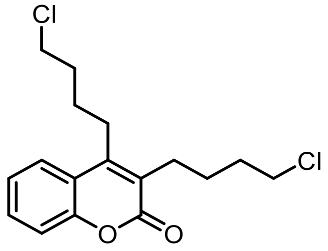

The title compound was synthesized according to general procedure 1 from 2-methoxybenzoyl chloride and 1,10-dichlorodec-5-yne. Purification by preparative TLC (SiO_2, 100% DCM) afforded **3n** as a yellow solid (42.9 mg, 131 µmol, 44%).

^{1}H NMR (400 MHz, $CDCl_3$) δ 7.60 (dd, J = 8.0, 1.4 Hz, 1H), 7.47 (ddd, J = 8.3, 7.2, 1.5 Hz, 1H), 7.33 – 7.31 (m, 1H), 7.29 (ddd, J = 8.0, 7.2, 1.2 Hz, 1H), 3.63 (t, J = 6.1 Hz, 2H), 3.61 (t, J = 6.1 Hz, 2H), 2.96 – 2.79 (m, 2H), 2.77 – 2.62 (m, 2H), 2.04 – 1.96 (m, 2H), 1.95 – 1.88 (m, 2H), 1.85 – 1.77 (m, 2H), 1.77 – 1.69 (m, 2H).

^{13}C{^{1}H} (101 MHz, $CDCl_3$) δ 161.8, 152.7, 149.8, 130.8, 126.1, 124.5, 124.4, 119.6, 117.2, 44.8, 44.4, 32.5, 32.5, 27.8, 26.9, 26.7, 26.3.

HRMS (ESI, m/z): $[M+H]^+$ calcd. for $C_{17}H_{21}Cl_2O_2$, 327.0913; found 327.0906.

3,4-bis(3-phenylpropyl)-2H-chromen-2-one (3o)

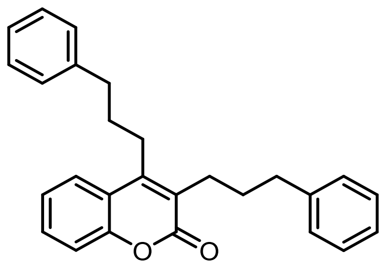

The title compound was synthesized according to general procedure 1 from 2-methoxybenzoyl chloride and 1,8-diphenyloct-4-yne. Purification by preparative TLC (SiO_2, 5% EtOAc in hexane) afforded **3o** as a yellow solid (109 mg, 285 µmol, 95%).

^{1}H NMR (400 MHz, $CDCl_3$) δ 7.43 (ddd, J = 8.2, 7.2, 1.5 Hz, 1H), 7.37 – 7.28 (m, 6H), 7.28 – 7.23 (m, 1H), 7.23 – 7.17 (m, 6H), 2.69 (q, J = 7.7 Hz, 4H), 2.66 – 2.62 (m, 2H), 2.60 – 2.49 (m, 2H), 1.89 – 1.79 (m, 4H).

^{13}C{^{1}H} (101 MHz, $CDCl_3$) δ 161.9, 152.7, 150.0, 142.1, 141.2, 130.5, 128.7, 128.6, 128.6, 128.5, 126.4, 126.4, 126.0, 124.4, 124.2, 119.7, 117.1, 36.1, 36.0, 31.0, 30.7, 27.8, 27.3.

HRMS (ESI, m/z): $[M+H]^+$ calcd. for $C_{27}H_{27}O_2$, 383.2006; found 383.1999.

7,8,9,10,11,12,13,14,15,16-decahydro-6H-cyclododeca[c]chromen-6-one (3p)

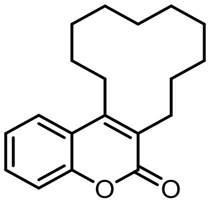

The title compound was synthesized according to general procedure 1 from 2-methoxybenzoyl chloride and cyclododecyne. Purification by preparative TLC (SiO_2, 5% EtOAc in hexane) afforded **3p** as a white solid (32.5 mg, 114 μmol, 38%).

^{1}H NMR (400 MHz, $CDCl_3$) δ 7.63 (dd, J = 8.0, 1.5 Hz, 1H), 7.44 (ddd, J = 8.5, 7.2, 1.5 Hz, 1H), 7.30 (dd, J = 8.3, 1.3 Hz, 1H), 7.29 – 7.23 (m, 1H), 2.96 – 2.81 (m, 2H), 2.81 – 2.58 (m, 2H), 1.86 – 1.70 (m, 4H), 1.65 – 1.41 (m, 12H).

^{13}C{^{1}H} (101 MHz, $CDCl_3$) δ 162.0, 152.6, 150.4, 130.3, 127.1, 125.1, 124.0, 120.1, 117.1, 27.5, 27.2, 27.1, 26.5, 26.3, 26.2, 26.1, 26.0, 22.5, 22.2.

HRMS (ESI, m/z): $[M+H]^+$ calcd. for $C_{19}H_{24}O_2$, 284.1771; found 284.1769.

3-butyl-4-phenyl-2H-chromen-2-one and 4-butyl-3-phenyl-2H-chromen-2-one (3q$^{\alpha}$ and 3q$^{\beta}$)

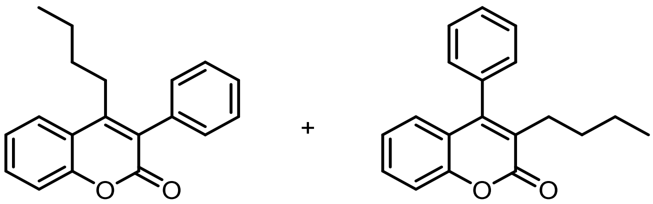

The title compound was synthesized according to general procedure 1 from 2-methoxybenzoyl chloride and hex-1-yn-1-ylbenzene. Purification by preparative TLC (SiO_2, 10% EtOAc in hexane) afforded **3q$^{\alpha}$** and **3q$^{\beta}$** as separable isomers. **3q$^{\alpha}$** was isolated as a yellow solid (36.7

mg, 132 μmol, 44%) and **3q**$^{\beta}$ as a yellow solid (25.6 mg, 92.0 μmol, 31%). (*combined yield: 62.3 mg, 224 μmol, 75%)*

3q$^{\alpha}$ (3-Ph isomer)

^{1}H NMR (400 MHz, $CDCl_3$) δ 7.68 (dd, *J* = 8.1, 1.5 Hz, 1H), 7.54 (ddd, *J* = 8.3, 7.3, 1.5 Hz, 1H), 7.50 – 7.44 (m, 2H), 7.43 – 7.37 (m, 2H), 7.32 (ddd, *J* = 8.0, 7.3, 1.3 Hz, 1H), 7.30 – 7.27 (m, 2H), 2.78 – 2.56 (m, 2H), 1.62 – 1.50 (m, 2H), 1.37 – 1.24 (m, 2H), 0.81 (t, *J* = 7.4 Hz, 3H).

^{13}C{^{1}H} (101 MHz, $CDCl_3$) δ 161.4, 153.4, 152.3, 134.7, 131.3, 129.8, 128.7, 128.3, 127.2, 125.3, 124.3, 119.7, 117.3, 32.0, 29.4, 23.0, 13.7.

HRMS (ESI, m/z): $[M+Na]^+$ calcd. For $C_{19}H_{18}NaO_2$, 301.1199; found 301.1206.

3q$^{\beta}$ (4-Ph isomer)

^{1}H NMR (500 MHz, $CDCl_3$) δ 7.56 – 7.48 (m, 3H), 7.44 (tt, *J* = 7.2, 1.4 Hz, 1H), 7.35 (dt, *J* = 8.3, 1.5 Hz, 1H), 7.25 – 7.22 (m, 1H), 7.11 (ddt, *J* = 8.3, 7.1, 1.2 Hz, 1H), 6.94 (dd, *J* = 7.9, 1.5 Hz, 1H), 2.41 – 2.29 (m, 2H), 1.57 – 1.39 (m, 2H), 1.21 (m, 2H), 0.81 – 0.70 (m, 3H).

^{13}C{^{1}H} (125 MHz, $CDCl_3$) δ 162.0, 152.7, 150.7, 135.0, 130.6, 128.9, 128.7, 128.4, 127.7, 127.3, 124.0, 121.1, 116.6, 31.1, 28.6, 22.8, 13.8.

HRMS (ESI, m/z): $[M+H]^+$ calcd. for $C_{19}H_{18}O_2$, 278.1301; found 278.1305.

Assignment of isomers was achieved through 2D NMR analysis and comparison with reference spectra.[3]

3-hexyl-2H-chromen-2-one and 4-hexyl-2H-chromen-2-one (3r$^{\alpha}$ and 3r$^{\beta}$)

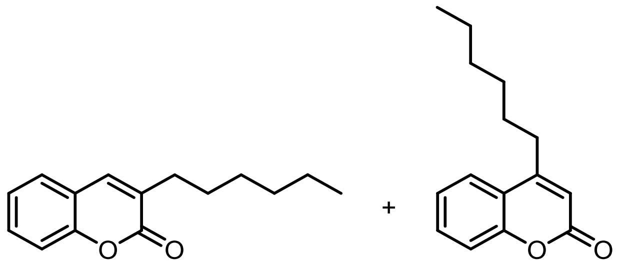

The title compound was synthesized according to general procedure 1 from 2-methoxybenzoyl chloride and hex-1-yn-1-ylbenzene. Purification by preparative TLC (SiO_2, 10% EtOAc in hexane) afforded **3r**$^{\alpha}$ and **3r**$^{\beta}$ as separable isomers. **3r**$^{\alpha}$ was further purified by another

preparative TLC (SiO_2, 100% DCM) and isolated as a yellow solid (16.6 mg, 72.1 µmol, 24%). **3r^β** was further purified by another preparative TLC (SiO_2, 70% DCM in hexane) and isolated as a yellow solid (17.8 mg, 77.3 µmol, 26%). (*combined yield: 34.4 mg, 149 µmol, 50%)*

3r^α (3-hexyl isomer)

^{1}H NMR (400 MHz, $CDCl_3$) δ 7.48 (d, *J* = 0.9 Hz, 1H), 7.44 (ddd, *J* = 7.8, 5.7, 1.4 Hz, 2H), 7.31 (ddd, *J* = 8.3, 1.2, 0.6 Hz, 1H), 7.27 – 7.21 (m, 1H), 2.65 – 2.43 (m, 2H), 1.73 – 1.57 (m, 2H), 1.42 – 1.26 (m, 6H), 0.93 – 0.85 (m, 3H).

^{13}C{^{1}H} (101 MHz, $CDCl_3$) δ 162.0, 153.3, 138.4, 130.6, 130.3, 127.2, 124.3, 119.8, 116.6, 31.8, 31.0, 29.1, 28.1, 22.7, 14.2.

HRMS (ESI, m/z): $[M+H]^+$ calcd. For $C_{15}H_{18}O_2$, 230.1301; found 230.1299.

3r^β (4-hexyl isomer)

^{1}H NMR (400 MHz, $CDCl_3$) δ 7.64 (dd, *J* = 8.0, 1.6 Hz, 1H), 7.52 (ddd, *J* = 8.7, 7.2, 1.6 Hz, 1H), 7.35 (dd, *J* = 8.4, 1.2 Hz, 1H), 7.29 (ddd, *J* = 8.3, 7.3, 1.2 Hz, 1H), 6.29 (d, *J* = 1.2 Hz, 1H), 2.83 – 2.69 (m, 2H), 1.78 – 1.63 (m, 2H), 1.44 (ddt, *J* = 8.7, 5.6, 1.5 Hz, 2H), 1.38 – 1.31 (m, 4H), 0.96 – 0.85 (m, 3H).

^{13}C{^{1}H} (101 MHz, $CDCl_3$) δ 161.2, 156.5, 153.9, 131.7, 124.5, 124.3, 119.5, 117.5, 114.1, 31.9, 31.7, 29.3, 28.3, 22.7, 14.2.

HRMS (ESI, m/z): $[M+Na]^+$ calcd. for $C_{15}H_{18}NaO_2$, 253.1199; found 253.1203.

Assignment of isomers was achieved through comparison with references in the literature.[4]

4-phenyl-3-(trimethylsilyl)-2H-chromen-2-one (3s)

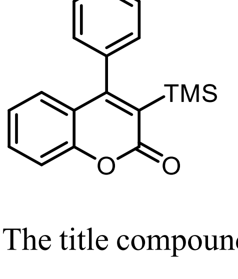

The title compound was synthesized according to general procedure 1 from 2-methoxybenzoyl chloride and phenylethynyl trimethylsilane. Purification by preparative TLC (SiO_2, 8% EtOAc in hexane) afforded **3s** as a white-yellow solid (42.9 mg, 146 µmol, 49%).

^{1}H NMR (400 MHz, $CDCl_3$) δ 7.52 – 7.43 (m, 4H), 7.32 (ddd, J = 8.3, 1.2, 0.5 Hz, 1H), 7.25 – 7.21 (m, 2H), 7.08 (ddd, J = 8.0, 7.2, 1.2 Hz, 1H), 6.94 (ddd, J = 8.0, 1.6, 0.5 Hz, 1H), -0.04 (s, 9H).

^{13}C{^{1}H} (101 MHz, $CDCl_3$) δ 163.3, 162.1, 154.0, 137.1, 131.7, 128.9, 128.9, 128.5, 127.6, 127.1, 123.7, 121.1, 116.6, 0.0.

HRMS (ESI, m/z): $[M+H]^+$ calcd. for $C_{18}H_{18}O_2Si$, 294.1071; found 294.1069.

Assignment of isomers was achieved through comparison of related compounds in the literature and characteristic proton shifts upon protodesilylation.[5]

***N*-(3-hexyl-2-oxo-2H-chromen-4-yl)-*N*,4-dimethylbenzenesulfonamide (3t)**

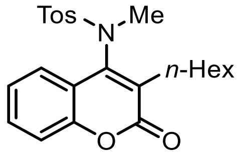

The title compound was synthesized according to general procedure 1 from 2-methoxybenzoyl chloride and N,4-dimethyl-N-(oct-1-yn-1-yl)benzenesulfonamide. Purification by preparative TLC (SiO_2, 20% EtOAc in hexane) afforded **3t** as a white-yellow solid (105 mg, 254 µmol, 85%).

^{1}H NMR (400 MHz, $CDCl_3$) δ 7.77 – 7.72 (m, 2H), 7.47 (ddd, J = 8.4, 7.3, 1.6 Hz, 1H), 7.38 – 7.32 (m, 4H), 7.18 (ddd, J = 7.9, 7.2, 1.1 Hz, 1H), 3.22 (s, 3H), 2.48 (d, J = 0.8 Hz, 3H), 2.36 (ddd, J = 13.0, 11.3, 4.8 Hz, 1H), 2.25 (ddd, J = 13.1, 11.4, 5.2 Hz, 1H), 1.71 – 1.56 (m, 1H), 1.49 – 1.36 (m, 1H), 1.32 – 1.14 (m, 6H), 0.87 (t, J = 7.1 Hz, 3H).

^{13}C{^{1}H} (101 MHz, $CDCl_3$) δ 162.2, 152.6, 147.3, 144.5, 136.4, 132.1, 131.3, 130.1, 127.7, 125.3, 124.4, 119.1, 116.9, 37.9, 31.5, 29.9, 28.4, 27.9, 22.7, 21.7, 14.2.

HRMS (ESI, m/z): $[M+H]^+$ calcd. for $C_{23}H_{28}NO_4S$, 414.1734; found 414.1732.

Assignment of isomers was achieved through 13C shift and 2D NMR analysis.

In situ acid chloride generation procedure

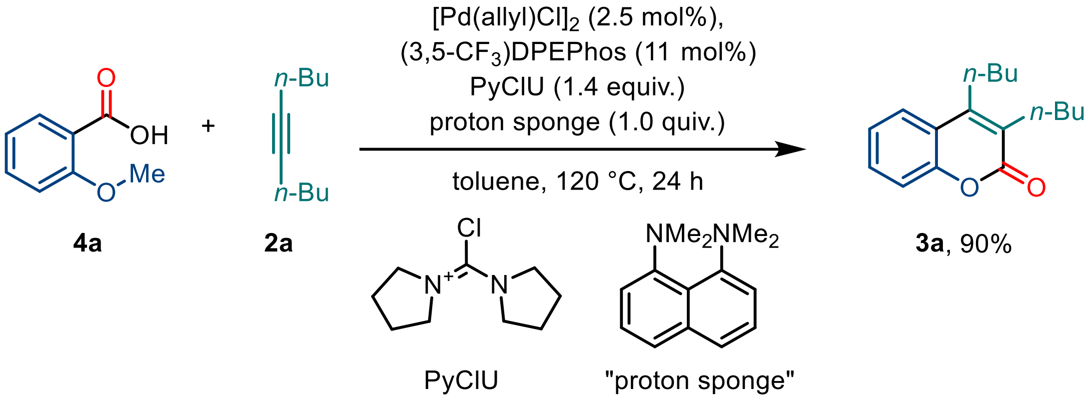

In a glovebox, to an oven dried 4 mL vial with a stir bar was added $[Pd(allyl)Cl]_2$ (2.74 mg, 7.50 µmol, 2.50 mol%,), followed by $3,5\text{-}CF_3DPEPhos$ (35.7 mg, 33 µmol, 11.0 mol%), and toluene (0.5 mL, 0.6 M). The mixture was stirred for 10 min before addition of alkyne (81.0 µL, 450 µmol, 1.50 equiv.), *o*-anisic acid (45.6 mg, 300 µmol, 1.00 equiv.), PyClU (140 mg, 420 µmol, 1.40 equiv.) and proton sponge (64.3 mg, 300 µmol, 1.00 equiv.) in that order. The vial was then capped and removed from the glovebox and placed in a preheated stir plate at 120 °C, 1000 rpm for 24h. The solution was quenched with MeOH and then filtered through a plug of silica with EtOAc and concentrated under reduced pressure. The product was then purified by preparative TLC (SiO_2, 5% EtOAc in hexane) to yield the corresponding coumarin **3a** as a yellow solid (70.1 mg, 271 µmol, 90%).

Protodesilylation

4-phenyl-2H-chromen-2-one (3s-desil)

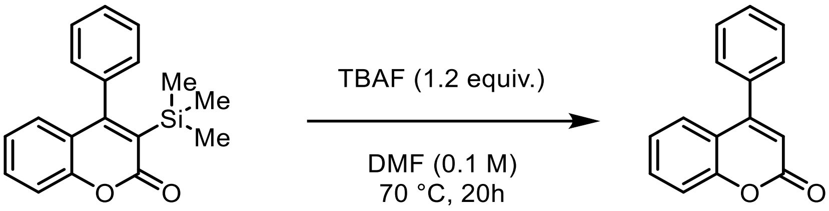

The employed procedure was adapted from the literature.[5]

To 4-phenyl-3-(trimethylsilyl)-2H-chromen-2-one (29.4 mg, 0.10 mmol, 1.00 equiv.) in DMF (1.0 mL) in a 4 mL drum vial under N_2 was added TBAF (1.0 M solution in THF, 0.12 mmol, 1.20 equiv.). The resulting mixture was heated to 70 °C for 20 h. Whenever the mixture turned from light-yellow to dark-brownish, a drop of acetic acid was added. Upon completion, volatiles were removed *in vacuo* and the title compound obtained after column chromatography (SiO_2, 6% EtOAc in hexane) as a white solid (18.3 mg, 82.3 µmol, 82%).

^{1}H NMR (400 MHz, $CDCl_3$) δ 7.61 – 7.44 (m, 7H), 7.42 (ddd, *J* = 8.3, 1.3, 0.5 Hz, 1H), 7.25 – 7.18 (m, 1H), 6.39 (s, 1H).

^{13}C{^{1}H} (101 MHz, $CDCl_3$) δ 155.8, 154.4, 135.4, 132.1, 129.8, 129.0, 128.6, 127.2, 124.3, 119.2, 117.5, 115.4.

The obtained analytical data was in accordance with reports in the literature.[5]

Starting material synthesis

General procedure 2

A 10 mL pointed flask equipped with a stirring bar was placed in a sand bath. The carboxylic acid (3.00 mmol, 1.00 equiv.) was added to the flask followed by thionyl chloride (2.20 mL, 30.0 mmol, 10.0 equiv.). The flask was connected to a straight drying tube stuffed with cotton and anhydrous calcium sulfate. The sand bath was heated at 60 °C, 400 rpm for 6h. The solution was concentrated under reduced pressure and used without further purification in the next step.

The acid chlorides **1a-1l** were all synthesized according to General Procedure 2.

Other compounds

2-methoxy-5-(4,4,5,5-tetramethyl-1,3,2-dioxaborolan-2-yl)benzoic acid (S1)

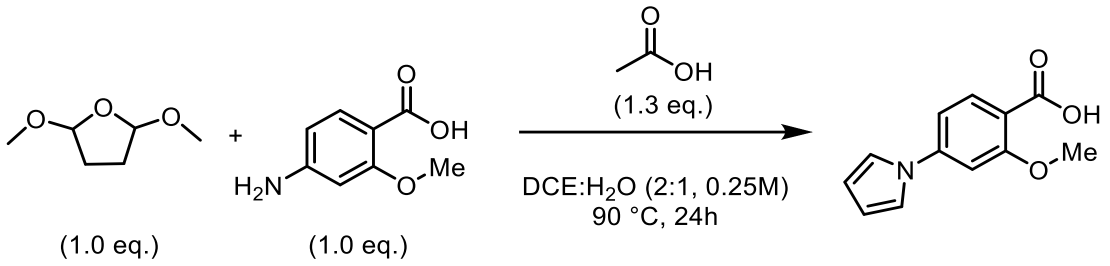

The procedure was adapted from the literature.[6]

To a solution of 4-amino-2-methoxybenzoic acid (836 mg, 5.00 mmol, 1.00 equiv.) in a 2:1 mixture of DCE (13 mL) and H_2O (6.7 mL) was added 2,5-dimethoxyoxolane (0.65 mL, 5.00 mmol, 1.00 equiv.). The resulting mixture was heated to 90 °C for 24 hours, after which the aqueous phase was extracted with DCM (3 × 15 mL) and the organic residues were dried over $MgSO_4$. Then, volatiles were removed *in vacuo*. Purification by column chromatography (SiO_2, 0 to 5% EtOAc gradient in hexane) afforded **S1** as a white solid (600 mg, 2.76 mmol, 55%).

^{1}H NMR (400 MHz, DMSO-d^6) δ 7.75 (d, J = 8.4 Hz, 1H), 7.51 (dt, J = 2.4, 1.4 Hz, 2H), 7.24 (d, J = 2.1 Hz, 1H), 7.21 (ddd, J = 8.5, 2.1, 1.1 Hz, 1H), 6.30 (t, J = 2.2 Hz, 2H), 3.91 (s, 3H).

^{13}C{^{1}H} (101 MHz, DMSO-d^6) δ 166.5, 159.9, 143.7, 132.7, 119.3, 117.1, 111.1, 110.3, 103.3, 56.1.

HRMS (ESI, m/z): $[M+H]^+$ calcd. for $C_{12}H_{12}NO_3$, 218.0812; found 218.0810.

2-methoxy-5-(4,4,5,5-tetramethyl-1,3,2-dioxaborolan-2-yl)benzoic acid (S2)

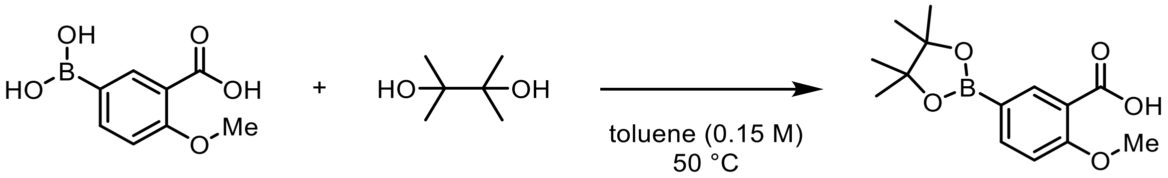

In a 100 mL RBF 5-(dihydroxyboryl)-2-methoxybenzoic acid (0.59 mg, 3.00 mmol, 1.00 equiv.) and pinacol (0.36 mg, 3.00 mmol, 1.00 equiv.) were added to toluene (20 mL). The flask was heated to 50 °C and concentrated under reduced pressure. Dissolving and removal of volatiles *in* vacuo was repeated two times, yielding the title product in quantitative yield as a white solid (834 mg, 3.00 mmol, 100%)

^{1}H NMR (500 MHz, $CDCl_3$) δ 8.65 (d, J = 1.7 Hz, 1H), 7.98 (dd, J = 8.3, 1.8 Hz, 1H), 7.04 (d, J = 8.3 Hz, 1H), 4.09 (s, 3H), 1.33 (s, 12H).

^{13}C{^{1}H} (125 MHz, $CDCl_3$) δ 165.4, 160.3, 141.7, 141.1, 117.2, 111.0, 84.2, 56.8, 25.0.

HRMS (ESI, m/z): $[M+Na]^+$ calcd. for $C_{14}H_{19}BNaO_5$, 301.1218; found 301.1222.

1,8-diphenyloct-4-yne (S3)

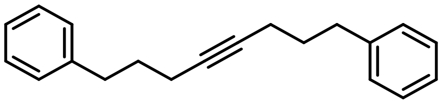

The title compound was synthesized according to a reported procedure.[7]

1,10-dichlorodec-5-yne (S4)

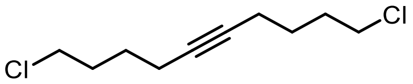

The title compound was synthesized according to a reported procedure.[8]

Mechanistic investigations

Reaction profile

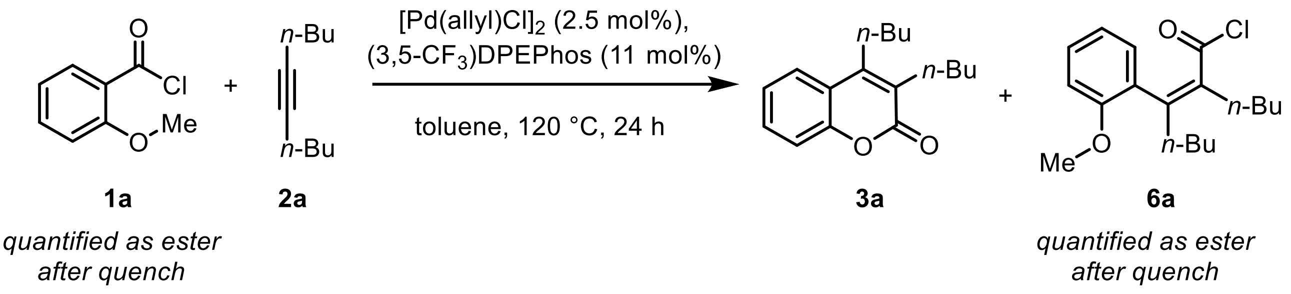

In a glovebox, to 20 oven dried 1 mL vials with a stir bar was added 3,5-CF_3DPEPhos (5.95 mg, 5.50 µmol, 11.0 mol%) followed by [Pd(allyl)Cl]$_2$ in toluene (0.6 M stock solution of 9.60 mg of [Pd] in 1.58 mL toluene, 75.1 µL per reaction, 1.25 µmol, 2.50 mol%). The mixtures were stirred for 10 min before addition of alkyne (13.5 µL,75.0 µmol, 1.50 equiv.) and acid chloride (6.90 µL, 50.0 µmol, 1.00 equiv.). The vials were then capped and removed from the glovebox and placed in a preheated stir plate at 120 °C, 1000 rpm for their corresponding times. Two samples were removed from the heat source together and placed into an ice-bath to stop the reaction from proceeding. Subsequently, the solutions were quenched with MeOH containing trimethoxybenzene (0.2 M stock solution in MeOH; 2.80 mg, 16.7 µmol, 0.33 equiv. per reaction) as internal standard and then filtered through a plug of silica with EtOAc and concentrated under reduced pressure. Yields were determined by quantitative NMR.

Kinetic modeling:

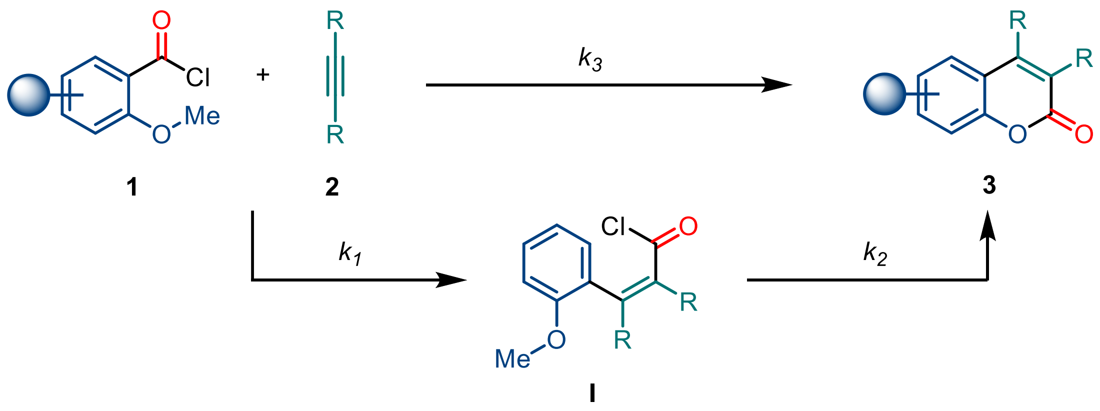

To elaborate on a direct conversion of the acid chloride with an alkyne to the coumarin vs. a prerequisite intermediary formation of an *ortho*-methoxy cinnamic acid chloride, we ran kinetic modeling experiments according to the reaction scheme above.

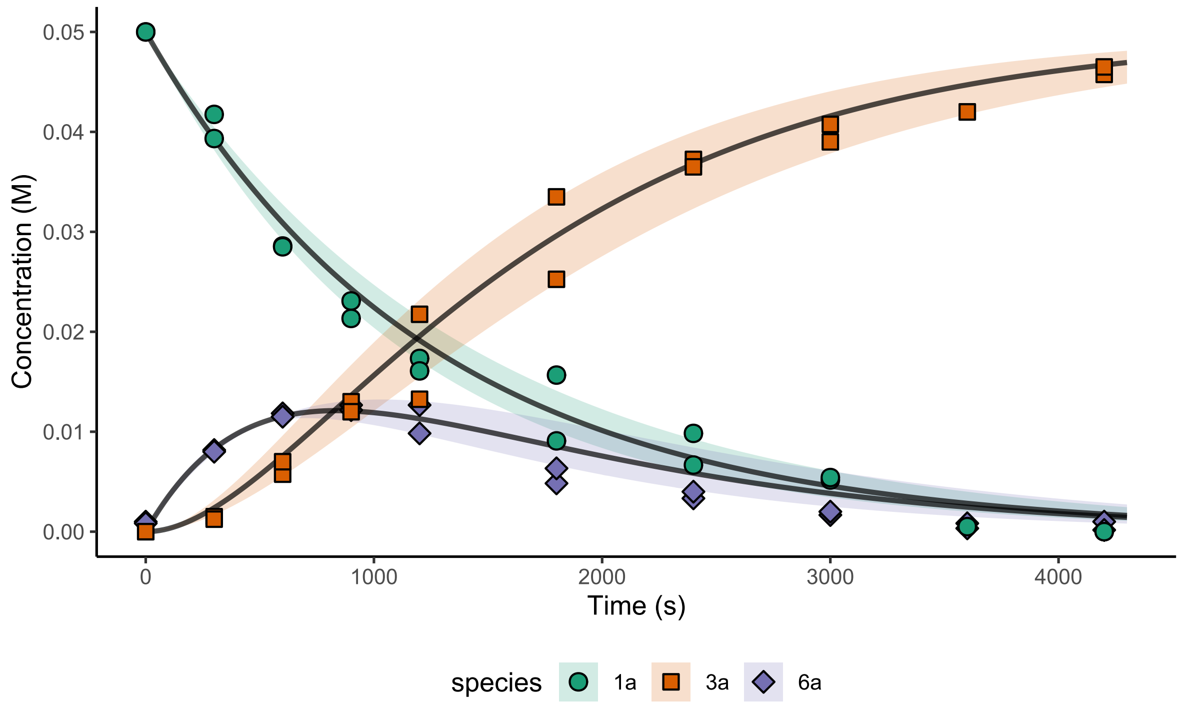

Figure S1: Plot of concentration in M against time in s, representing **1a** in green, **3a** in orange and **6a** in purple. Data points are derived from two independent experiments. The black curve represents the predictions of the model using mean values of k_1 and k_2 obtained from individual fits of the two replicates. The ribbons represent the bounds of the predictions derived from the 99.7% confidence interval of k_1 and k_2.

The following ordinary differential equation model was used for the kinetic data:

$$\mathbf{1a} \overset{k_1}{\rightarrow} \mathbf{6a} \overset{k_2}{\rightarrow} \mathbf{3a}$$

$$\frac{d[\mathbf{1a}]}{dt} = -k_1[\mathbf{1a}]$$

$$\frac{d[\mathbf{6a}]}{dt} = k_1[\mathbf{1a}] - k_2[\mathbf{6a}]$$

$$\frac{d[\mathbf{3a}]}{dt} = k_2[\mathbf{6a}]$$

The data from each replicate were fitted to the model by optimising the values of k_1 and k_2 such that the square of the residuals for all species is minimized. The mean value for k_1 and k_2 across replicates and the corresponding propagated standard error is $8.022 \cdot 10^{-4} \pm 3.236 \cdot 10^{-4}$ and $1.688 \cdot 10^{-3} \pm 1.344 \cdot 10^{-4}$ respectively.

The induction period observed for the formation of **3a** indicates that conversion of **1a** to **3a** directly must either not occur or occur with a rate that is several orders of magnitude slower than the pathway through a carbochlorocarbonylation intermediate **I** ($k_3 << k_1+k_2$).

However, it must be noted that this is not sufficient evidence to unambigously confirm initial formation of a carbochlorocarbonylation intermediate followed by Lewis acid catalyzed annulation to the coumarin. Instead, reversible formation of the pallada-acyl species **II** might be feasible. The obtained data is not suited to differentiate fully between a direct pathway through a carbochlorocarbonylation intermediate (**II** → **I** (k_1); **I** → coumarin (k_2)) or the alternative reversible formation of **II** from **I** (k_{-1}) followed by conversion of **II** to the coumarin (k_3). For the latter case the observed onset period might originate from a favorable formation of **I** from **II** ($k_1 > k_{-1} > k_3$)

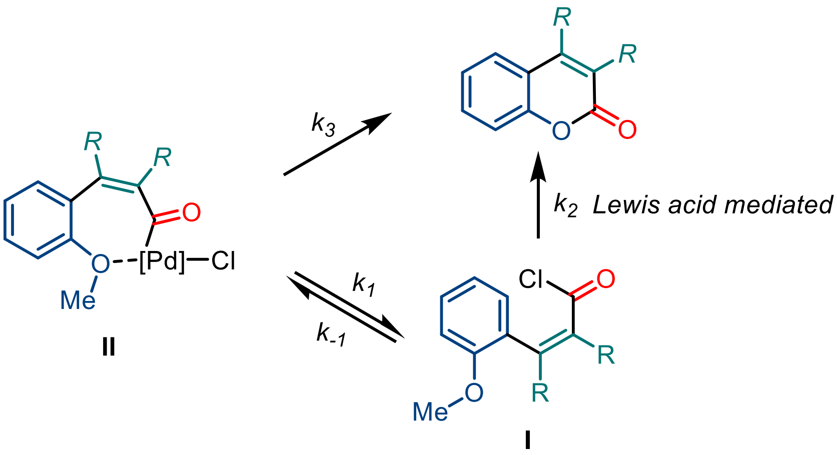

Methyl (Z)-2-butyl-3-(2-methoxyphenyl)hept-2-enoate (6a)

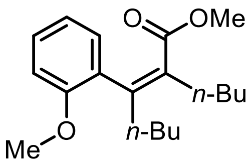

The title compound was synthesized according to general procedure 1 from 2-methoxy benzoyl chloride and 5-decyne, after reduced reaction time of 20 min instead of the usual 24 . Purification by column chromatography (SiO_2, 0 to 4% EtOAc in hexane) afforded the title compound as a colorless oil (9.90 mg, 33.0 µmol, 11%).

^{1}H NMR (500 MHz, $CDCl_3$) δ 7.23 – 7.18 (m, 1H), 6.93 – 6.90 (m, 1H), 6.89 – 6.83 (m, 2H), 3.79 (s, 3H), 3.33 (s, 3H), 2.43 (dt, J = 12.2, 7.4 Hz, 4H), 1.49 – 1.34 (m, 4H), 1.33 – 1.23 (m, 4H), 0.94 (t, J = 7.2 Hz, 3H), 0.84 (dd, J = 7.5, 6.5 Hz, 3H).

^{13}C{^{1}H} (125 MHz, $CDCl_3$) δ 170.7, 156.4, 145.3, 132.1, 131.9, 129.3, 128.2, 120.2, 110.9, 55.8, 51.1, 33.4, 31.5, 30.2, 29.6, 22.9, 22.8, 14.1, 14.1.

HRMS (ESI, m/z): $[M+H]^+$ calcd. for $C_{19}H_{29}O_3$, 305.2111; found 305.2109.

Ring closure from cinnamoyl chlorides

3-(2-methoxyphenyl)but-2-enoic acid (7u)

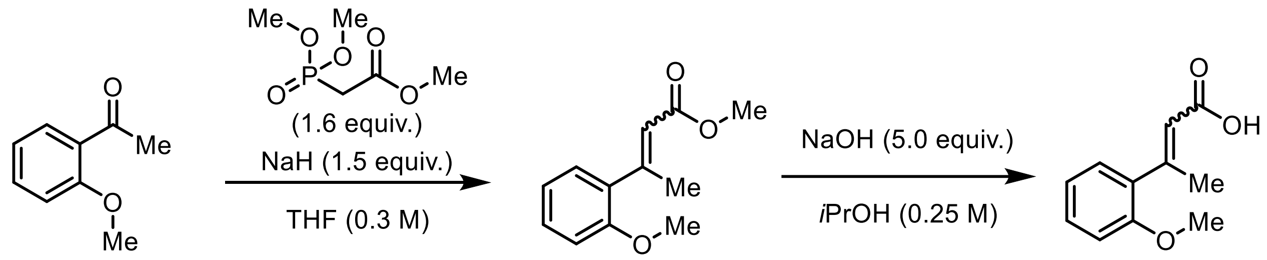

The employed procedure was adapted from the literature.[9]

To a 100 mL round bottom flask containing NaH (60% NaH in mineral oil, 600 mg, 15.0 mmol, 1.50 equiv.) and anhydrous THF (33 mL) at 0 ºC, was added trimethyl phosphonoacetate (2.47 mL, 16.0 mmol, 1.60 equiv.) dropwise. The reaction mixture was warmed to room temperature, followed by dropwise addition of 2-methoxy acetophenone (1.38 mL, 10.0 mmol, 1.00 equiv.). The reaction mixture was stirred for 12 hours and then quenched through the addition of water. The organic layer was collected, and the aqueous layer extracted with Et_2O (3 × 30 mL). The combined organic phases were dried over $MgSO_4$, filtered, and concentrated under reduced pressure. Purification by column chromatography (SiO_2, 0 to 5% EtOAc gradient in hexane) afforded a mixture of ester isomers as a yellow oil in sufficient purity and estimated quantitative yield to directly proceed with the next step.

The obtained ester was subjected to hydrolysis in *i*PrOH (40 mL) with NaOH (2.00 g, 50.0 mmol). The corresponding reaction mixture was heated to 70 °C for 2 h. Subsequently, water was added and the aqueous phase extracted with Et_2O (2 × 15 mL). The remaining aqueous phase was acidified through the addition of 6 M HCl and then extracted again with EtOAc (3 × 30 mL). Upon removal of volatiles *in vacuo*, the title mixture of acid isomers (1:1.1 mixture of isomers) was obtained as white-yellow solids (1.65 g, 8.58 mmol, 86% over two steps).

(obtained isomers differentiated by integration and 2D NMR analysis)

Isomer A:

^{1}H NMR (500 MHz, $CDCl_3$) δ 7.37 – 7.29 (m, 1H), 7.07 (dd, J = 7.4, 1.9 Hz, 1H), 7.01 – 6.83 (m, 2H), 6.00 (q, J = 1.4 Hz, 1H), 3.82 (s, 3H), 2.20 (d, J = 1.4 Hz, 3H).

$^{13}C\{^1H\}$ (125 MHz, $CDCl_3$) δ 170.9, 156.4, 155.7, 129.8, 129.1, 128.1, 120.6, 118.4, 111.0, 55.6, 26.7.

Isomer B:

^1H NMR (500 MHz, $CDCl_3$) δ 7.37 – 7.29 (m, 1H), 7.17 (dd, J = 7.5, 1.8 Hz, 1H), 7.01 – 6.83 (m, 2H), 5.94 (q, J = 1.4 Hz, 1H), 3.85 (s, 3H), 2.51 (d, J = 1.4 Hz, 3H).

$^{13}C\{^1H\}$ (125 MHz, $CDCl_3$) δ 172.2, 159.8, 155.4, 133.0, 129.9, 128.9, 120.7, 118.7, 111.2, 55.6, 20.4.

HRMS (ESI, m/z): $[M+H]^+$ calcd. for $C_{11}H_{12}O_3$, 192.0781; found 192.0779.

Ring closure from 7u

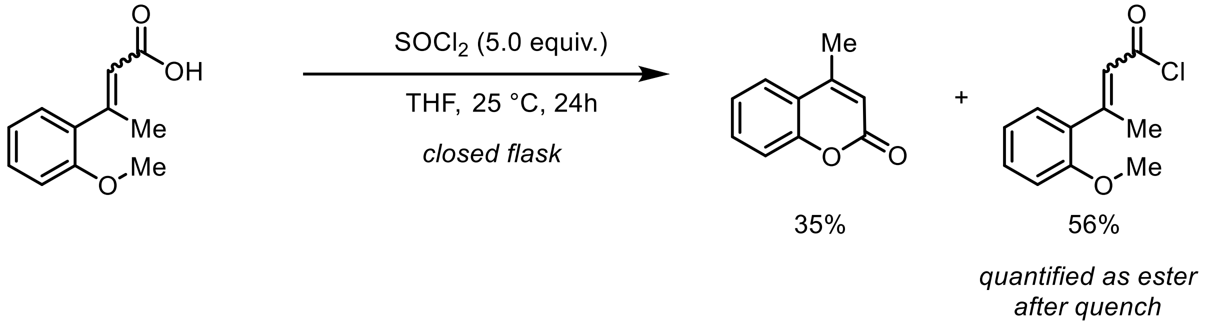

To an oven dried 4 mL vial with a stir bar was added acid **6t** (19.2 mg, 0.10 mmol, 1.00 equiv.), THF (0.4 mL) and thionyl chloride (36.5 µL, 0.50 mmol, 5.00 equiv.). The vial was closed immediately and subsequently stirred for 24 h at 25 °C. Subsequently, the solution was quenched with MeOH containing trimethoxybenzene (0.2 M stock solution in MeOH; 2.80 mg, 33.3 µmol, 0.33 equiv. per reaction) as internal standard and then filtered through a plug of silica with EtOAc and concentrated under reduced pressure. Yields were determined by quantitative ^1H-NMR.

Coumarin formation in presence of base

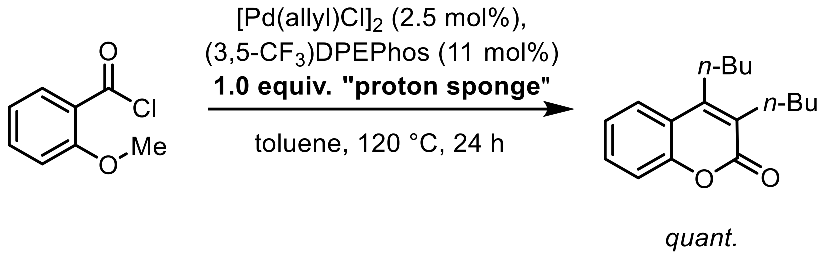

The reaction was conducted according to general procedure 1 on a 0.10 mmol scale, with the addition of proton sponge (21.4 mg, 0.10 mmol, 1.00 equiv.). Upon completion the solution was quenched with MeOH containing trimethoxybenzene (0.2 M stock solution in MeOH; 2.80 mg, 33.3 µmol, 0.33 equiv. per reaction) as internal standard and then filtered through a plug of silica with EtOAc and concentrated under reduced pressure. Yields were determined by quantitative ^{1}H-NMR.

Alkyl fate

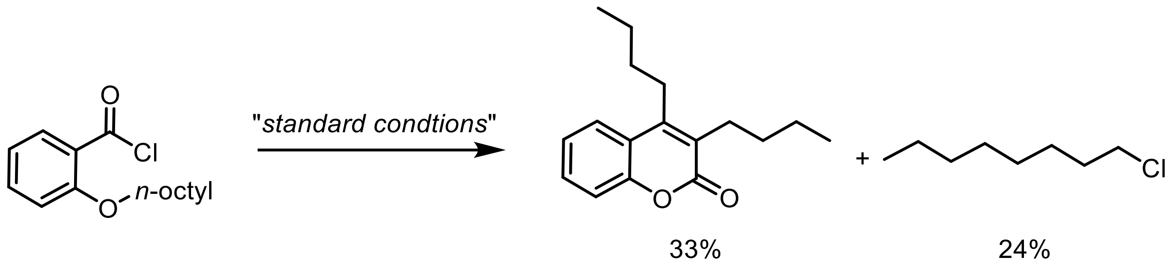

General procedure 1 was followed with 2-(octyloxy)benzoyl chloride **8a** (26.9 mg, 10.0 µmol, 1.00 equiv.) over 72h. The yield of both products was determined using 1,3,5-trimethoxybenzene (0.033 mmol, 0.33 equiv.) as a reference.

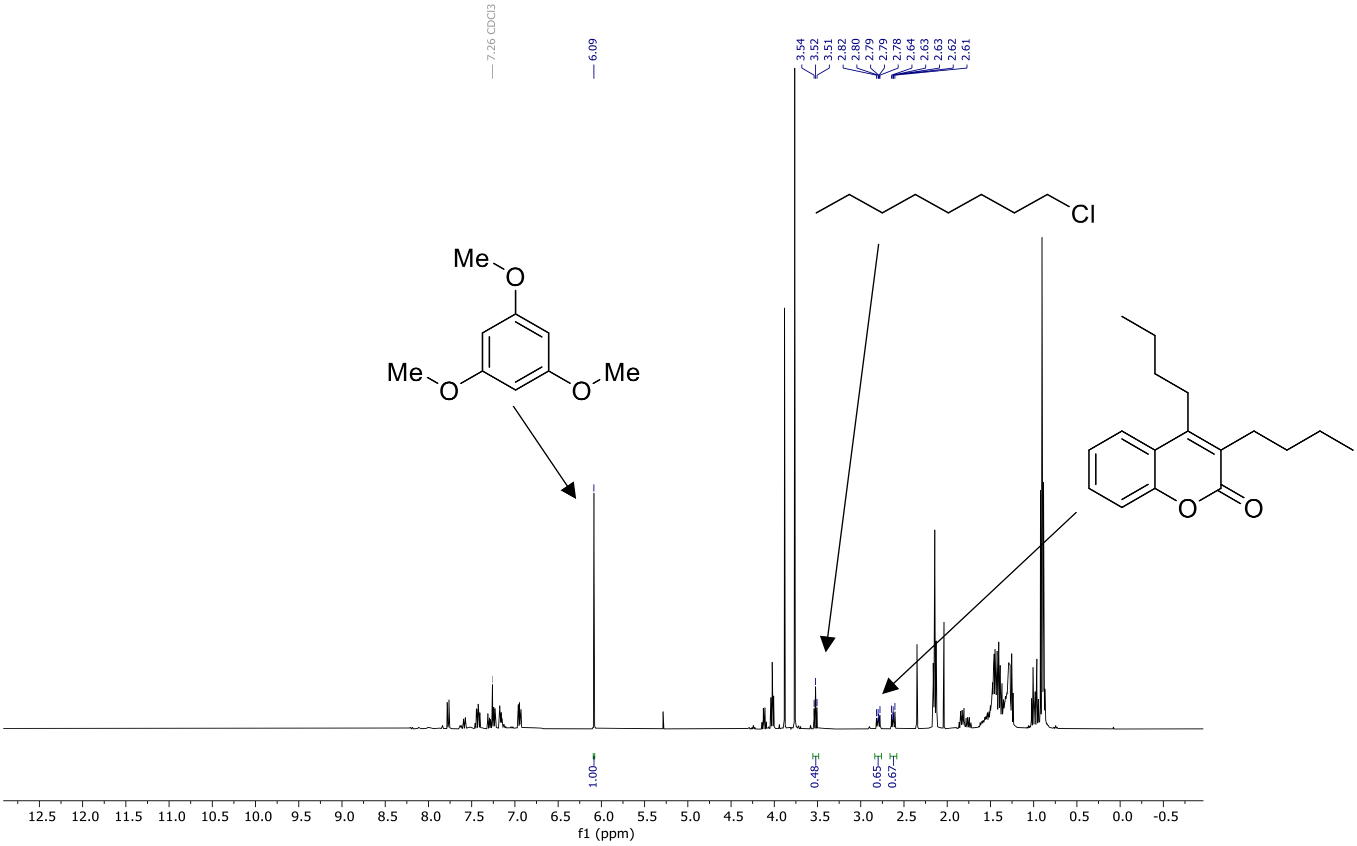

Figure S1: ^{1}H NMR (400 MHz, $CDCl_3$) The peaks 2.80 (m) and 2.62 (m) are both from the 3,4-dibutyl-2H-chromen-2-one, while the 3.52 (t) peak originates from the 1-chlorooctan byproduct.

References

(1) Denton, E.; Schmitt, H.; Stepanovic, O.; Müller, P.; Müller, A.; Svoboda, D.; Morandi, B. Ligand Design Enables the Palladium-Catalyzed Intermolecular Carbochlorocarbonylation of Alkynes and Cyclopentenone Formation. *ChemRxi* **2025**. https://doi.org/10.26434/chemrxiv-2025-vpptw.

(2) Zhao, Y.; Han, F.; Yang, L.; Xia, C. Access to Coumarins by Rhodium-Catalyzed Oxidative Annulation of Aryl Thiocarbamates with Internal Alkynes. *Org. Lett.* **2015**, *17* (6), 1477–1480. https://doi.org/10.1021/acs.orglett.5b00364.

(3) Manna, S.; Prabhu, K. R. Visible-Light-Mediated Vicinal Difunctionalization of Activated Alkynes with Boronic Acids: Substrate-Controlled Rapid Access to 3-Alkylated Coumarins and Unsaturated Spirocycles. *Org. Lett.* **2023**, *25* (5), 810–815. https://doi.org/10.1021/acs.orglett.2c04333.

(4) Gärtner, D.; Stein, A. L.; Grupe, S.; Arp, J.; Jacobi von Wangelin, A. Iron-Catalyzed Cross-Coupling of Alkenyl Acetates. *Angew. Chem. Int. Ed.* **2015**, *54* (36), 10545–10549. https://doi.org/10.1002/anie.201504524.

(5) Ladumor, R.; Samanta, S.; Selvakumar, S. Photocatalytic Silylation of Aryl Alkynoates: Synthesis of Silylated Coumarins. *Adv. Synth. Catal.* **2025**, *367* (3), 1–7. https://doi.org/10.1002/adsc.202401007.

(6) Romagnoli, R.; Oliva, P.; Salvador, M. K.; Manfredini, S.; Padroni, C.; Brancale, A.; Ferla, S.; Hamel, E.; Ronca, R.; Maccarinelli, F.; Rruga, F.; Mariotto, E.; Viola, G.; Bortolozzi, R. A Facile Synthesis of Diaryl Pyrroles Led to the Discovery of Potent Colchicine Site Antimitotic Agents. *Eur. J. Med. Chem.* **2021**, *214*, 113229. https://doi.org/10.1016/j.ejmech.2021.113229.

(7) Kawasaki, Y.; Ishikawa, Y.; Igawa, K.; Tomooka, K. Directing Group-Controlled Hydrosilylation: Regioselective Functionalization of Alkyne. *J. Am. Chem. Soc.* **2011**, *133* (51), 20712–20715. https://doi.org/10.1021/ja209553f.

(8) Lee, Y. H.; Denton, E. H.; Morandi, B. Modular Cyclopentenone Synthesis through the Catalytic Molecular Shuffling of Unsaturated Acid Chlorides and Alkynes. *J. Am. Chem. Soc.* **2020**, *142* (50), 20948–20955. https://doi.org/10.1021/jacs.0c10832.

(9) Jin, J.; Xu, Q.; Deng, W. <scp>DMAP</Scp> -Catalyzed [4 + 2] Cycloaddition of α , β -Unsaturated Carboxylic Acids with Ketones for Synthesis of α , β -Unsaturated δ -Lactones. *Chinese J. Chem.* **2017**, *35* (4), 397–400. https://doi.org/10.1002/cjoc.201600929.

NMR spectra

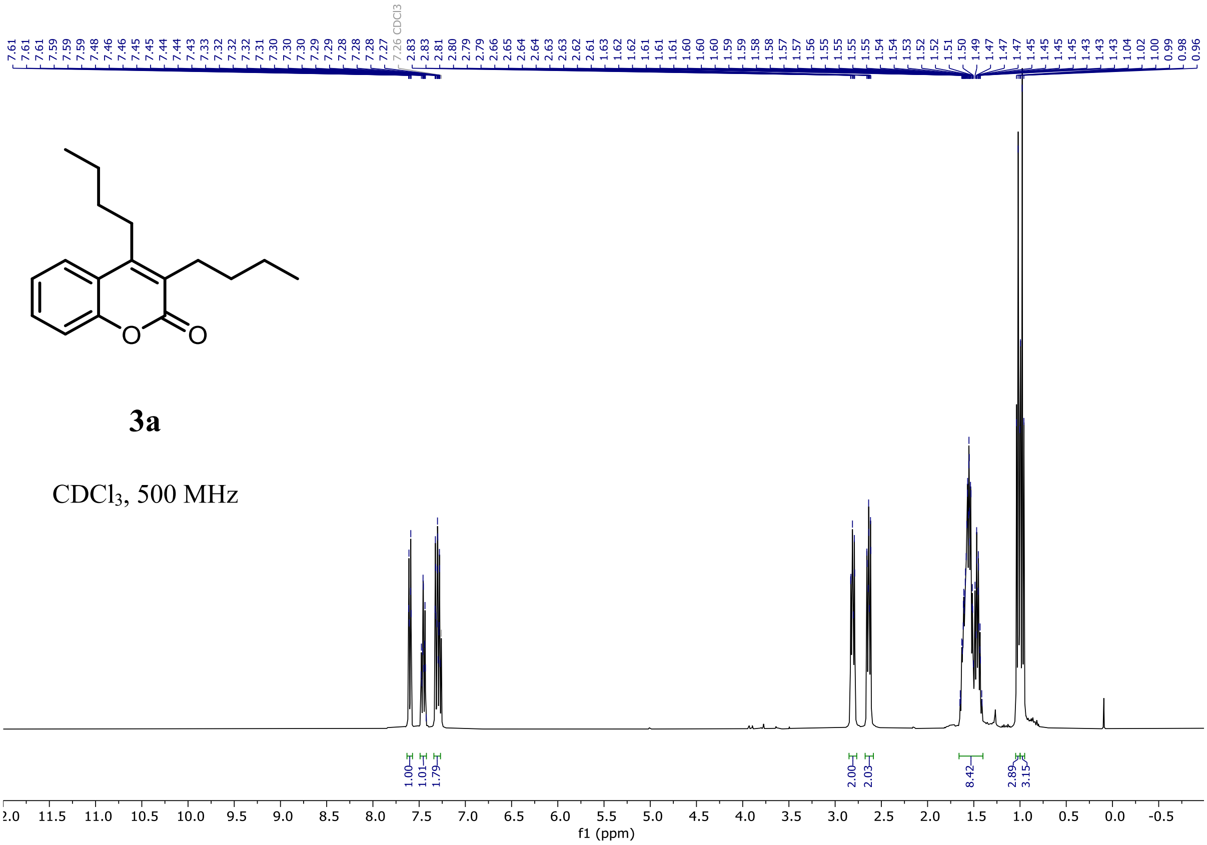
7.61 7.61 7.61 7.59 7.59 7.59 7.48 7.46 7.46 7.45 7.45 7.44 7.43 7.33 7.32 7.32 7.32 7.31 7.30 7.30 7.29 7.29 7.28 7.28 7.28 7.27 7.26 CDCl3 2.83 2.83 2.81 2.80 2.79 2.79 2.66 2.65 2.64 2.64 2.63 2.62 2.61 1.63 1.62 1.62 1.61 1.61 1.61 1.60 1.60 1.59 1.59 1.58 1.58 1.57 1.57 1.56 1.55 1.55 1.55 1.55 1.54 1.54 1.53 1.52 1.52 1.51 1.50 1.49 1.47 1.47 1.47 1.45 1.45 1.45 1.45 1.43 1.43 1.43 1.04 1.02 1.00 0.99 0.98 0.96
3a
CDCl3, 500 MHz
1.00 1.01 1.79 2.00 2.03 8.42 2.89 3.15
f1 (ppm)

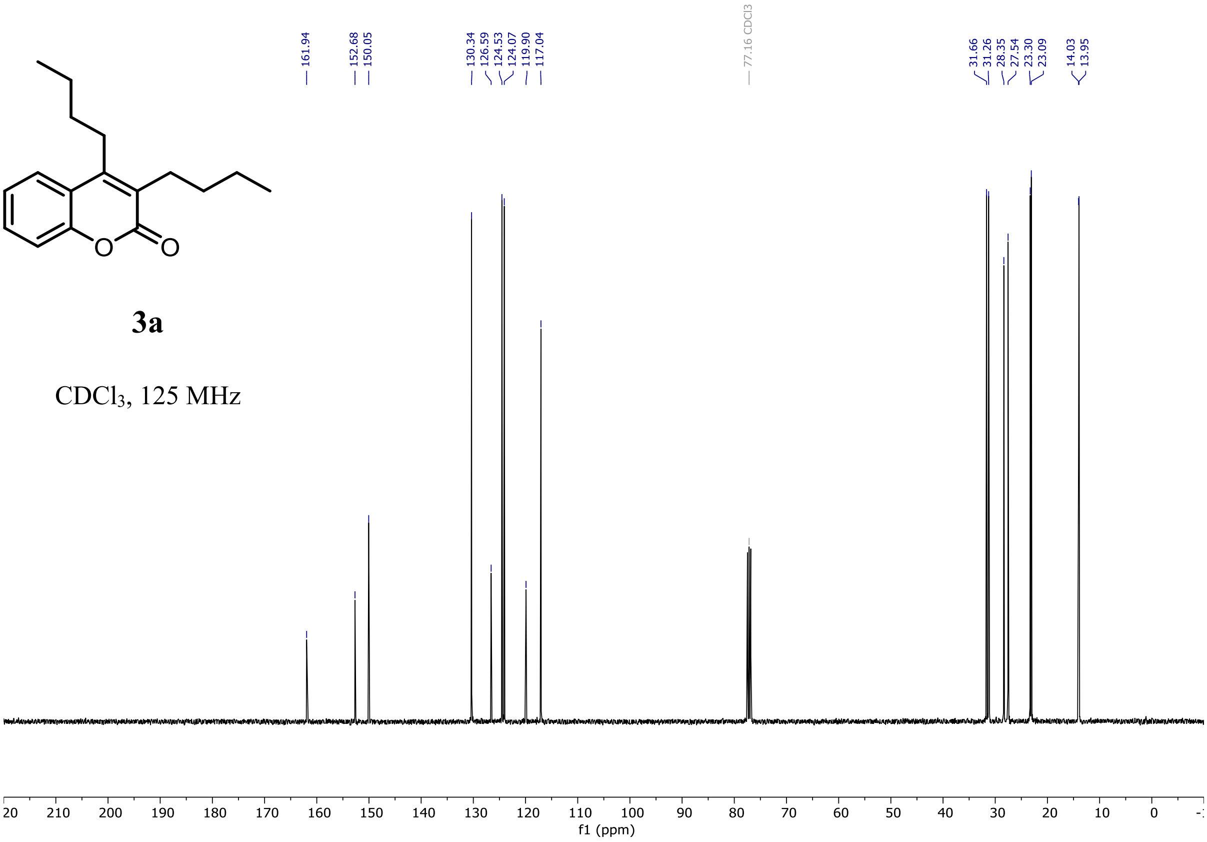
161.94 152.68 150.05 130.34 126.59 124.53 124.07 119.90 117.04 77.16 CDCl3 31.66 31.26 28.35 27.54 23.30 23.09 14.03 13.95
3a
CDCl3, 125 MHz
f1 (ppm)

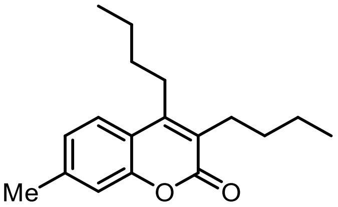

3b

$CDCl_3$, 400 MHz

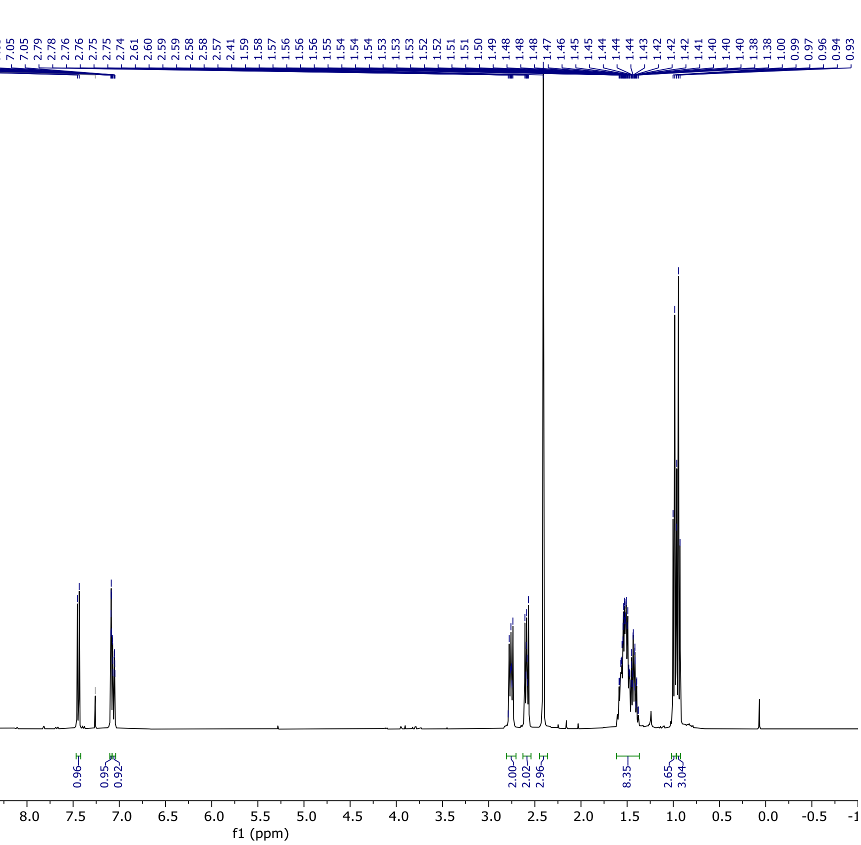

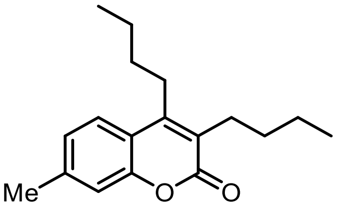

3b

$CDCl_3$, 101 MHz

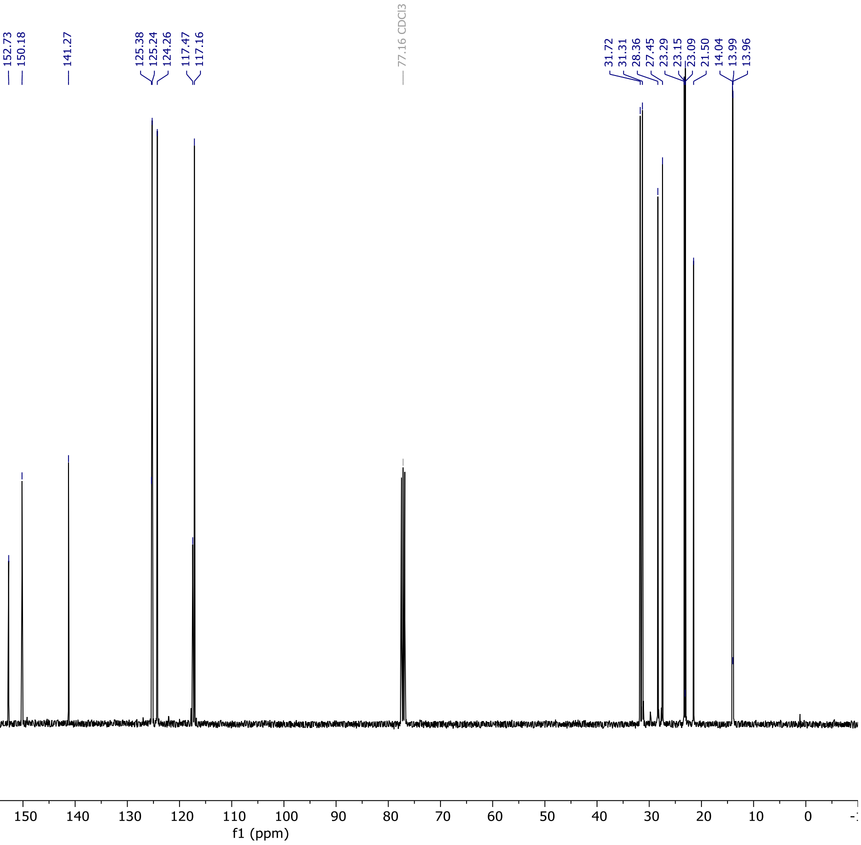

3c

CDCl$_3$, 400 MHz

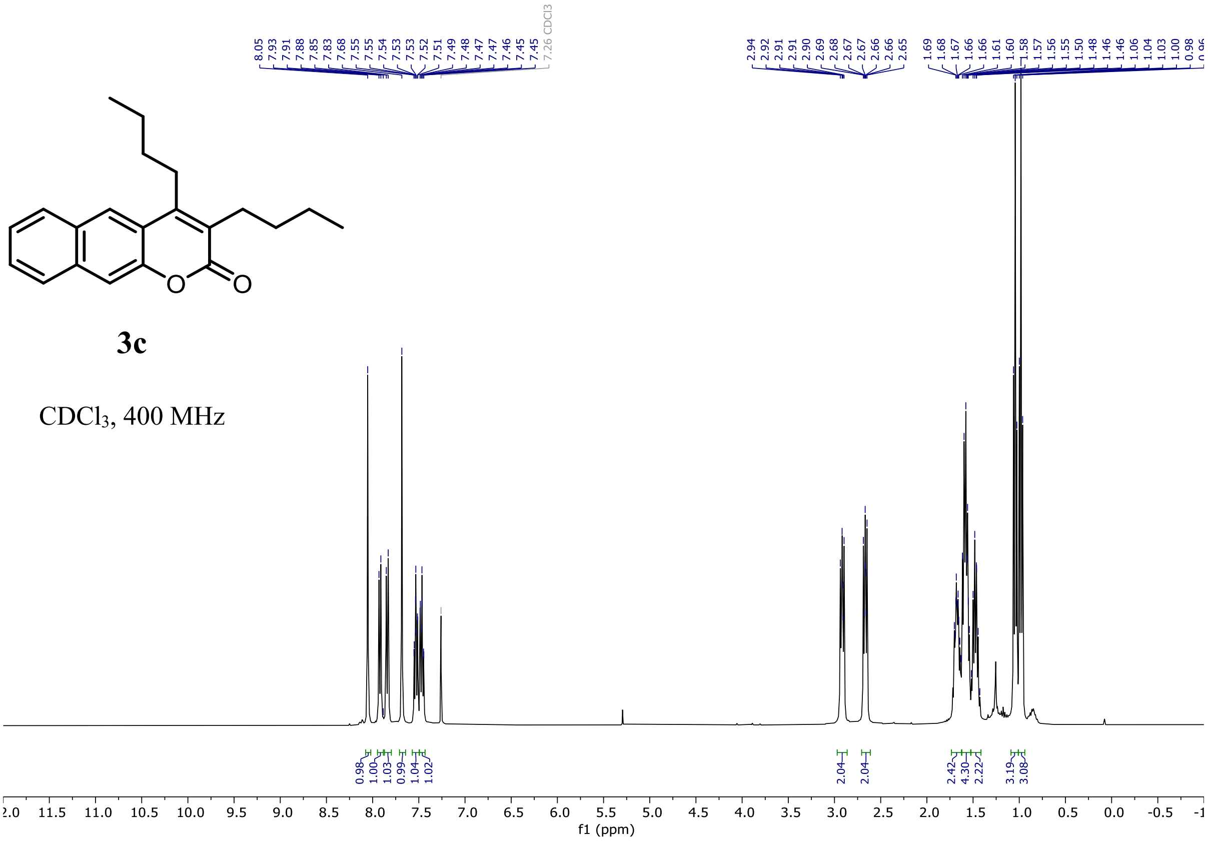

3c

CDCl$_3$, 101 MHz

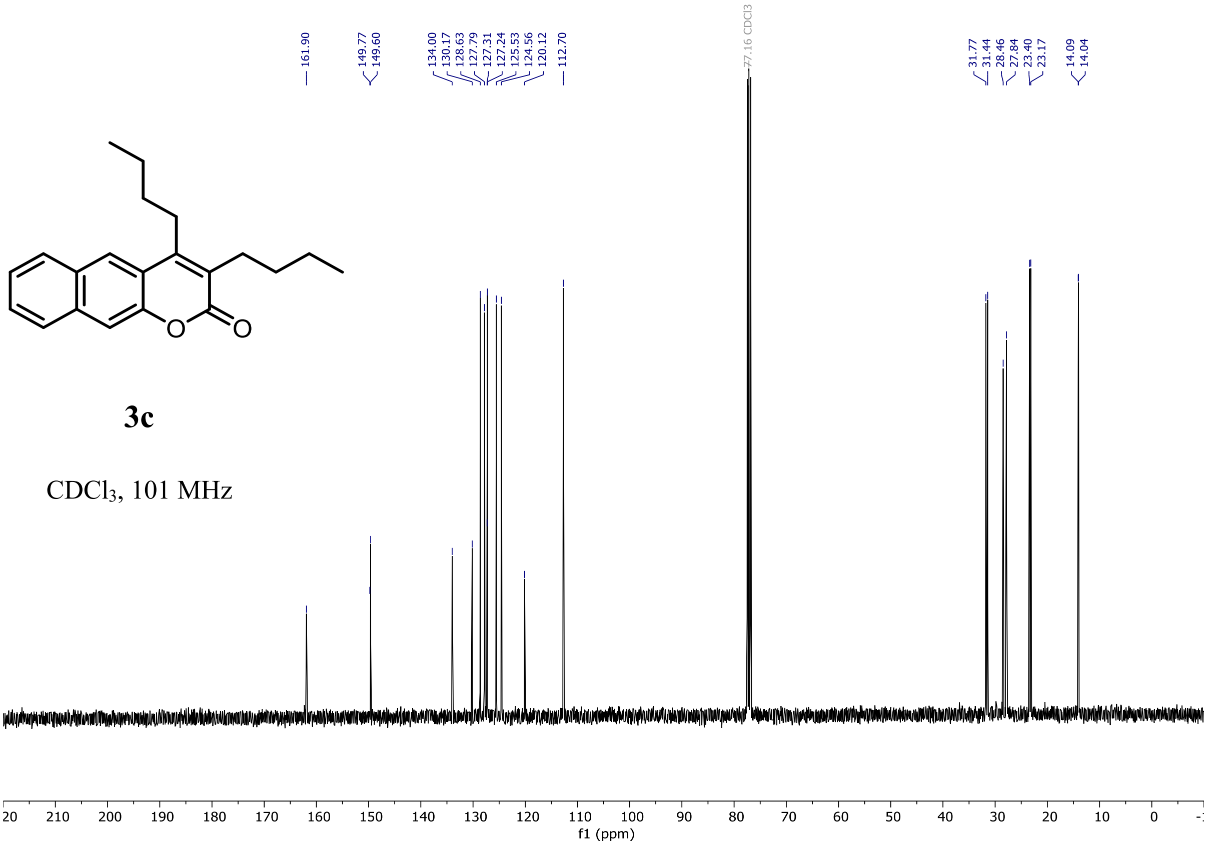

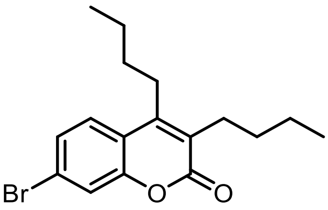

3d

$CDCl_3$, 400 MHz

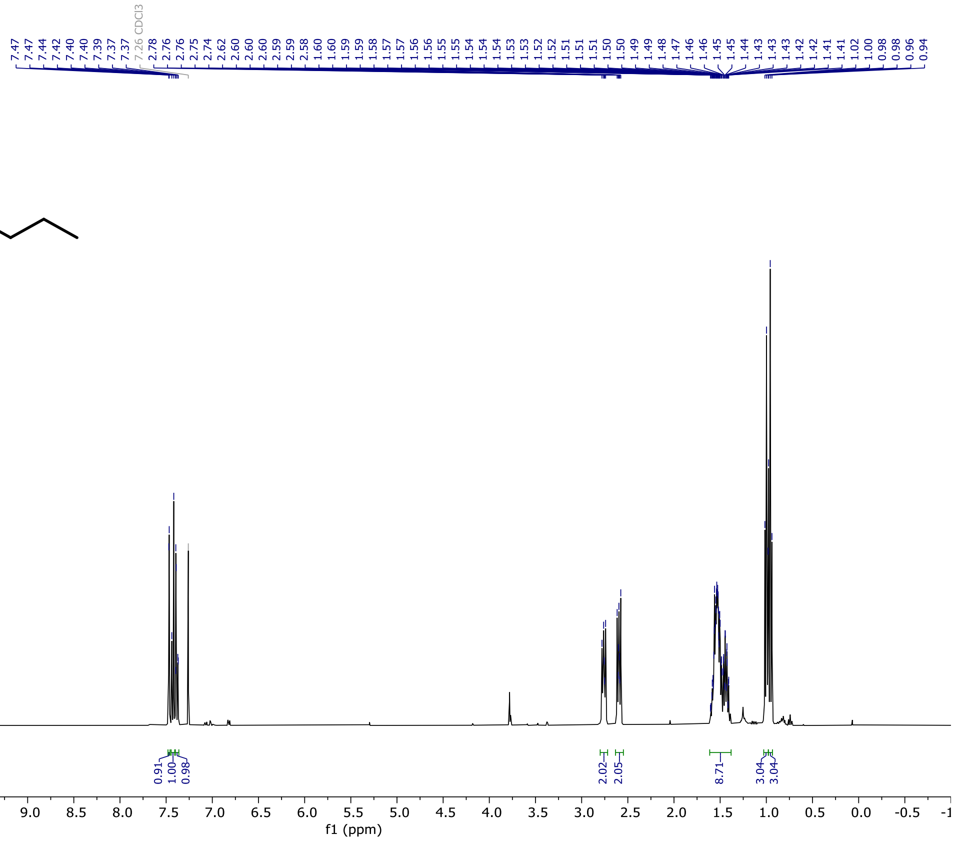

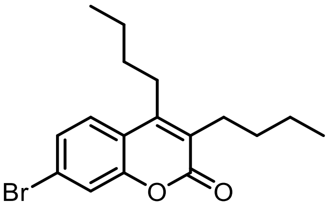

3d

$CDCl_3$, 101 MHz

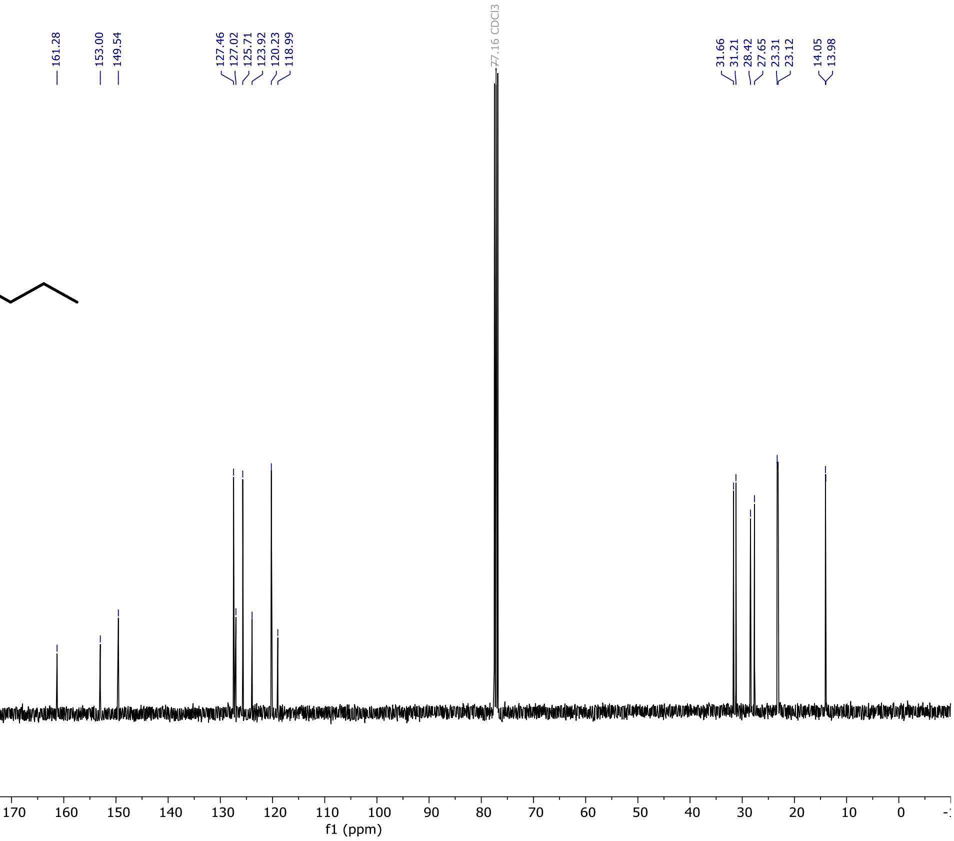

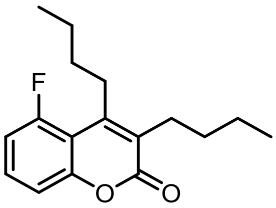

3e

$CDCl_3$, 500 MHz

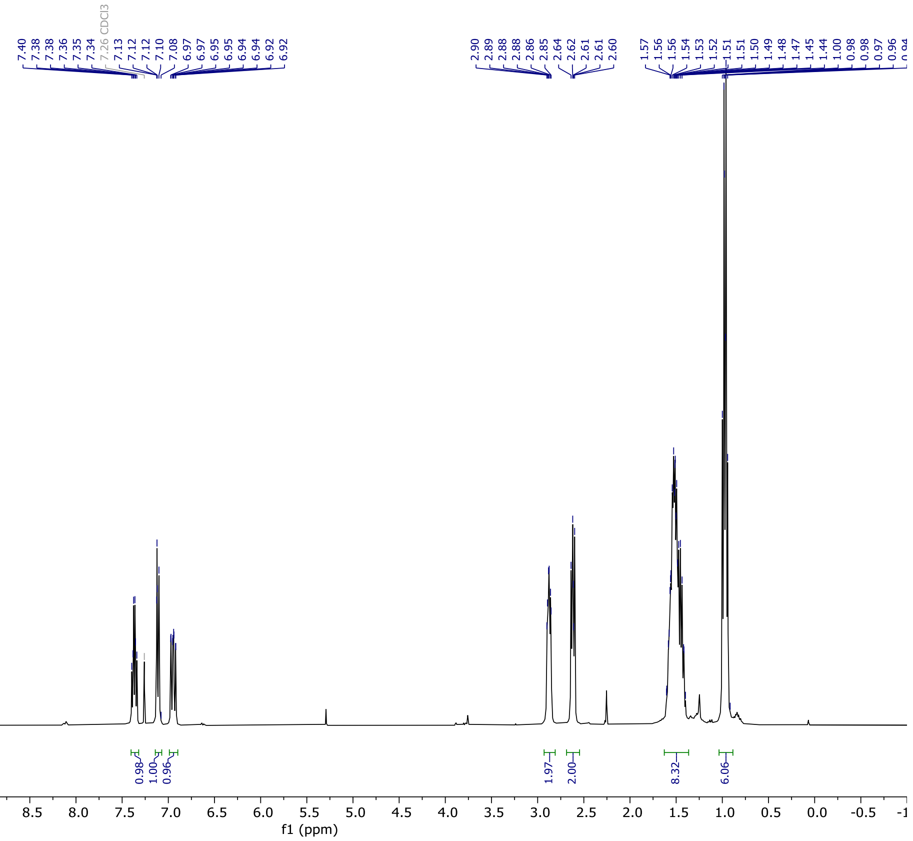

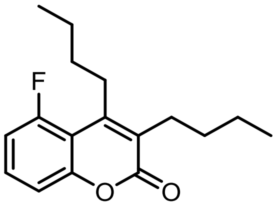

3e

$CDCl_3$, 125 MHz

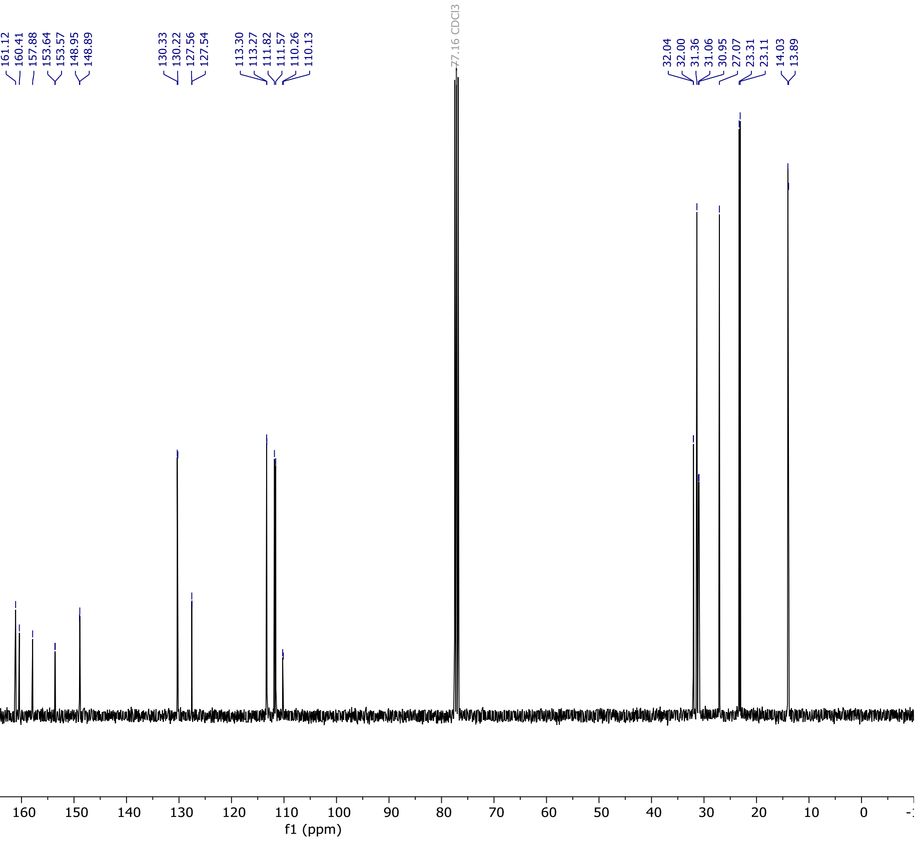

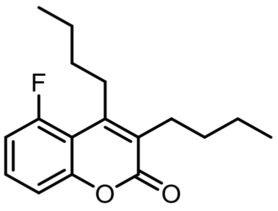

3e

CDCl$_3$, 376 MHz

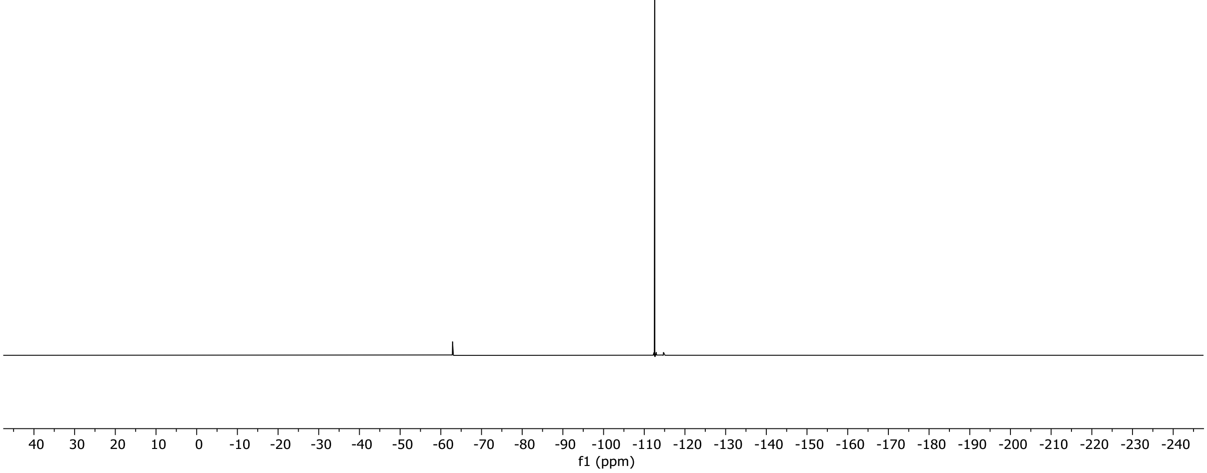

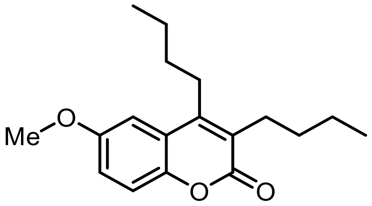

3f

CDCl$_3$, 400 MHz

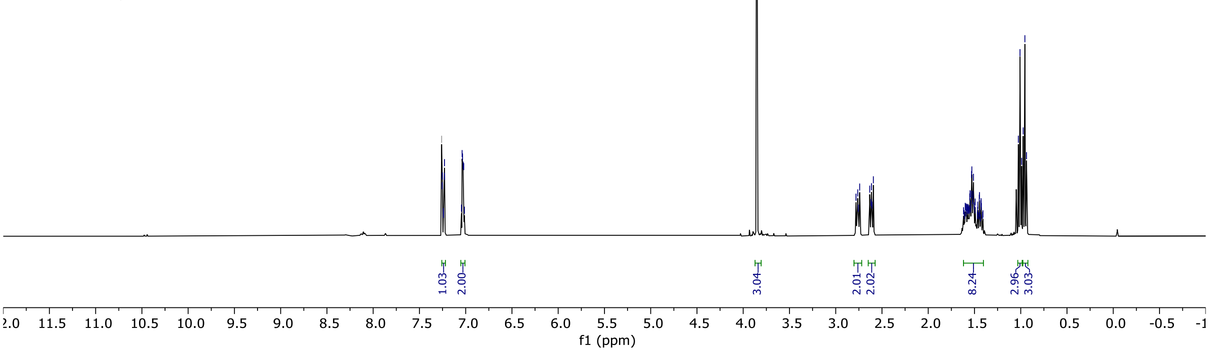

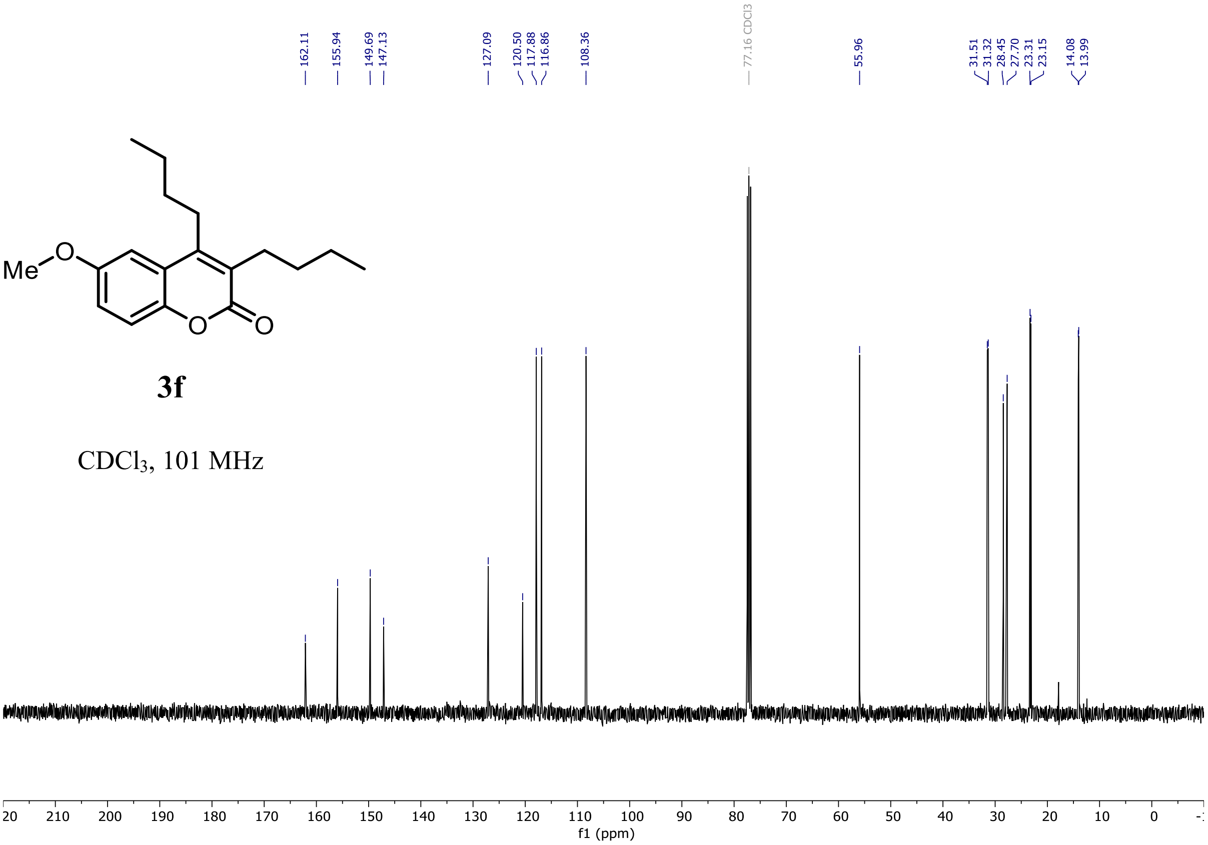

3f

$CDCl_3$, 101 MHz

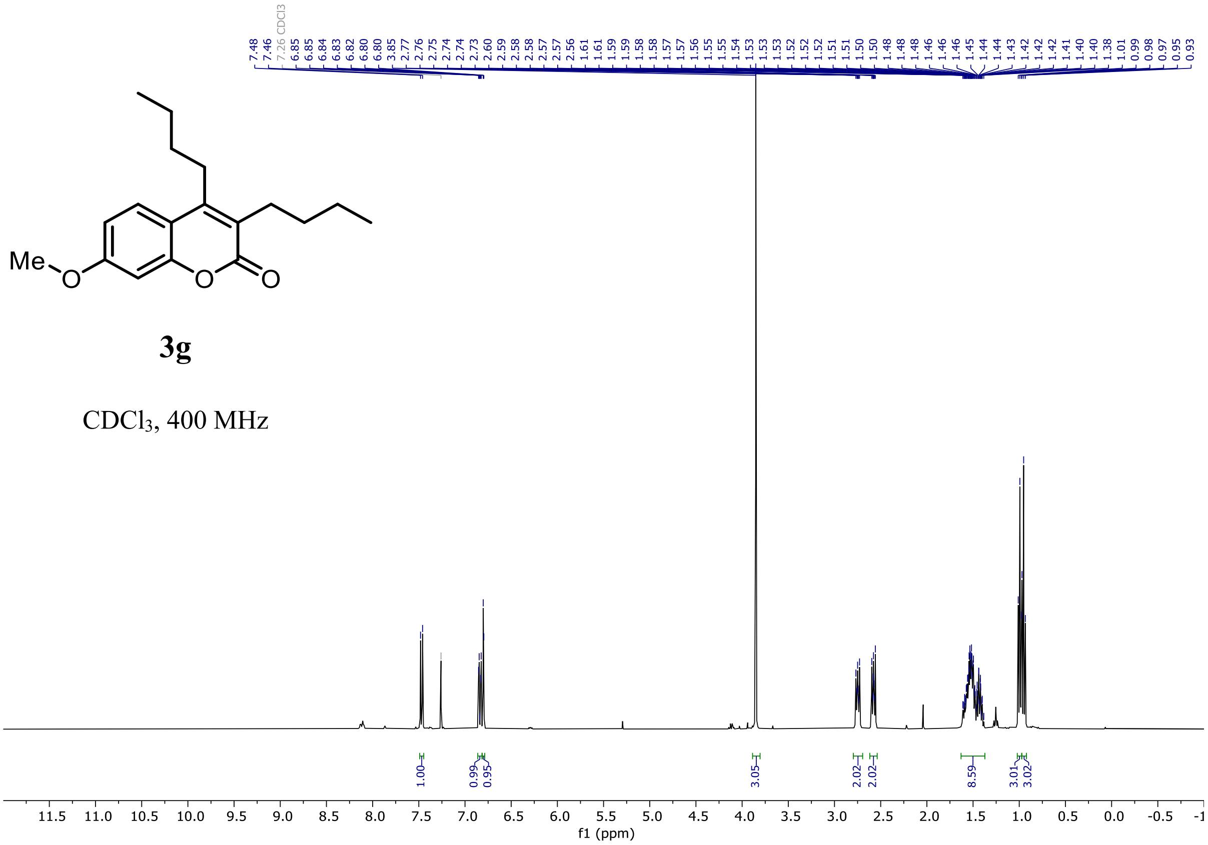

3g

$CDCl_3$, 400 MHz

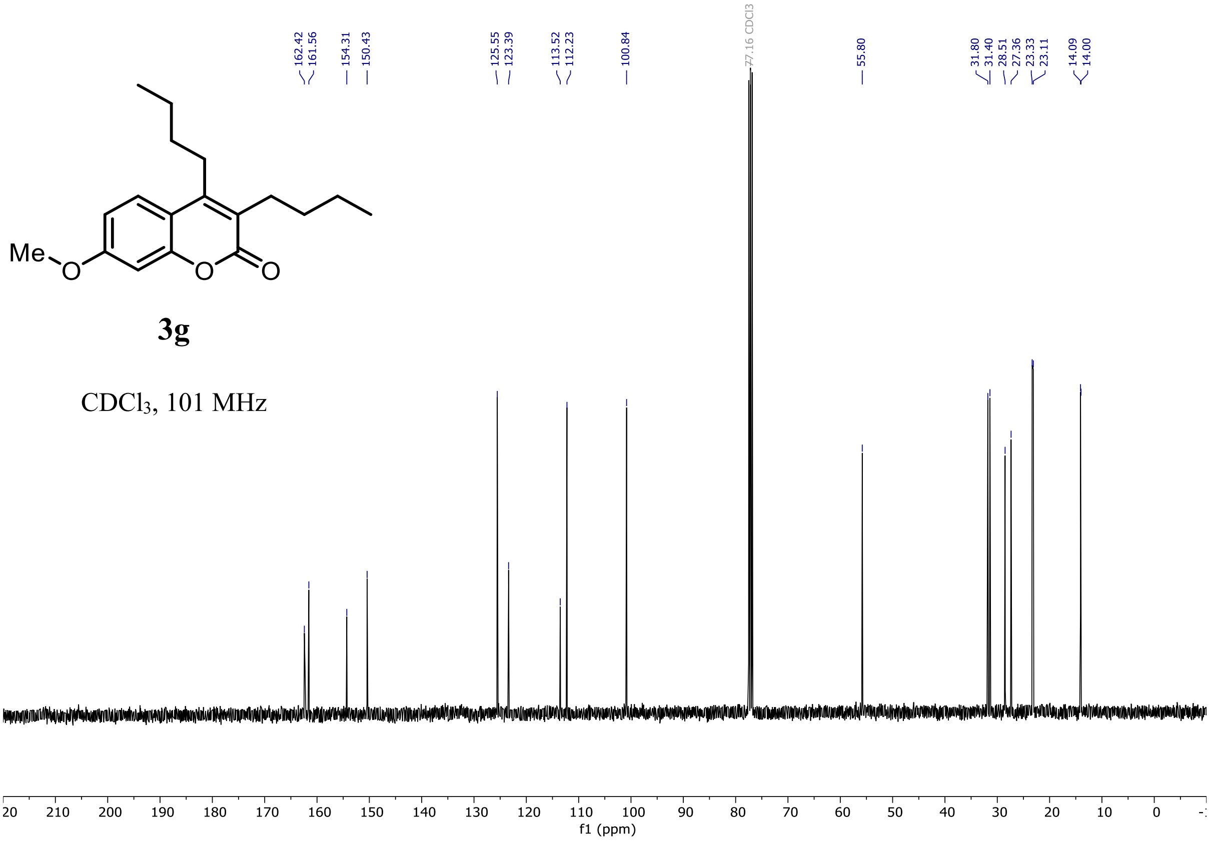

3g

$CDCl_3$, 101 MHz

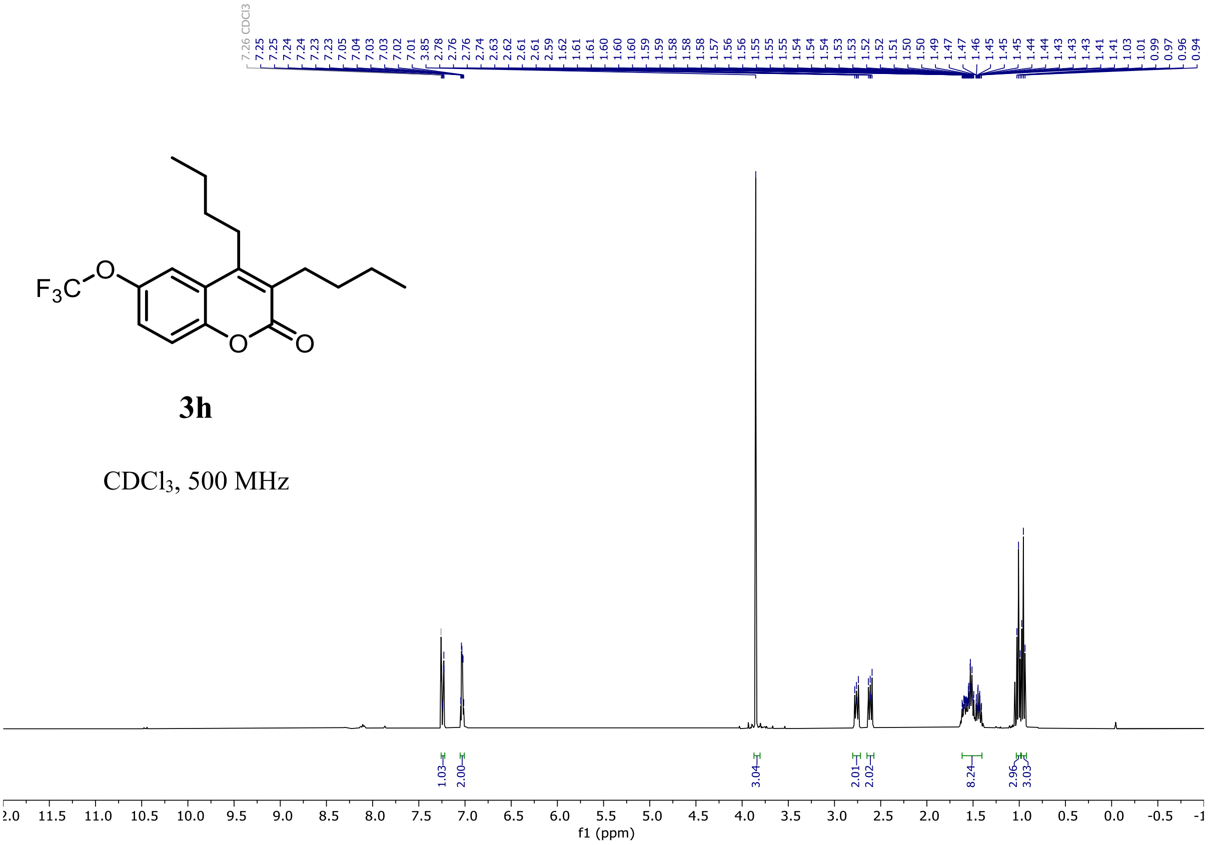

3h

$CDCl_3$, 500 MHz

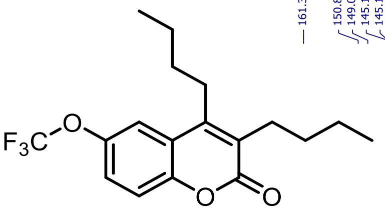

3h

$CDCl_3$, 125 MHz

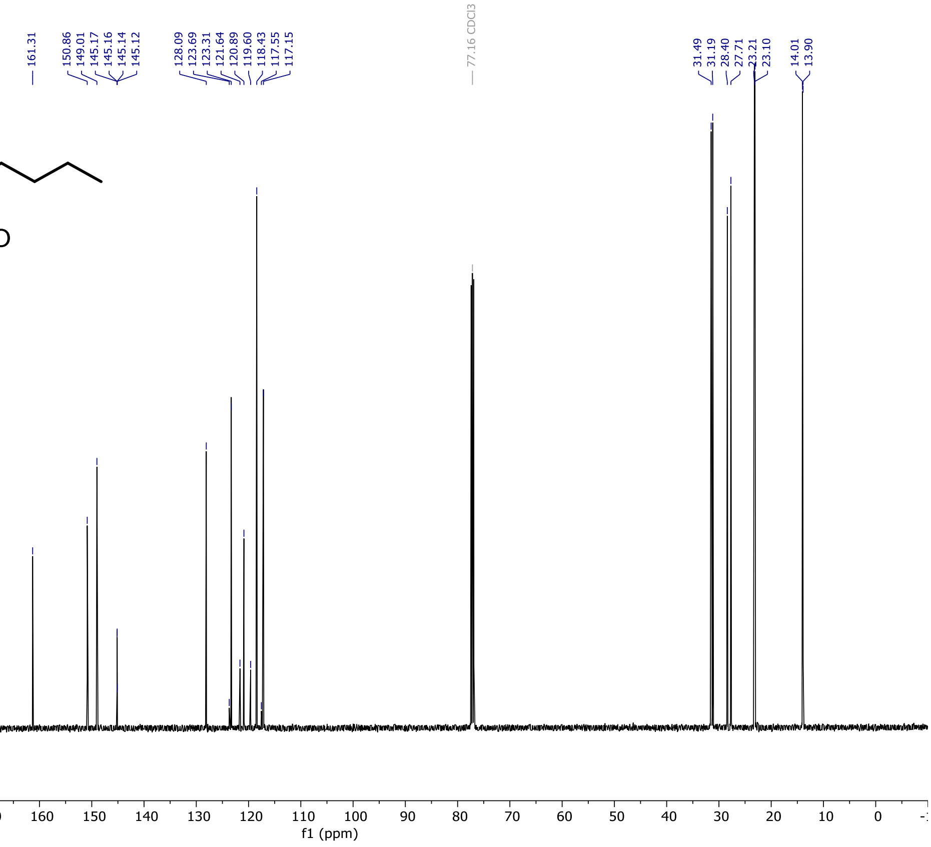

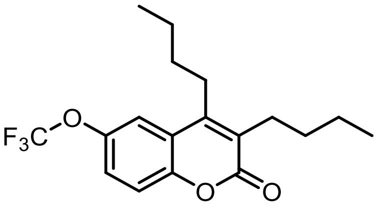

3h

$CDCl_3$, 376 MHz

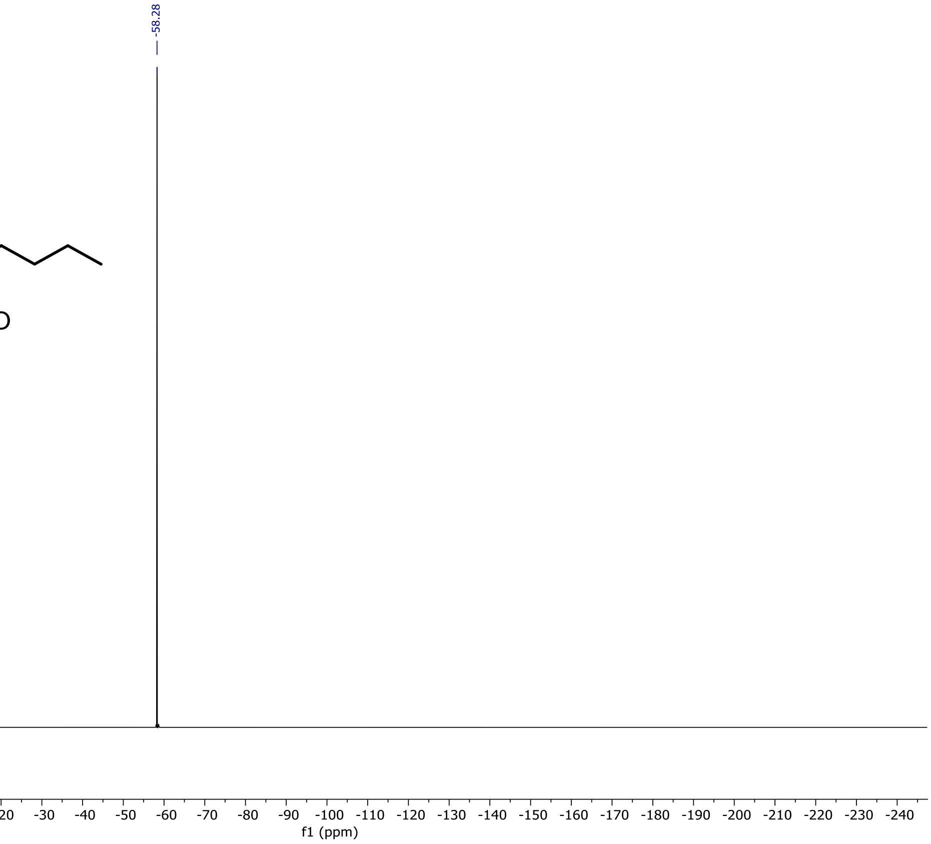

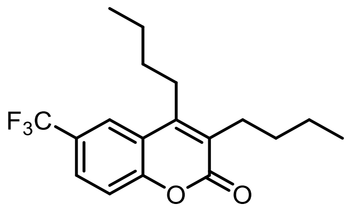

3i

$CDCl_3$, 500 MHz

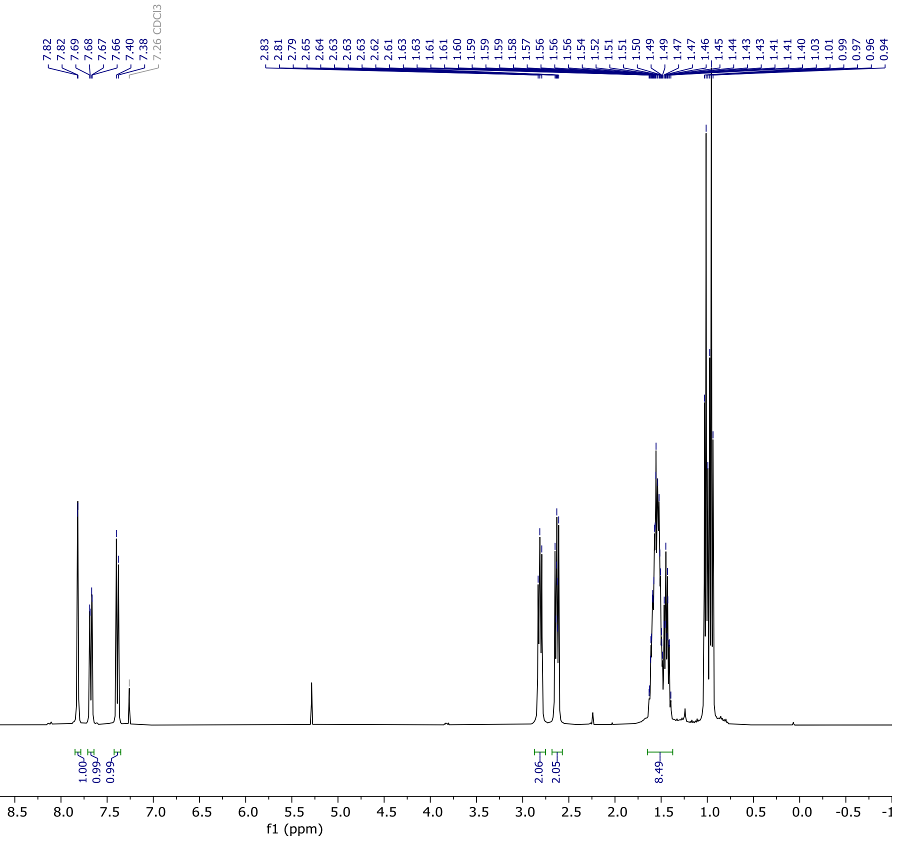

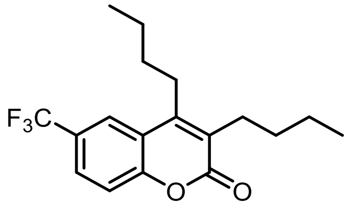

3i

$CDCl_3$, 125 MHz

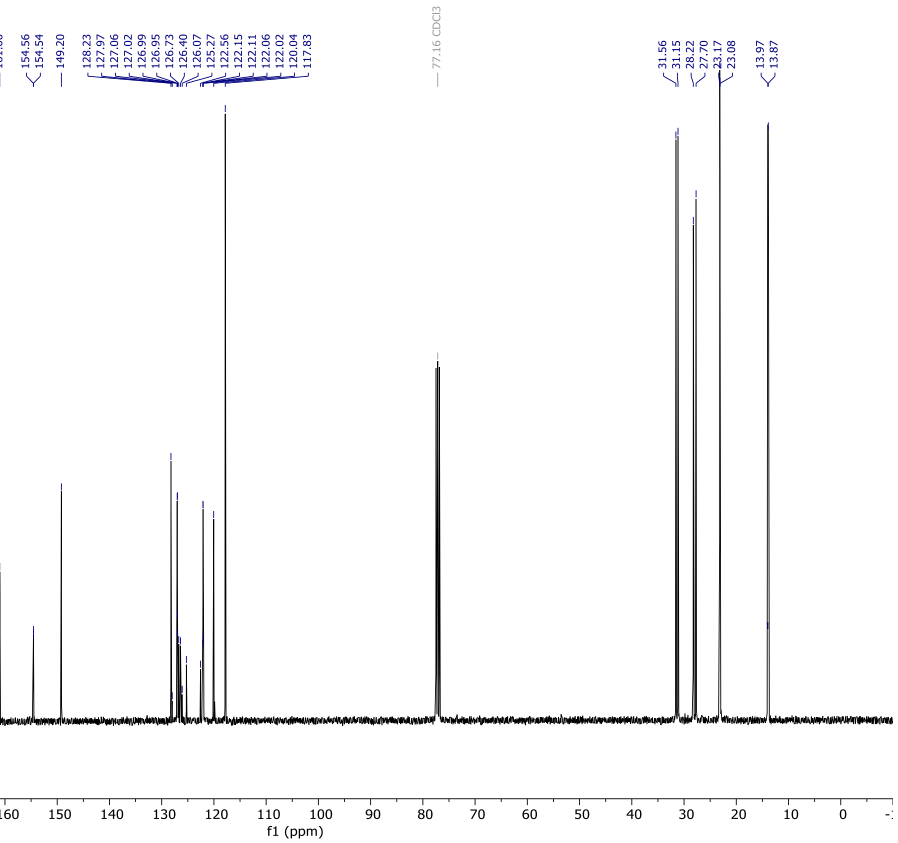

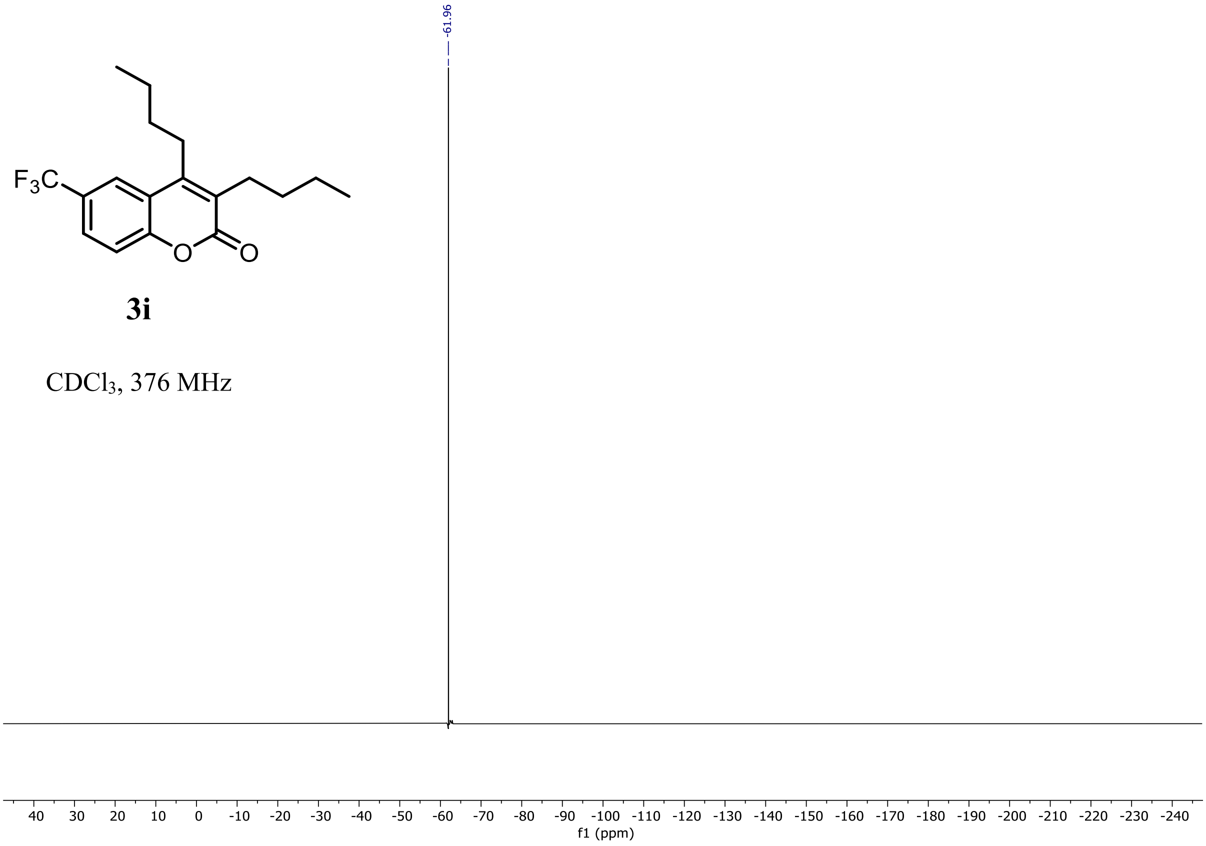

-61.96
F_3C
O
O
3i
CDCl3, 376 MHz
f1 (ppm)

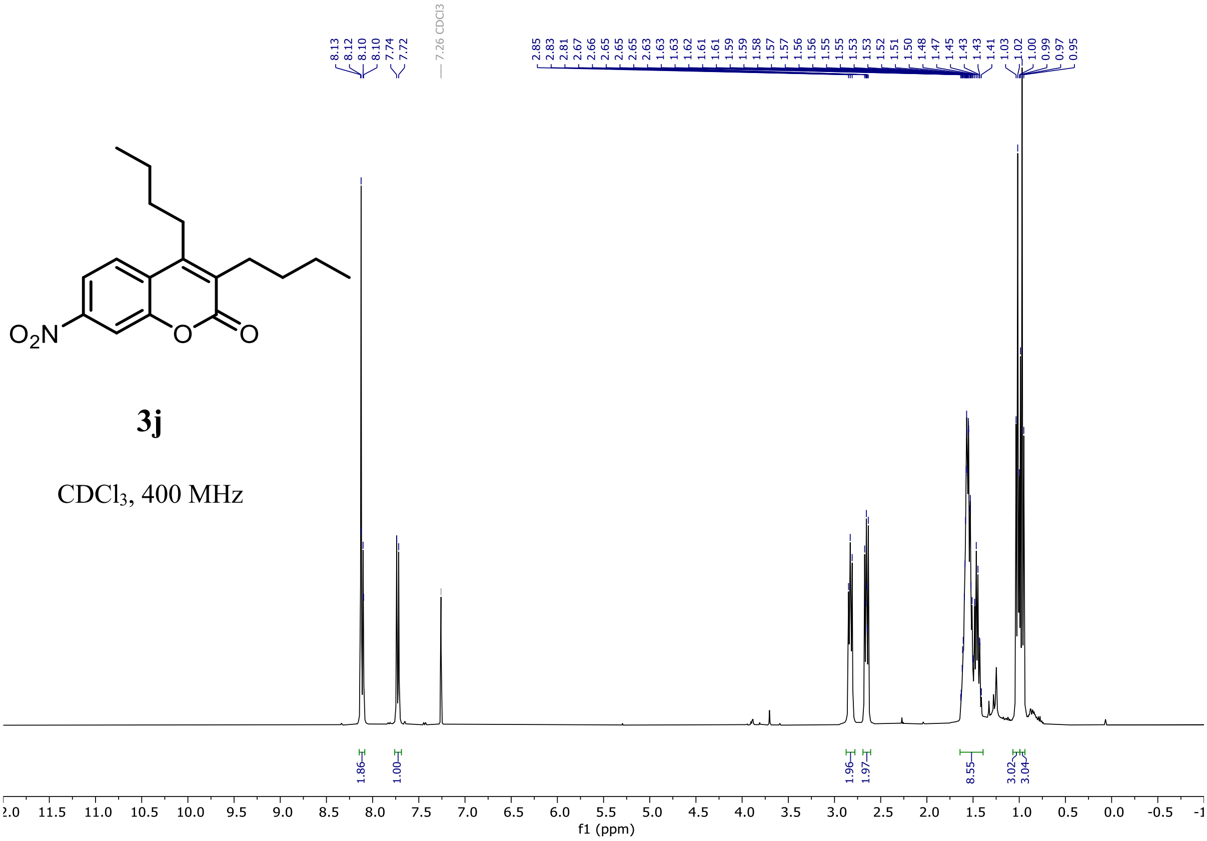

7.26 CDCl3
O_2N
O
O
3j
CDCl3, 400 MHz
1.86
1.00
1.96
1.97
8.55
3.02
3.04
f1 (ppm)

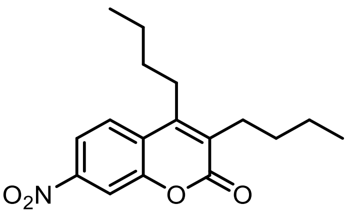

3j

$CDCl_3$, 101 MHz

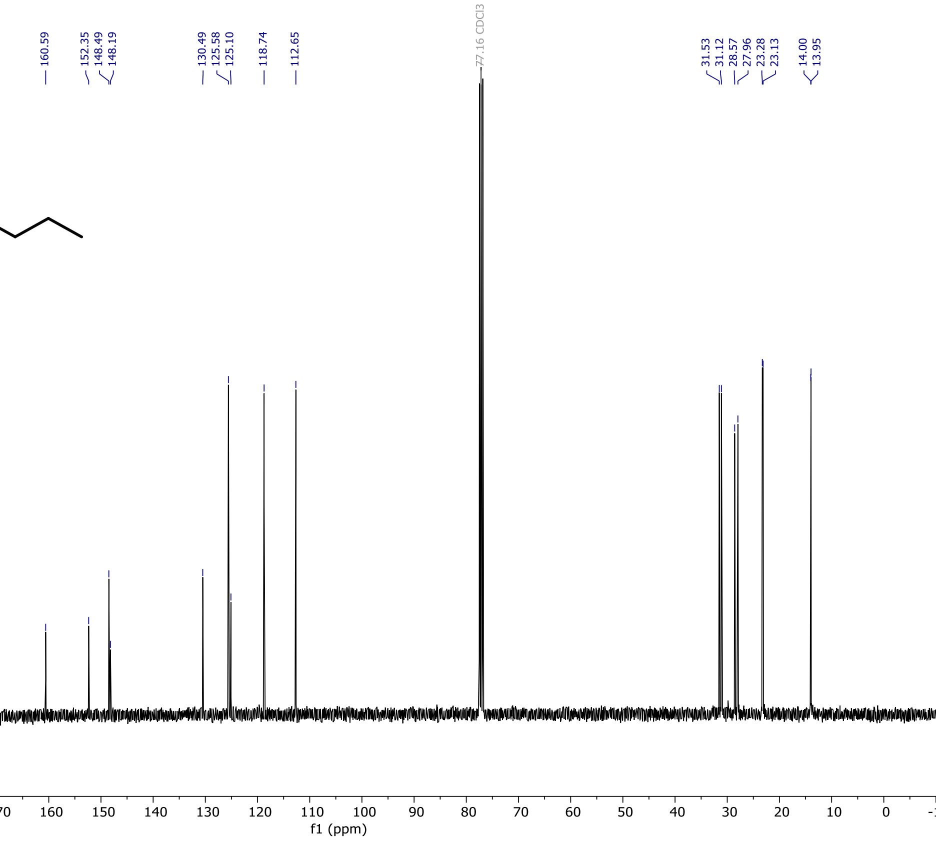

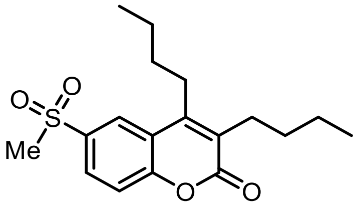

3k

$CDCl_3$, 400 MHz

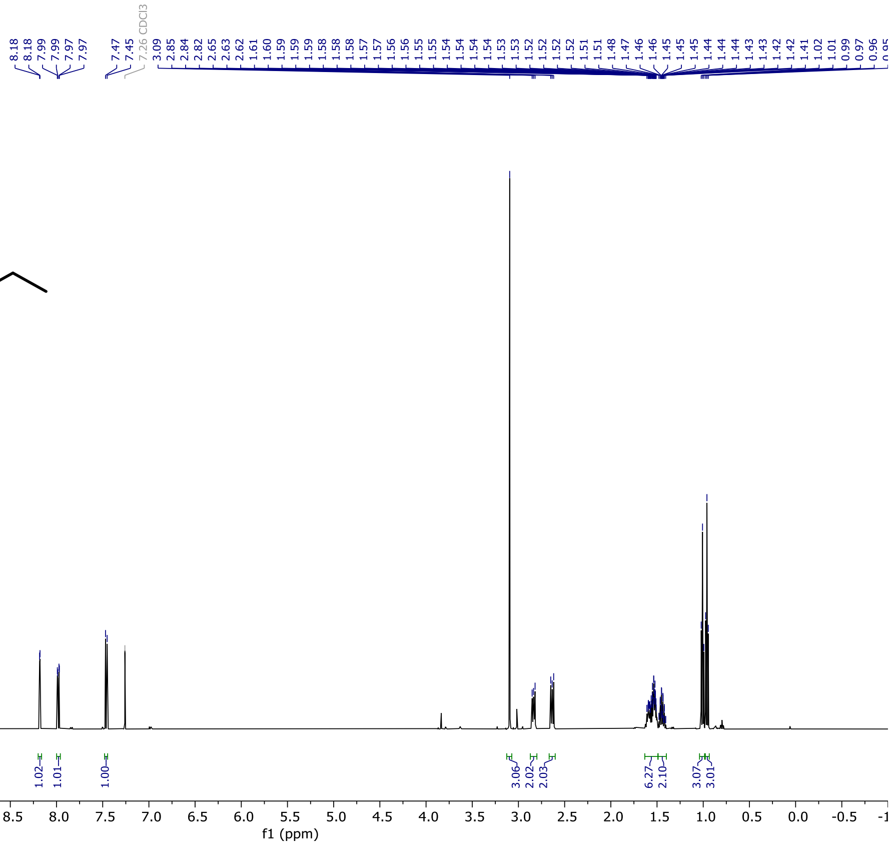

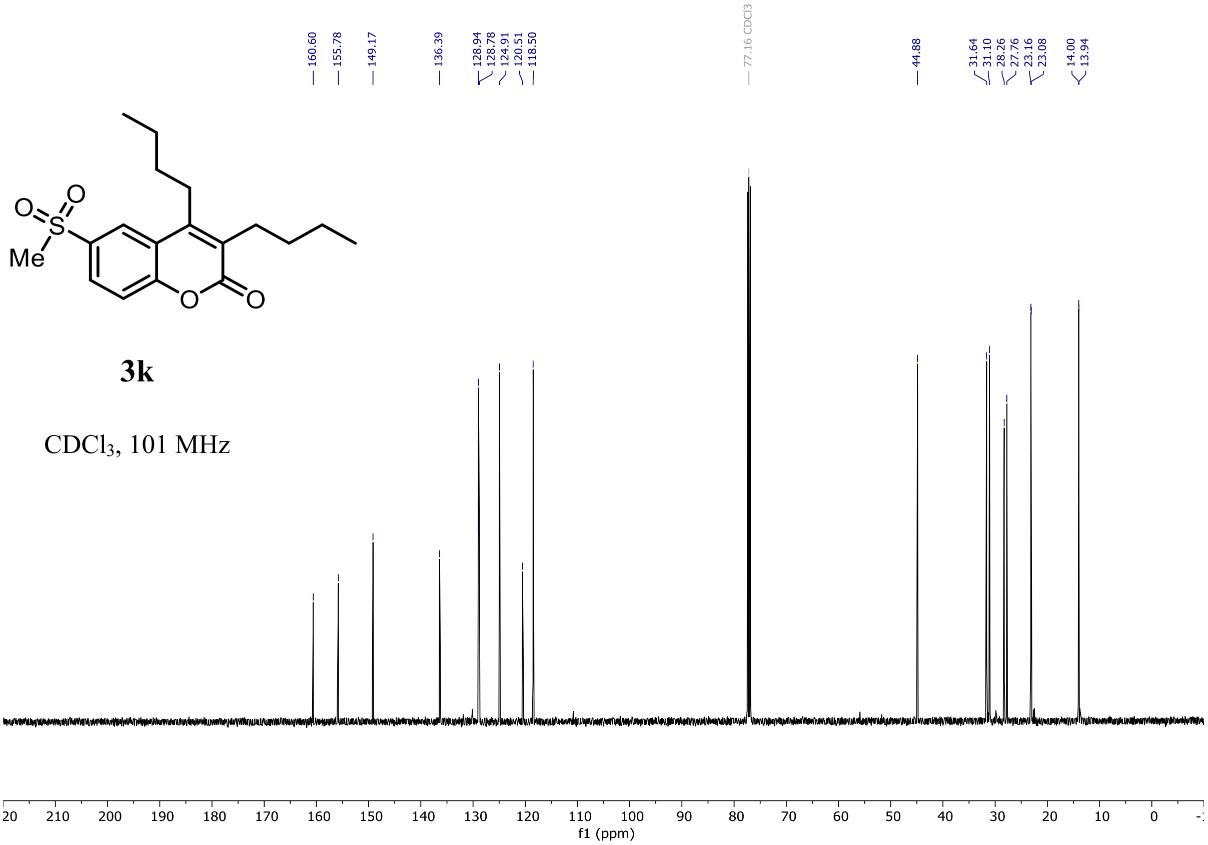

3k

CDCl3, 101 MHz

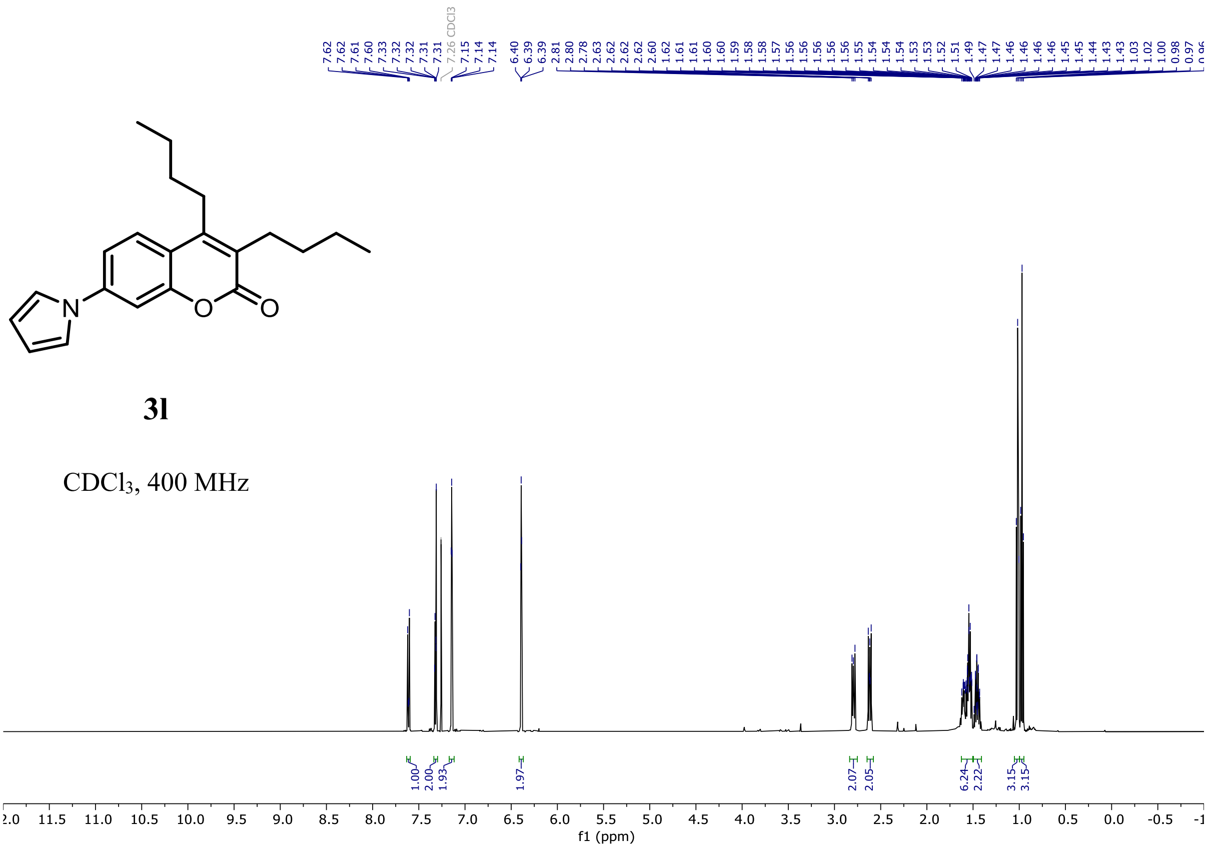

3l

CDCl3, 400 MHz

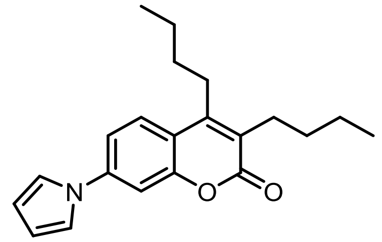

3l

CDCl$_3$, 101 MHz

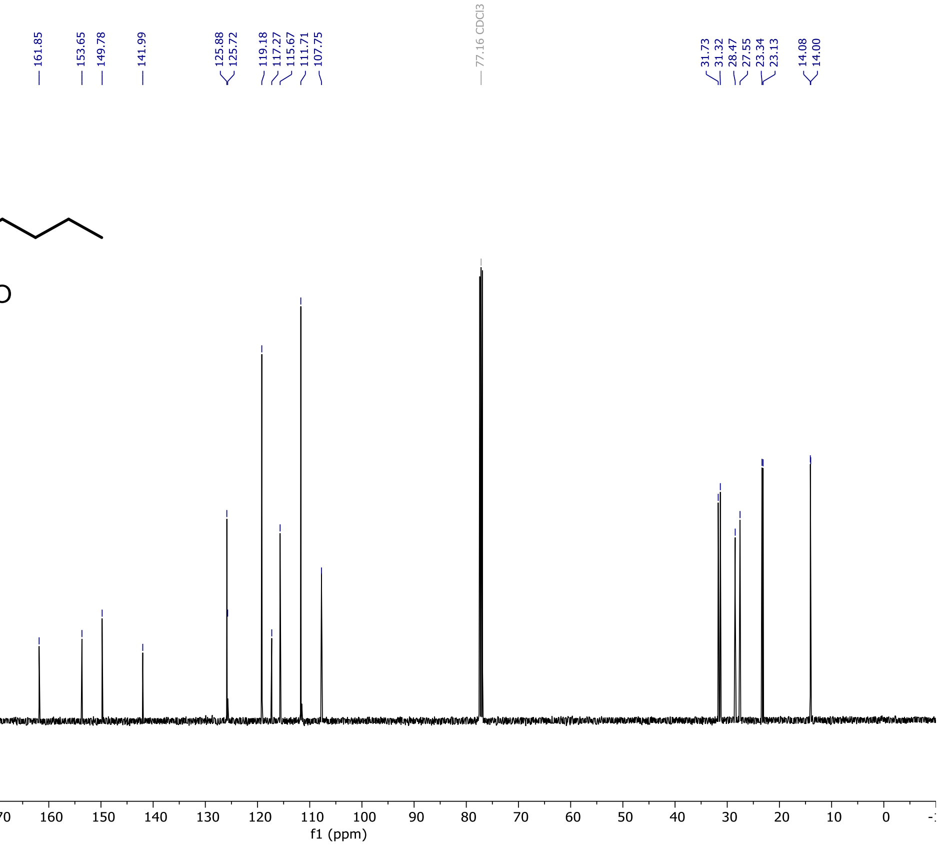

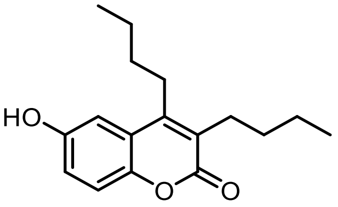

3m

CDCl$_3$, 400 MHz

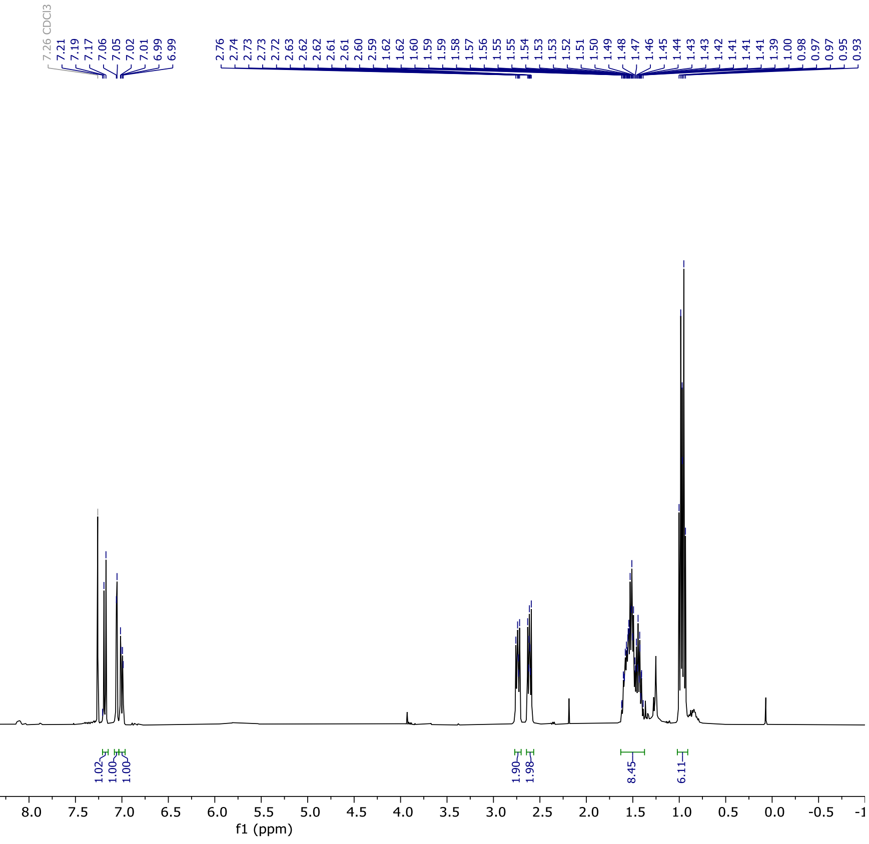

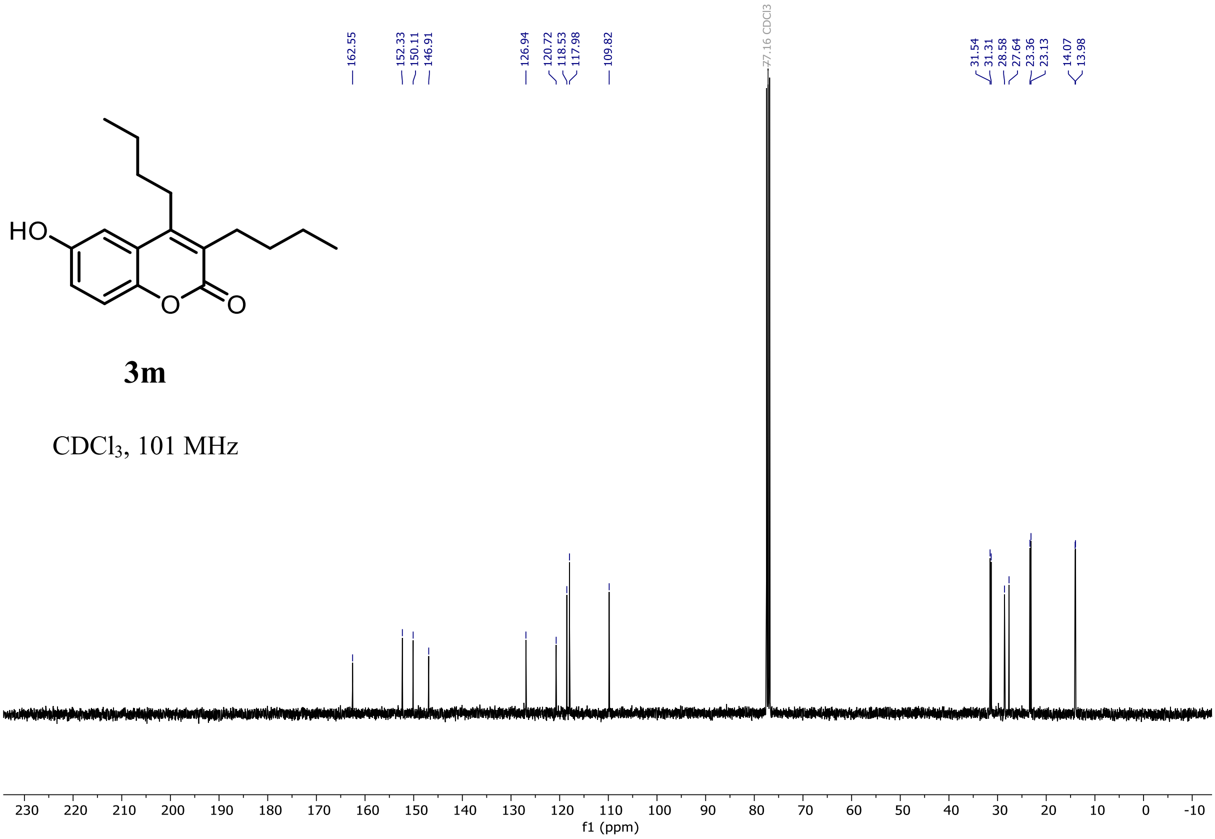
162.55
152.33
150.11
146.91
126.94
120.72
118.53
117.98
109.82
77.16 CDCl3
31.54
31.31
28.58
27.64
23.36
23.13
14.07
13.98
HO
O
O
3m
CDCl3, 101 MHz
230 220 210 200 190 180 170 160 150 140 130 120 110 100 90 80 70 60 50 40 30 20 10 0 -10
f1 (ppm)

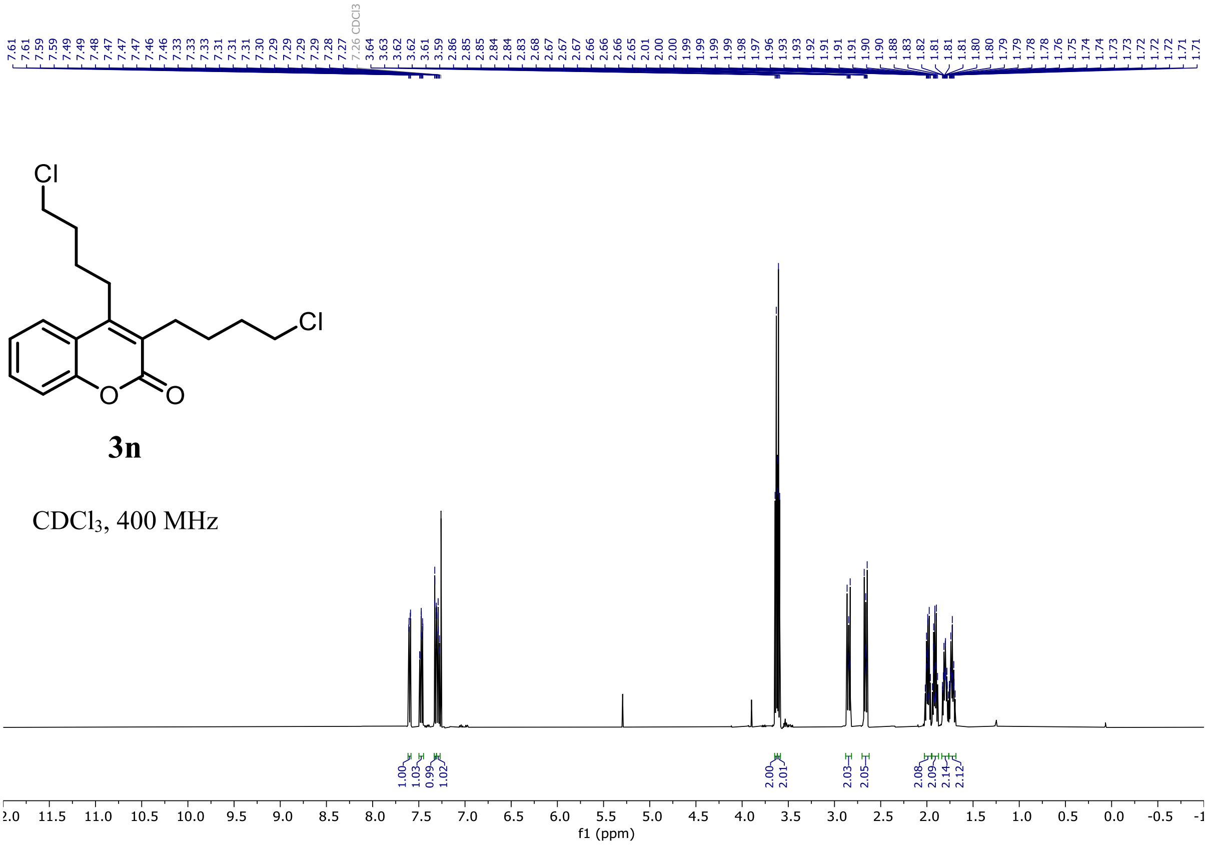
7.61 7.61 7.59 7.59 7.49 7.48 7.47 7.47 7.47 7.46 7.33 7.33 7.33 7.31 7.31 7.30 7.29 7.29 7.29 7.29 7.28 7.27 7.26 CDCl3 3.64 3.63 3.62 3.61 3.59 2.86 2.85 2.85 2.84 2.83 2.68 2.67 2.67 2.66 2.66 2.65 2.01 2.00 2.00 1.99 1.99 1.99 1.98 1.98 1.97 1.96 1.93 1.93 1.93 1.92 1.91 1.91 1.91 1.90 1.90 1.88 1.83 1.82 1.81 1.81 1.81 1.80 1.80 1.79 1.78 1.78 1.76 1.75 1.74 1.74 1.73 1.73 1.72 1.72 1.71 1.71
Cl
Cl
O
O
3n
CDCl3, 400 MHz
1.00
1.03
0.99
1.02
2.00
2.01
2.03
2.05
2.08
2.09
2.14
2.12
11.5 11.0 10.5 10.0 9.5 9.0 8.5 8.0 7.5 7.0 6.5 6.0 5.5 5.0 4.5 4.0 3.5 3.0 2.5 2.0 1.5 1.0 0.5 0.0 -0.5
f1 (ppm)

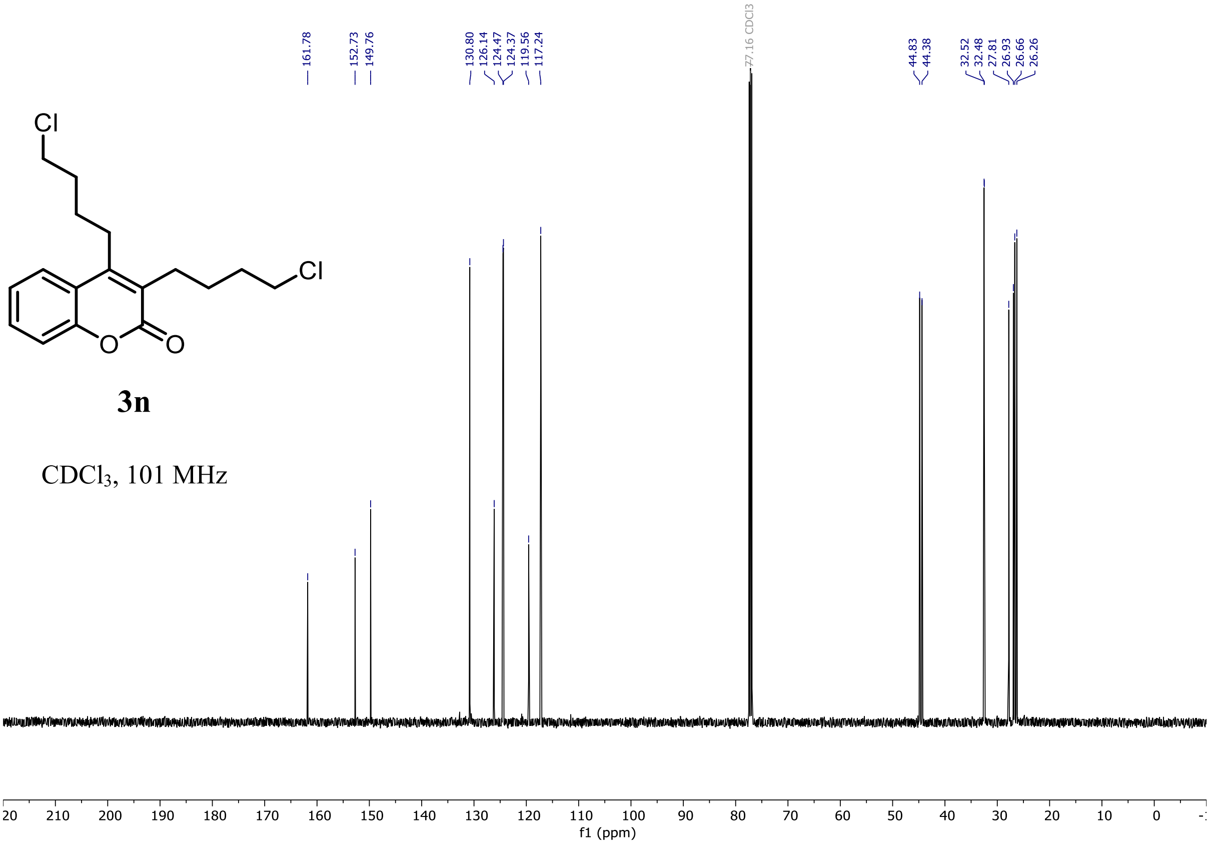

3n

$CDCl_3$, 101 MHz

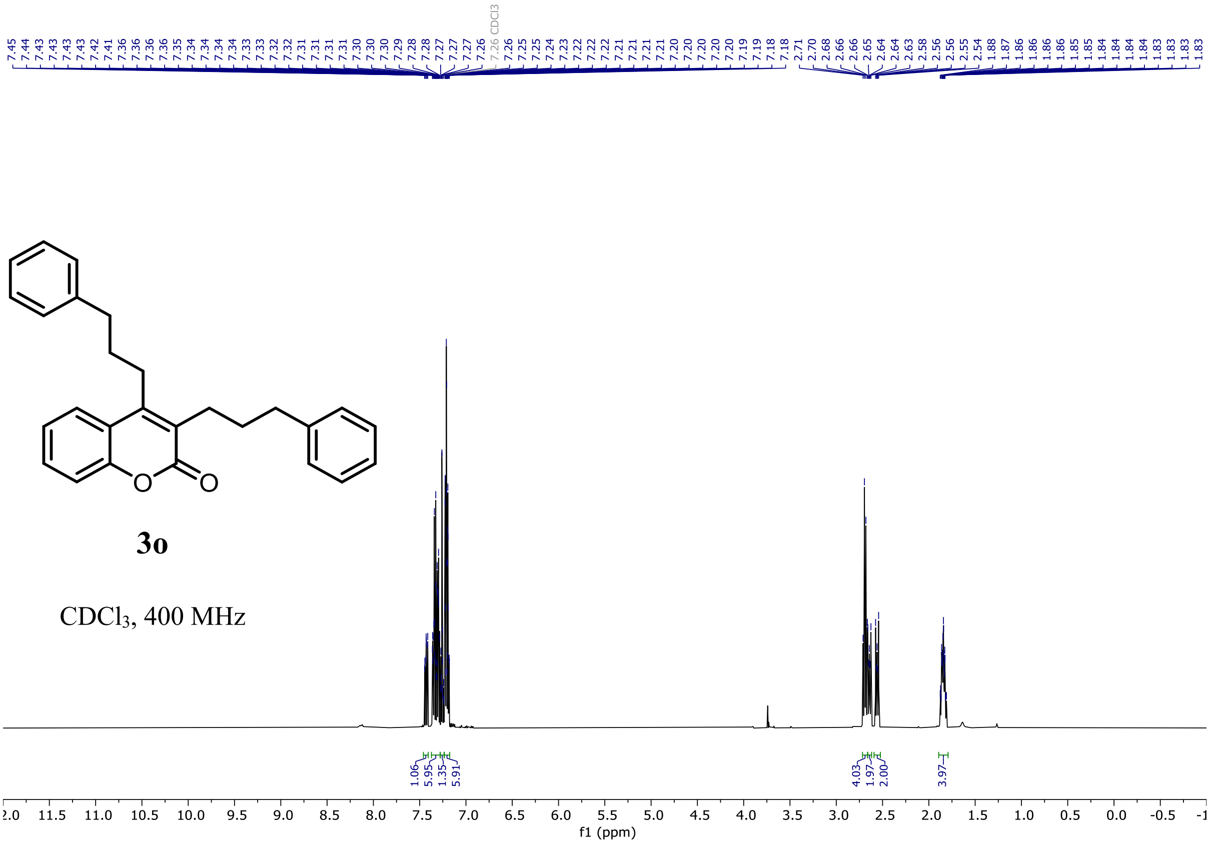

3o

$CDCl_3$, 400 MHz

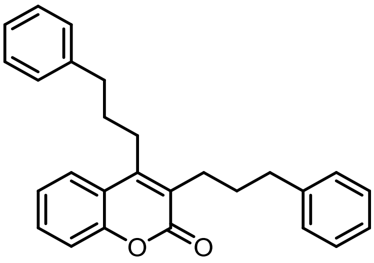

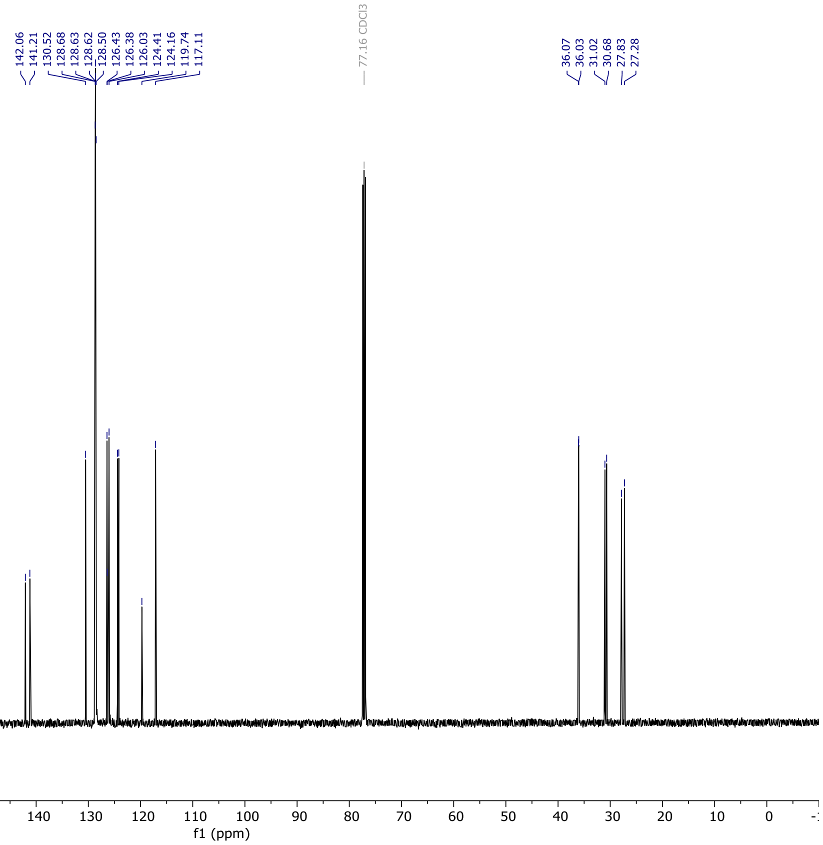

3o

CDCl$_3$, 101 MHz

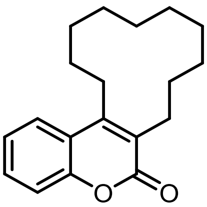

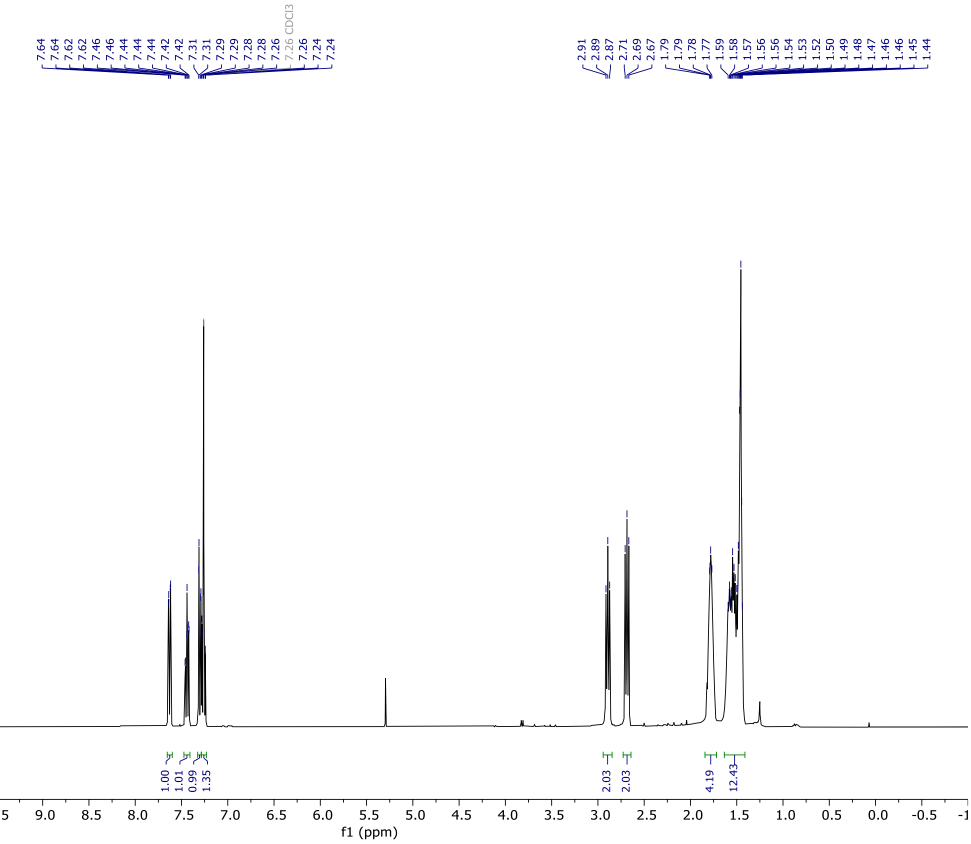

3p

CDCl$_3$, 400 MHz

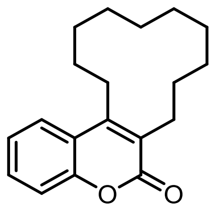

3p

$CDCl_3$, 101 MHz

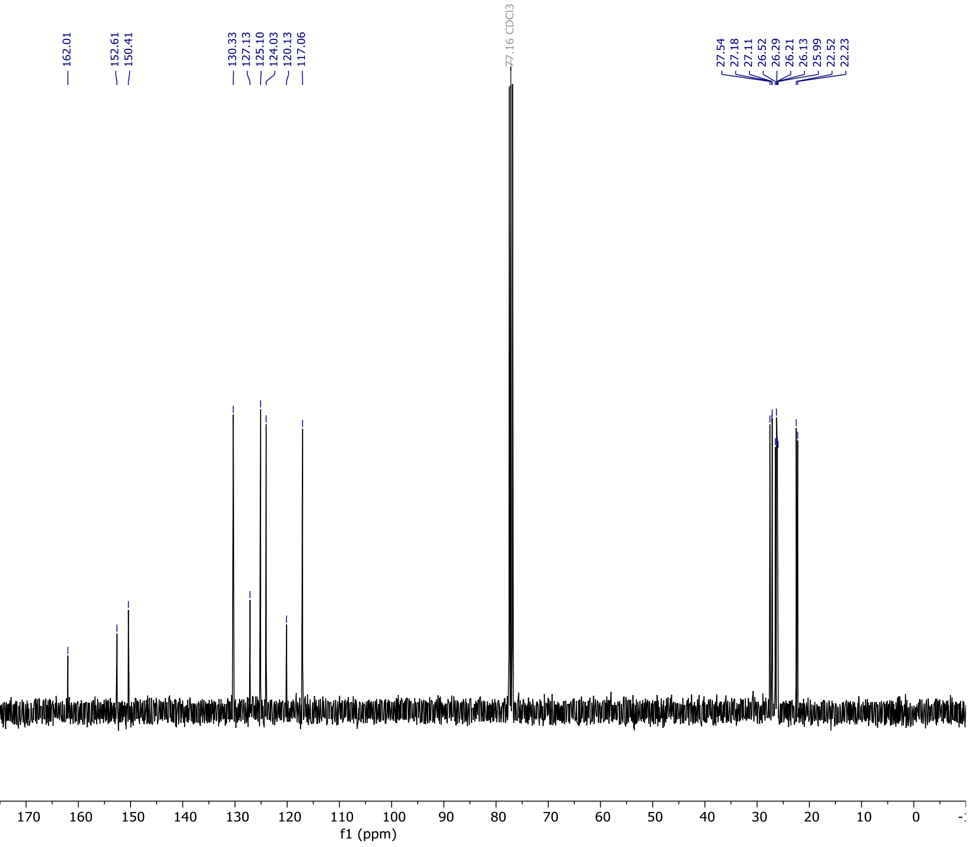

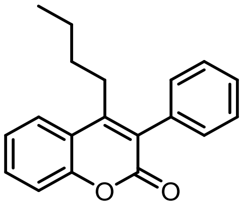

3q[α]

$CDCl_3$, 400 MHz

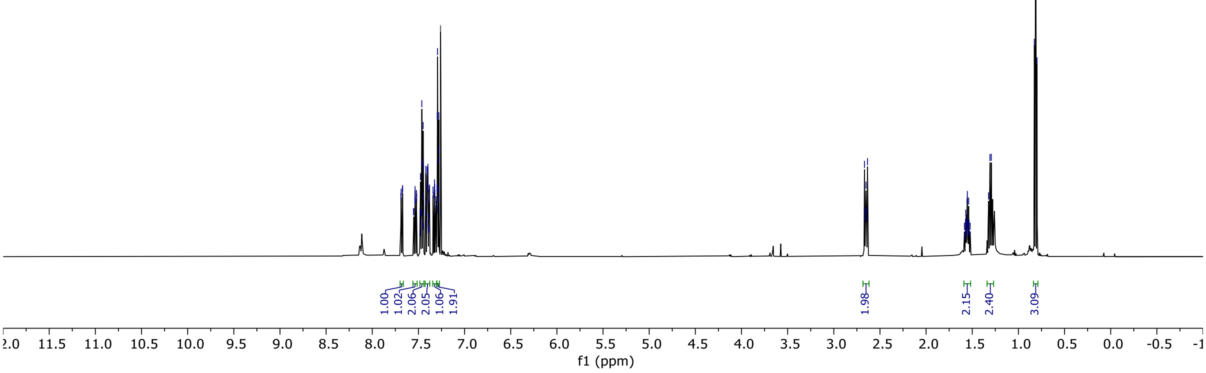

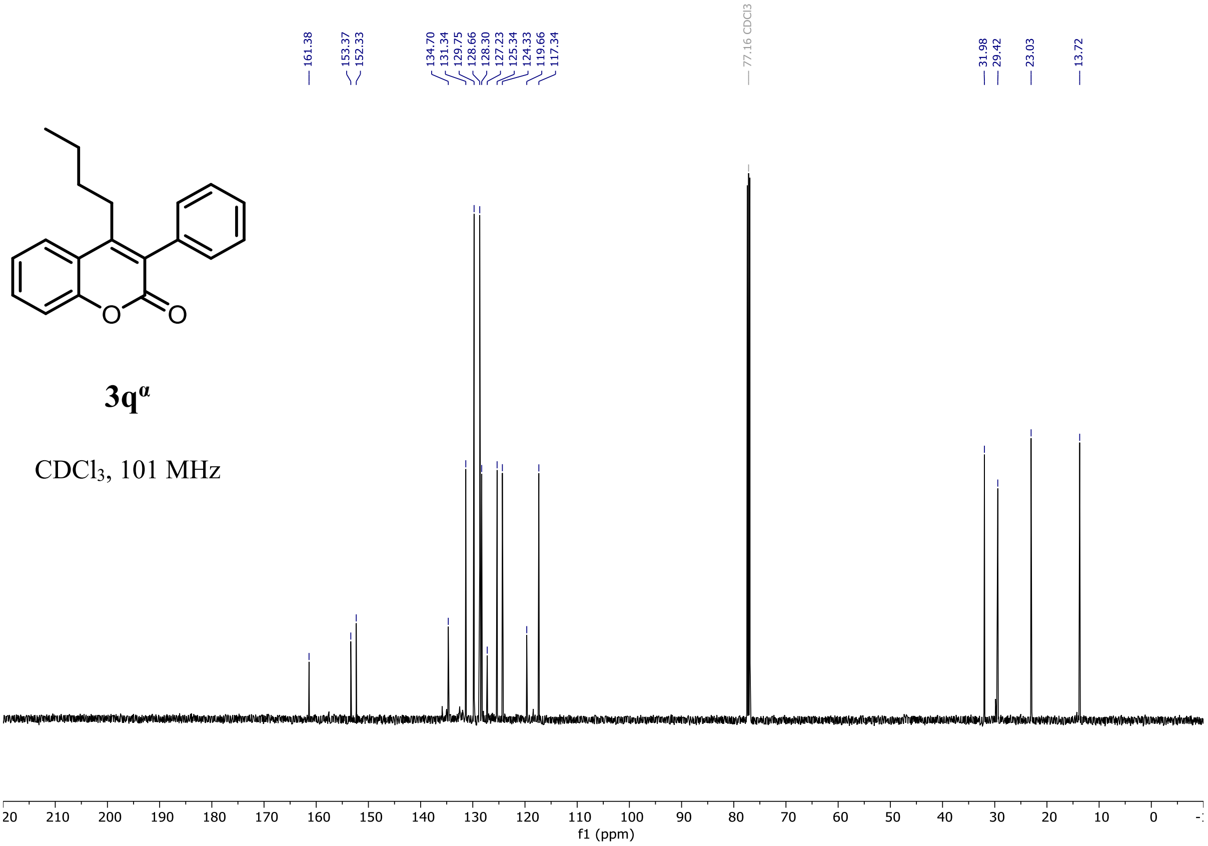

3q$^{\alpha}$

$CDCl_3$, 101 MHz

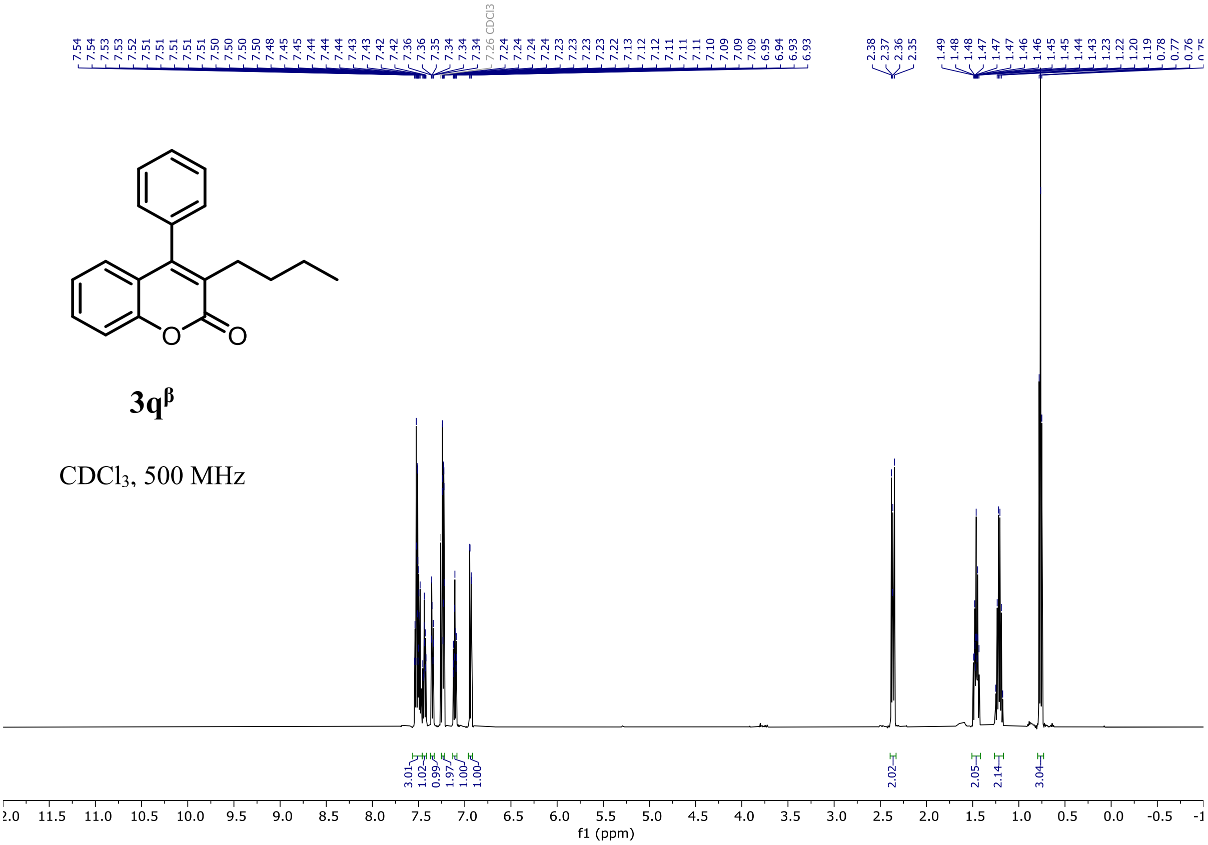

3q$^{\beta}$

$CDCl_3$, 500 MHz

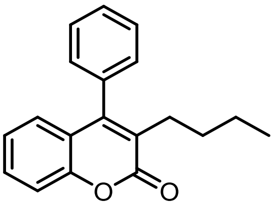

3q[β]

$CDCl_3$, 125 MHz

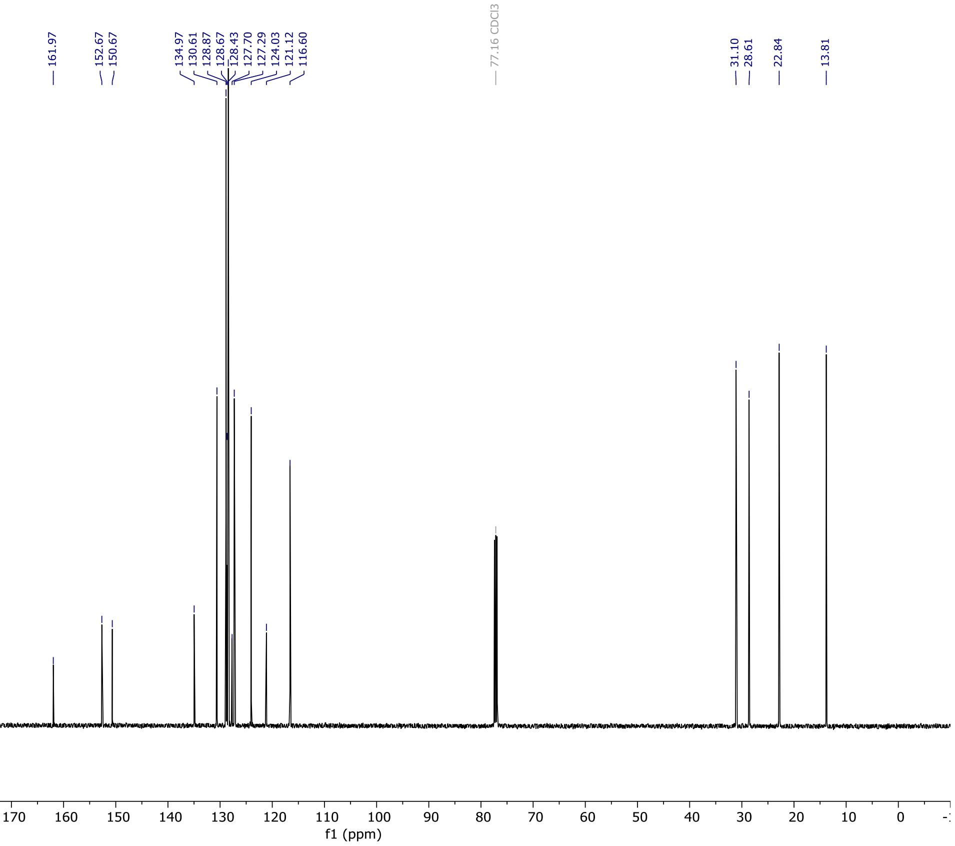

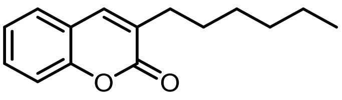

3r[α]

$CDCl_3$, 400 MHz

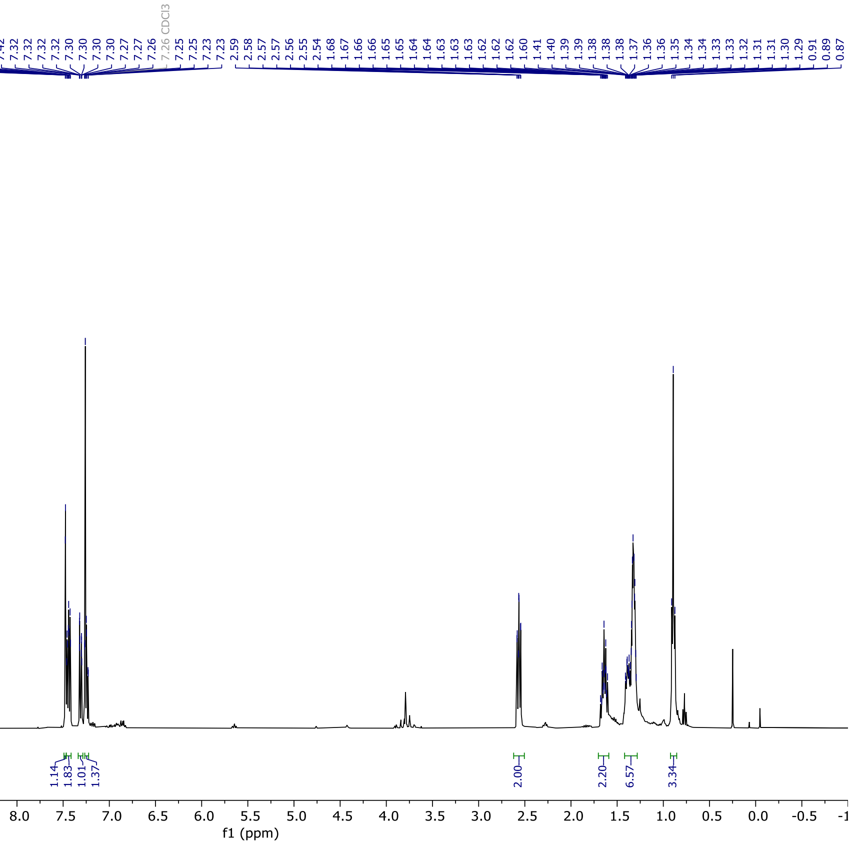

3r$^{\alpha}$

$CDCl_3$, 101 MHz

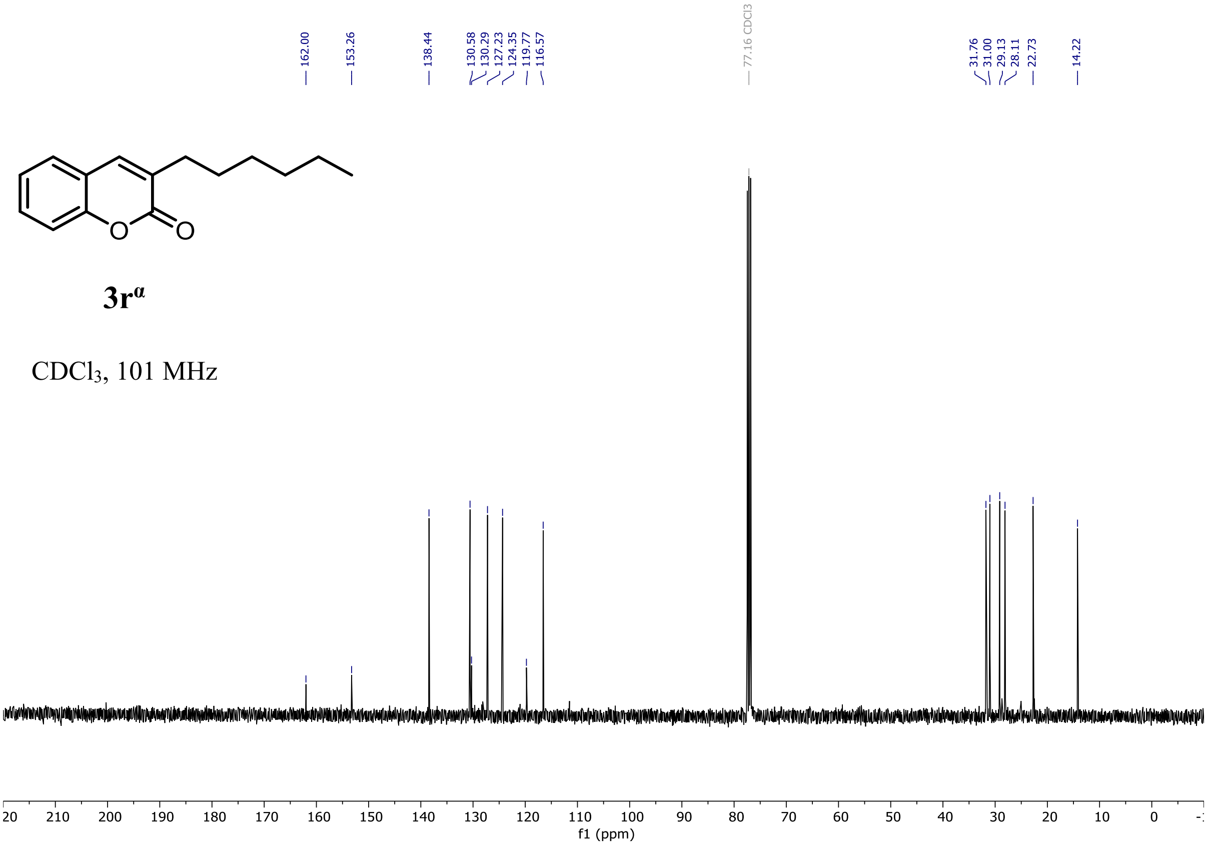

3r$^{\beta}$

$CDCl_3$, 400 MHz

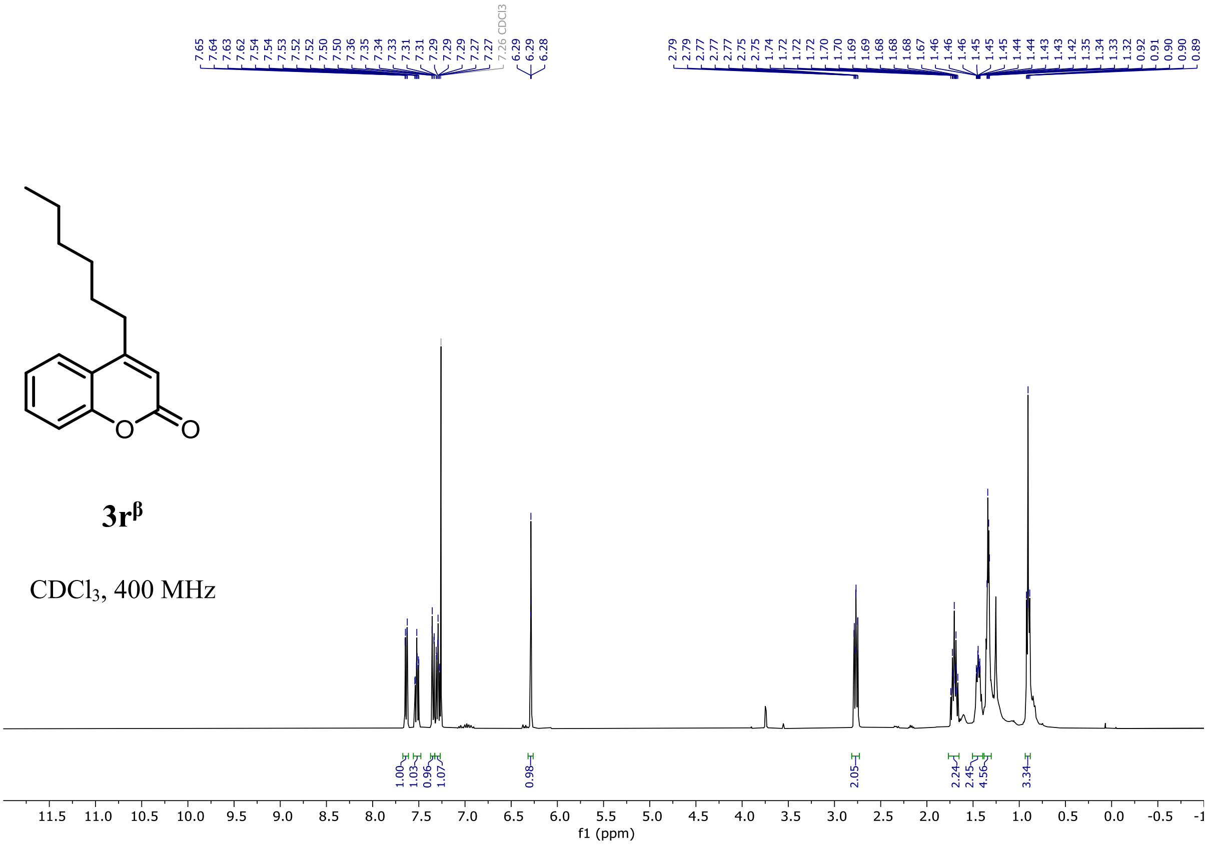

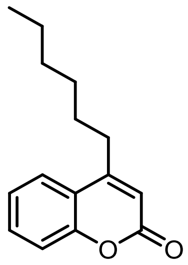

3r$^{\beta}$

CDCl$_3$, 101 MHz

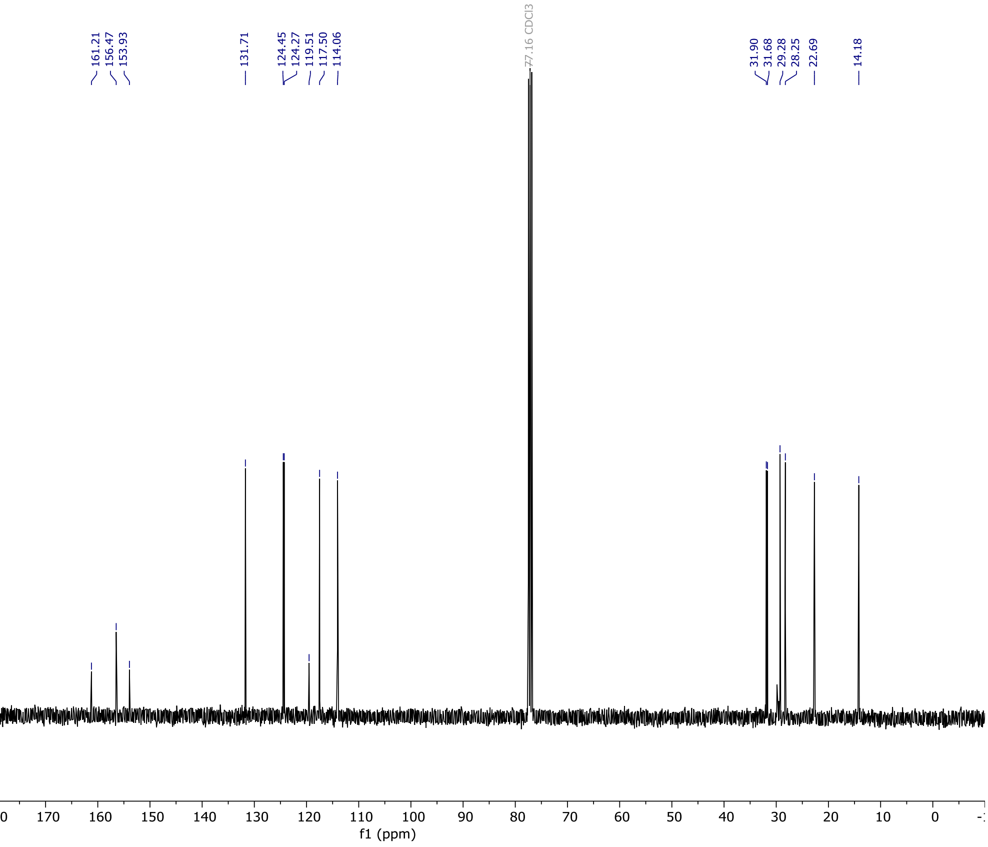

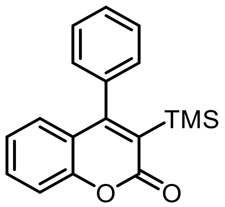

3s

CDCl$_3$, 400 MHz

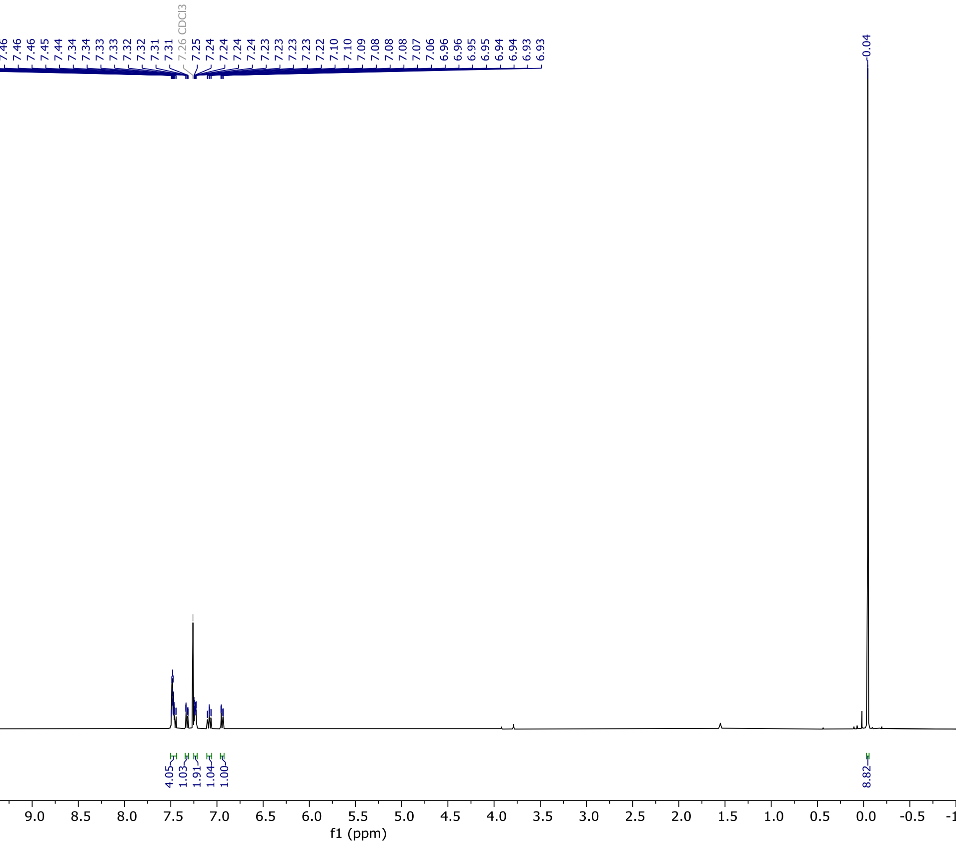

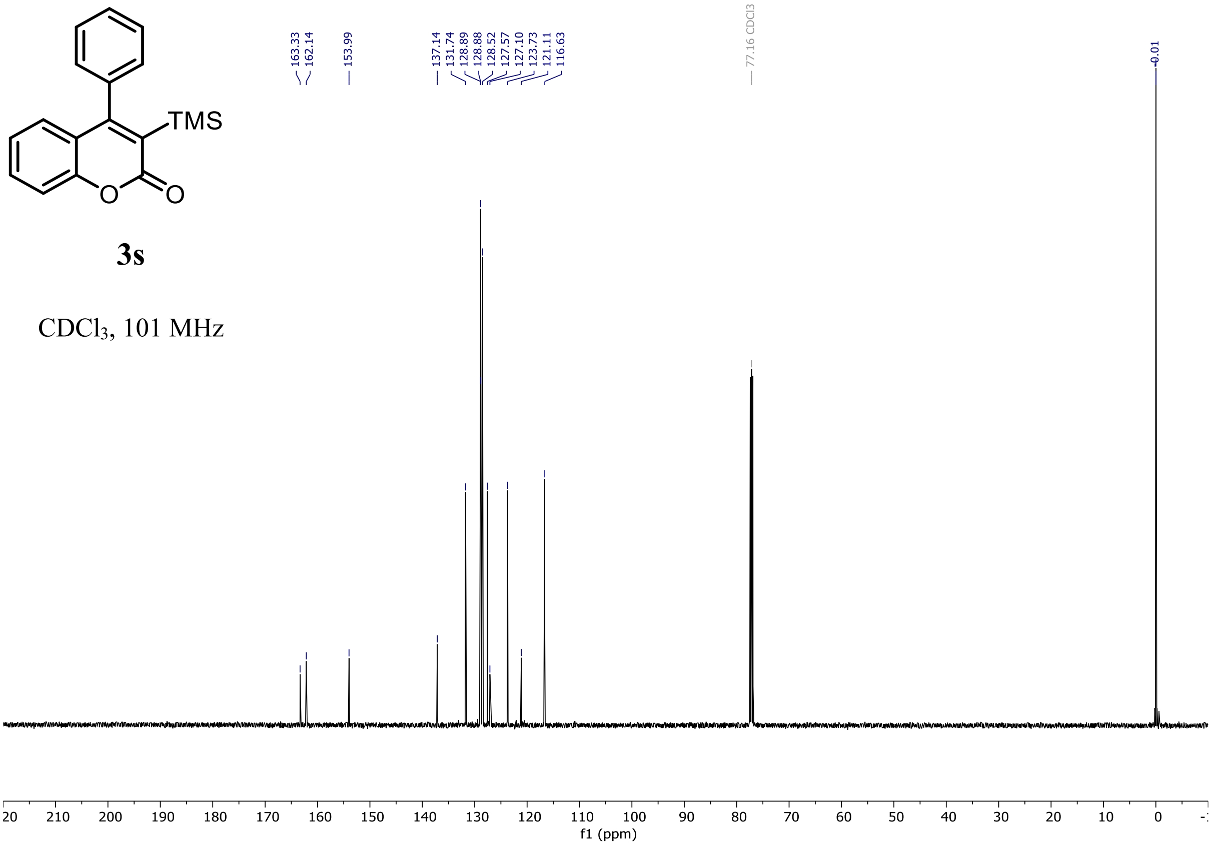

TMS
O
O
3s
CDCl3, 101 MHz
163.33
162.14
153.99
137.14
131.74
128.89
128.88
128.52
127.57
127.10
123.73
121.11
116.63
77.16 CDCl3
0.01
20 210 200 190 180 170 160 150 140 130 120 110 100 90 80 70 60 50 40 30 20 10 0
f1 (ppm)

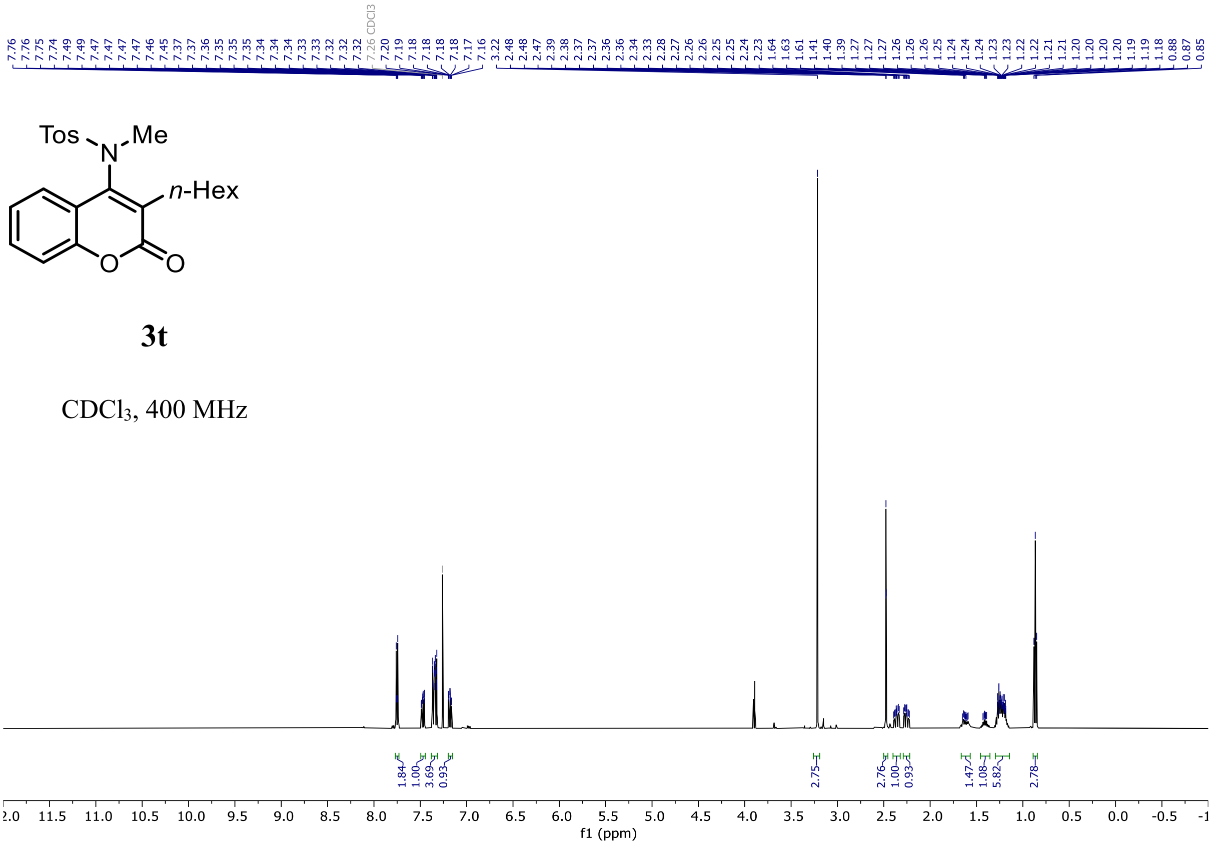

Tos
N
Me
n-Hex
O
O
3t
CDCl3, 400 MHz
7.76 7.76 7.75 7.74 7.49 7.49 7.47 7.47 7.47 7.46 7.45 7.37 7.37 7.36 7.35 7.35 7.34 7.34 7.34 7.33 7.33 7.32 7.32 7.26 CDCl3 7.20 7.19 7.18 7.18 7.18 7.18 7.17 7.16
3.22 2.48 2.48 2.47 2.39 2.38 2.37 2.37 2.36 2.36 2.34 2.33 2.28 2.27 2.26 2.26 2.25 2.24 2.23 1.64 1.63 1.61 1.41 1.40 1.39 1.27 1.27 1.27 1.26 1.26 1.26 1.25 1.24 1.24 1.24 1.23 1.23 1.22 1.22 1.21 1.21 1.20 1.20 1.20 1.20 1.19 1.19 1.18 0.88 0.87 0.85
1.84 1.00 3.69 0.93
2.75
2.76 1.00 0.93
1.47 1.08 5.82
2.78
2.0 11.5 11.0 10.5 10.0 9.5 9.0 8.5 8.0 7.5 7.0 6.5 6.0 5.5 5.0 4.5 4.0 3.5 3.0 2.5 2.0 1.5 1.0 0.5 0.0 -0.5 -1
f1 (ppm)

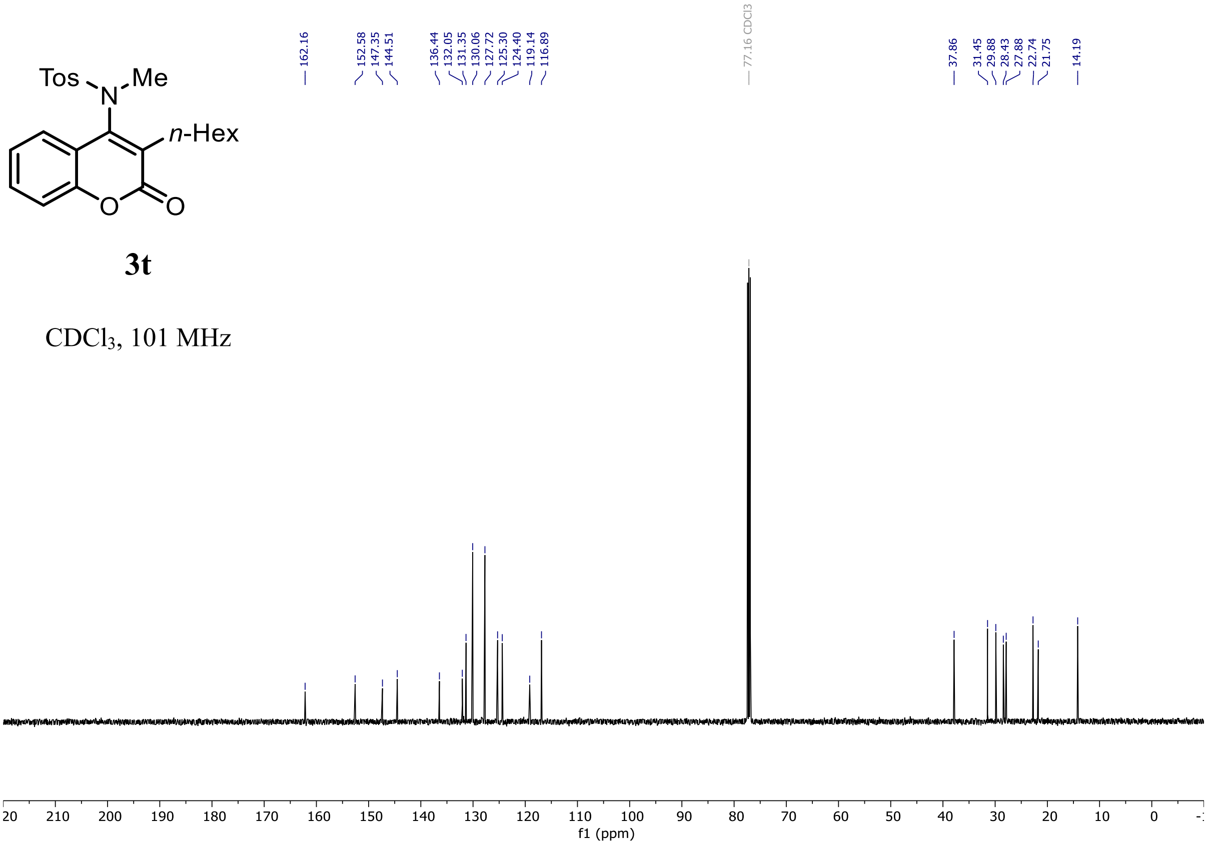
Tos
N
Me
n-Hex
O
O
3t
CDCl3, 101 MHz
162.16
152.58
147.35
144.51
136.44
132.05
131.35
130.06
127.72
125.30
124.40
119.14
116.89
77.16 CDCl3
37.86
31.45
29.88
28.43
27.88
22.74
21.75
14.19
f1 (ppm)

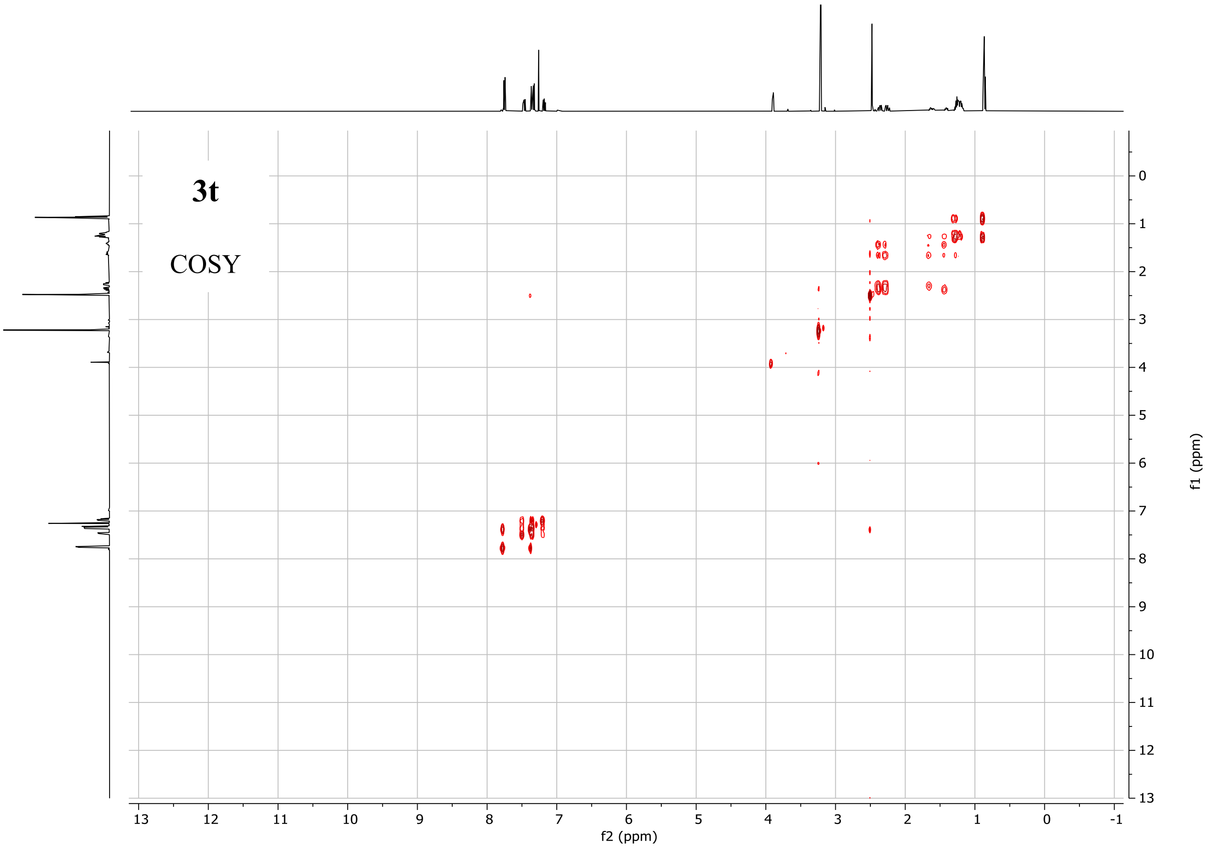
3t
COSY
f2 (ppm)
f1 (ppm)

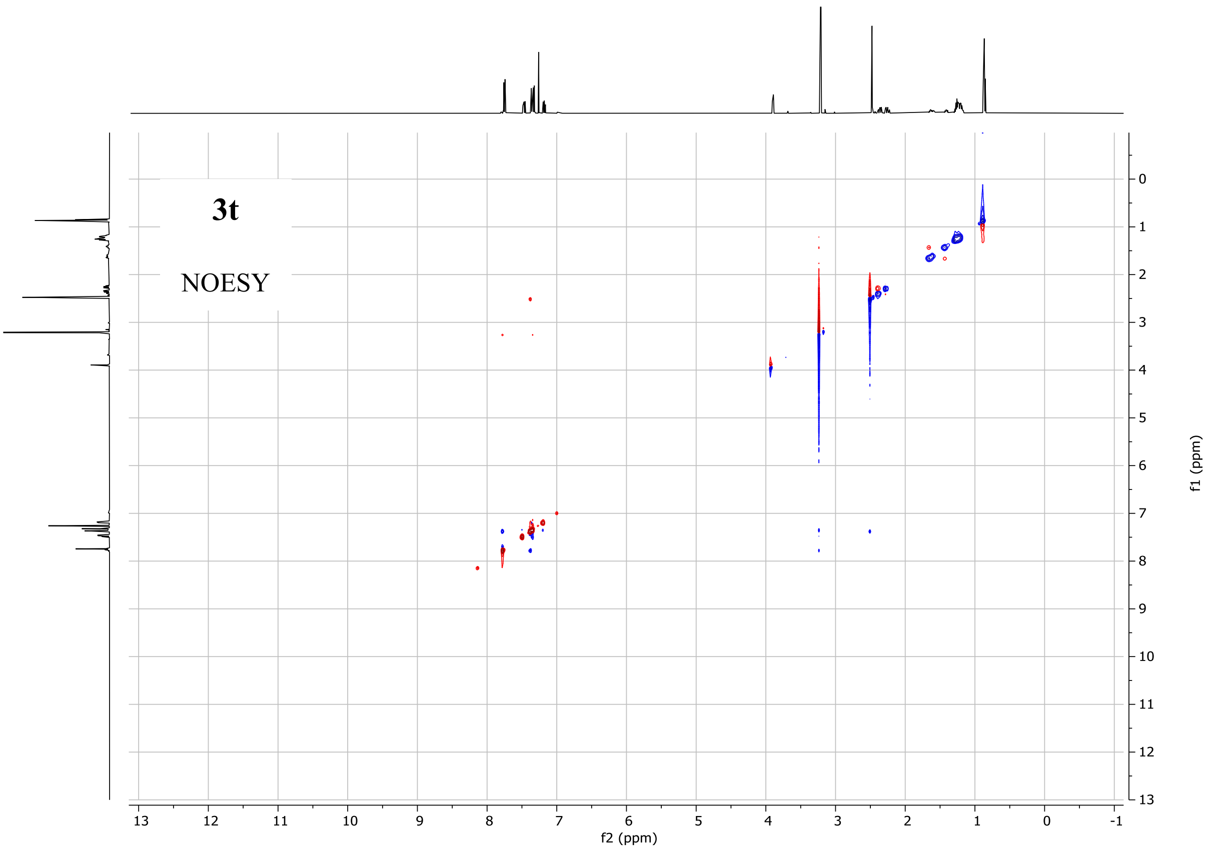
3t
NOESY
f2 (ppm)
f1 (ppm)

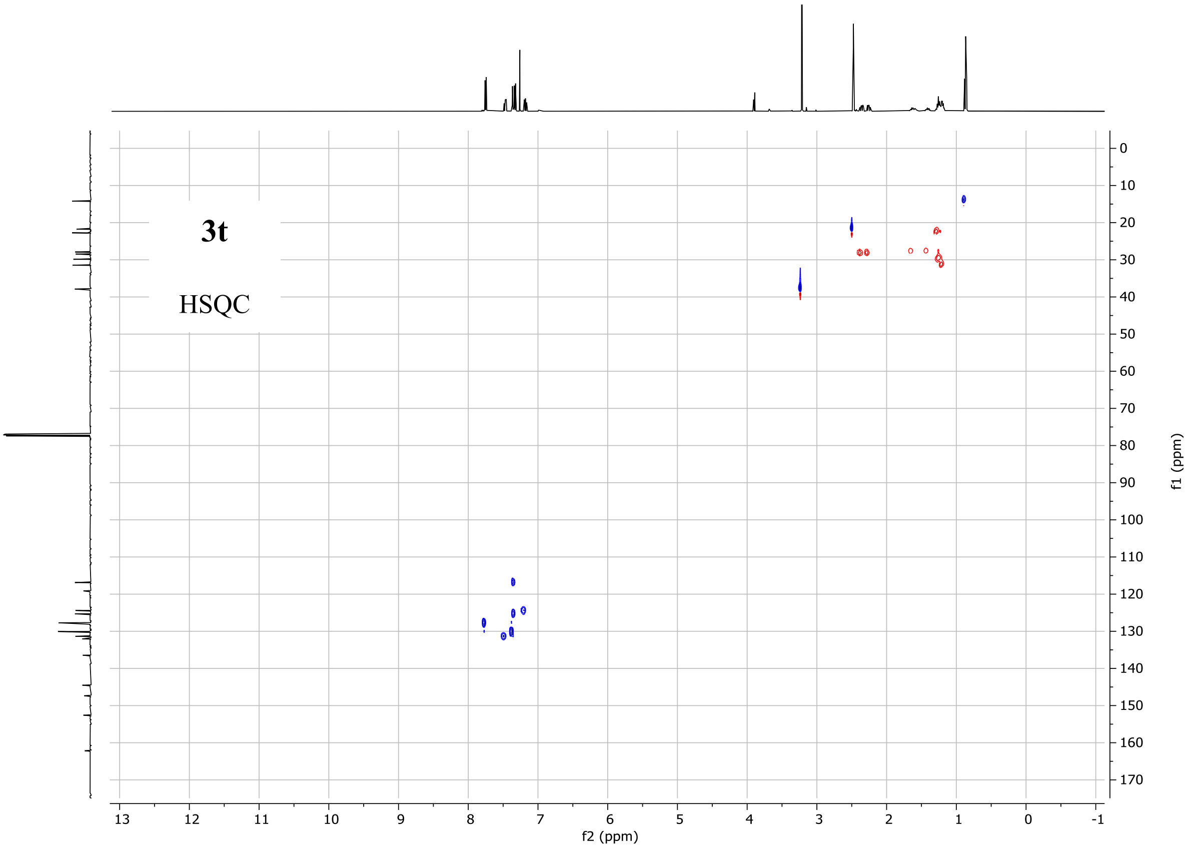
3t
HSQC
f2 (ppm)
f1 (ppm)

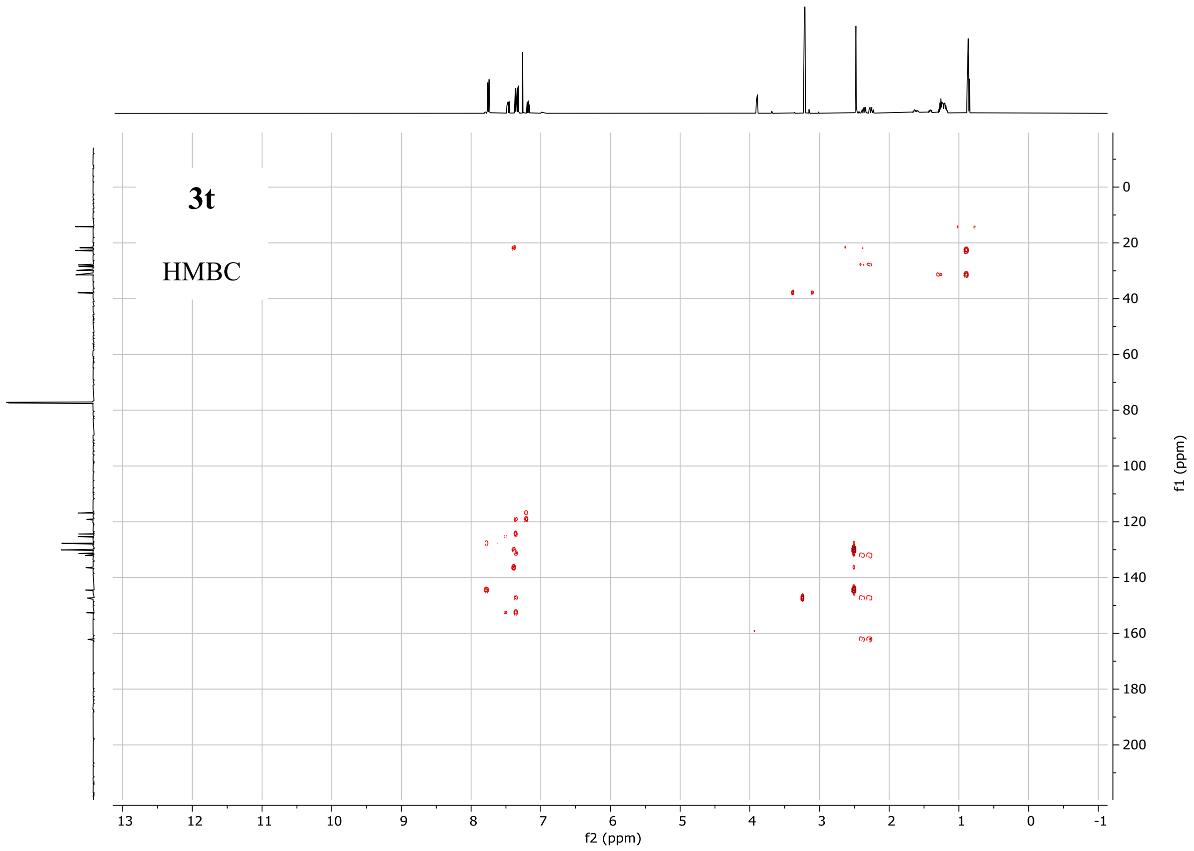
3t
HMBC
f2 (ppm)
f1 (ppm)

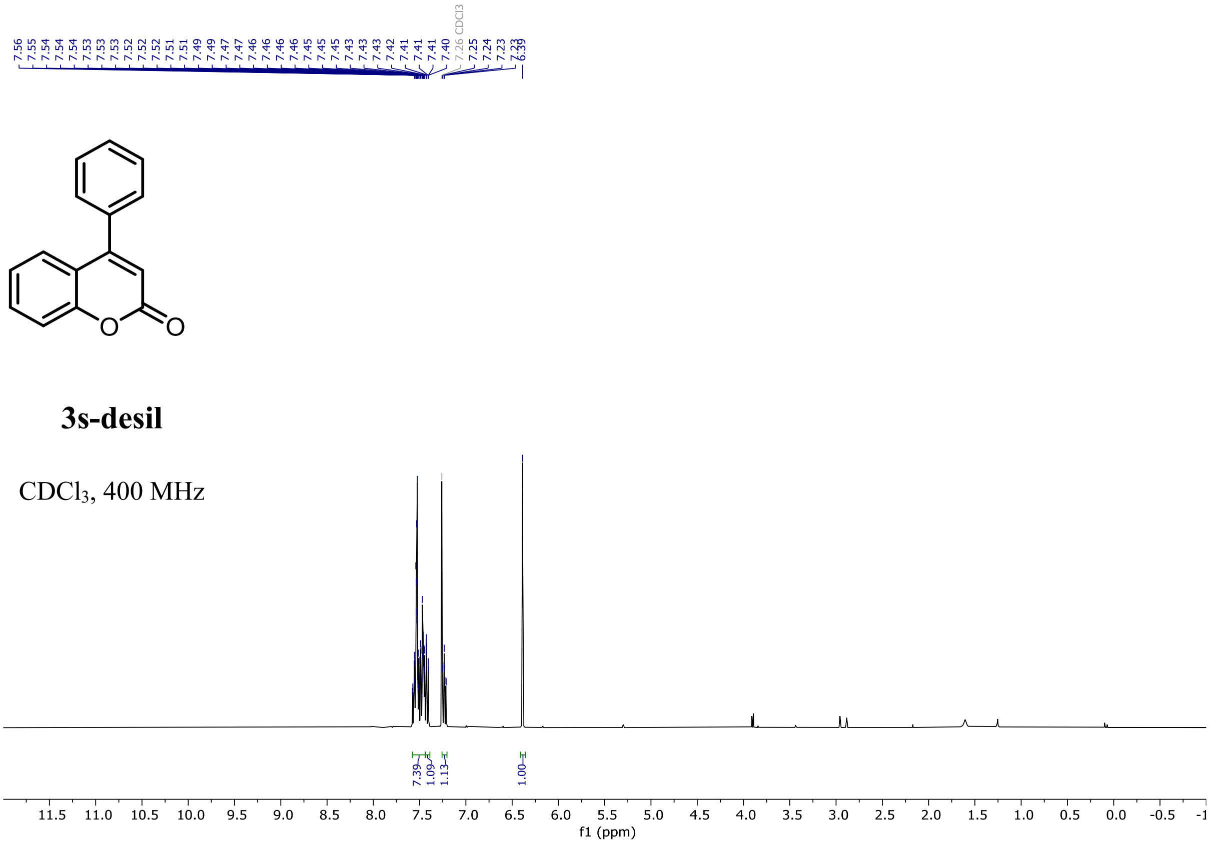
7.56
7.55
7.54
7.54
7.54
7.53
7.53
7.53
7.52
7.52
7.52
7.51
7.51
7.49
7.49
7.47
7.47
7.46
7.46
7.46
7.45
7.45
7.45
7.45
7.43
7.43
7.43
7.43
7.42
7.41
7.41
7.41
7.40
7.26 CDCl3
7.25
7.24
7.23
7.23
6.39
O
O
3s-desil
CDCl3, 400 MHz
7.39
1.09
1.13
1.00
11.5 11.0 10.5 10.0 9.5 9.0 8.5 8.0 7.5 7.0 6.5 6.0 5.5 5.0 4.5 4.0 3.5 3.0 2.5 2.0 1.5 1.0 0.5 0.0 -0.5
f1 (ppm)

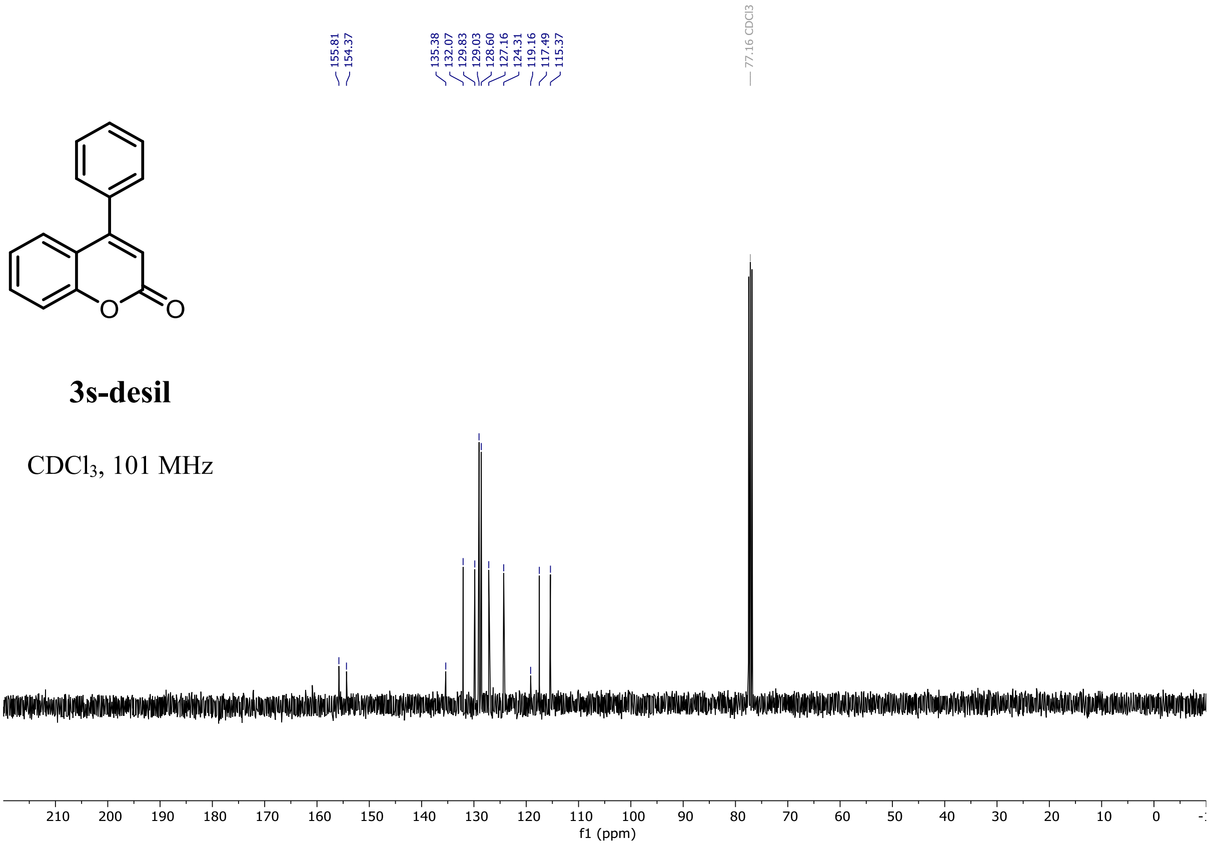
155.81
154.37
135.38
132.07
129.83
129.03
128.60
127.16
124.31
119.16
117.49
115.37
77.16 CDCl3
O
O
3s-desil
CDCl3, 101 MHz
210 200 190 180 170 160 150 140 130 120 110 100 90 80 70 60 50 40 30 20 10 0
f1 (ppm)

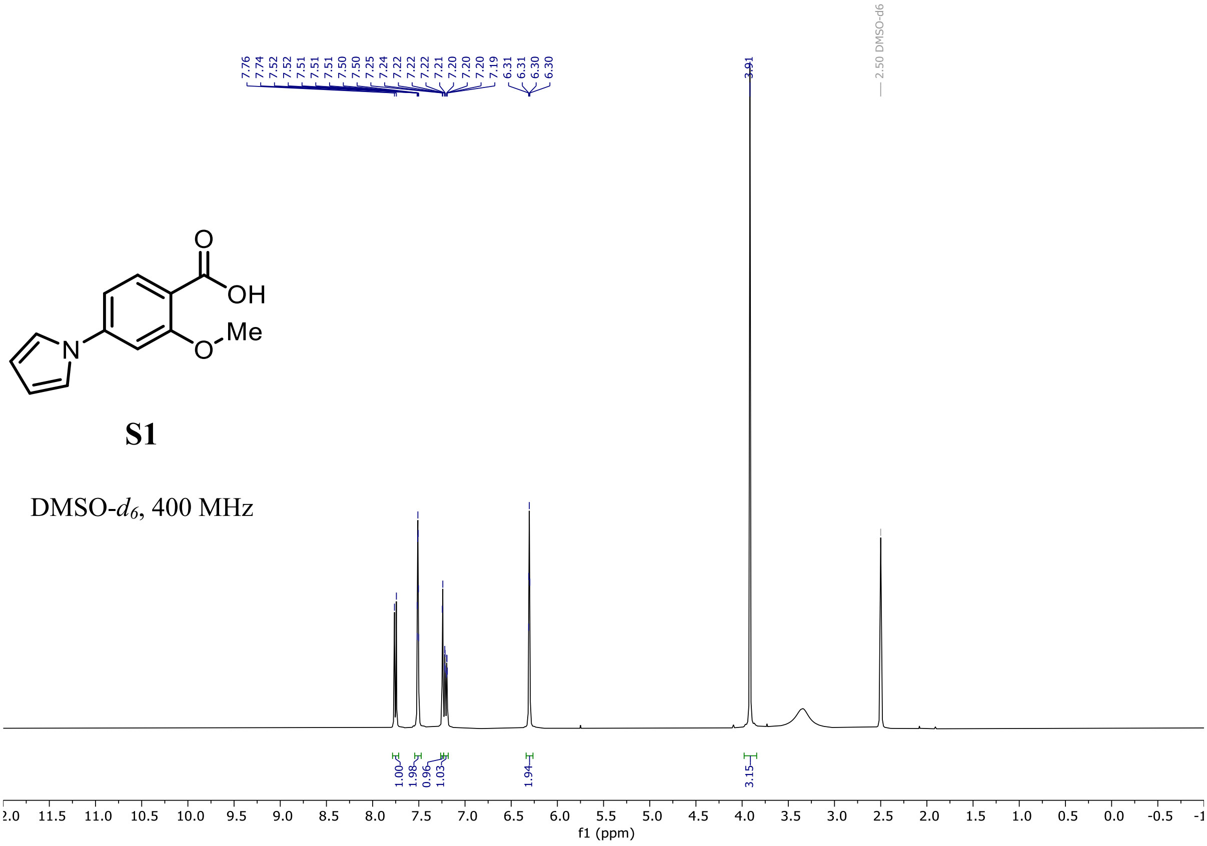
7.76 7.74 7.52 7.52 7.51 7.51 7.51 7.50 7.50 7.25 7.24 7.22 7.22 7.22 7.21 7.20 7.20 7.20 7.19 6.31 6.31 6.30 6.30
3.91
2.50 DMSO-d6
OH
O
N
Me
S1
DMSO-d_6, 400 MHz
1.00 1.98 0.96 1.03 1.94 3.15
f1 (ppm)

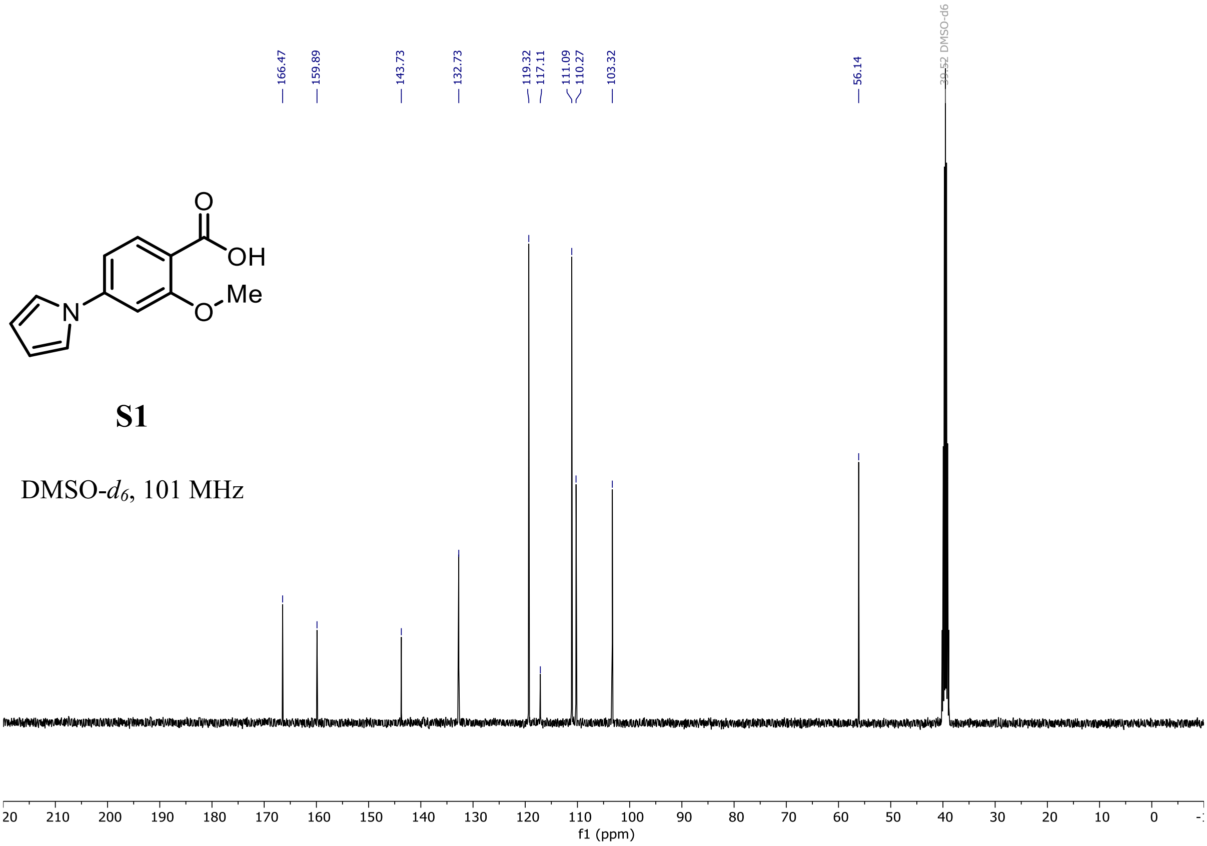
166.47
159.89
143.73
132.73
119.32 117.11
111.09 110.27
103.32
56.14
39.52 DMSO-d6
OH
O
N
Me
S1
DMSO-d_6, 101 MHz
f1 (ppm)

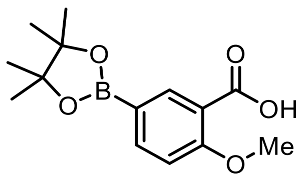

S2

CDCl$_3$, 500 MHz

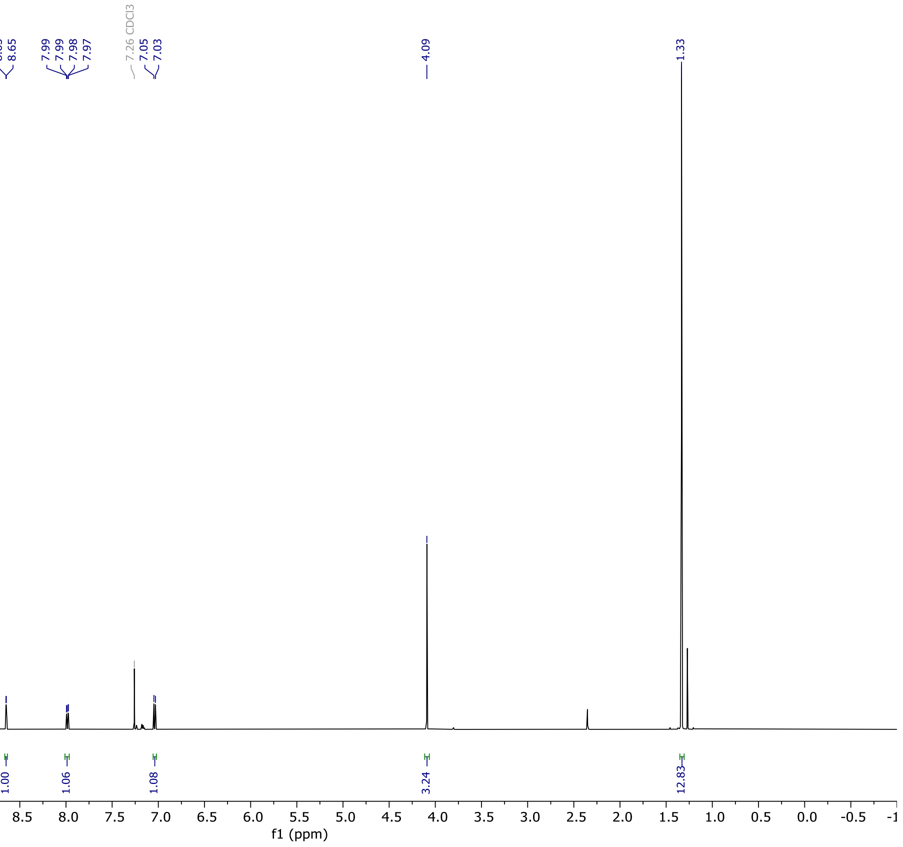

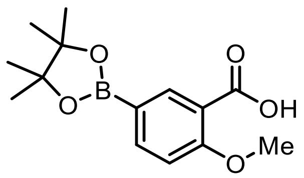

S2

CDCl$_3$, 125 MHz

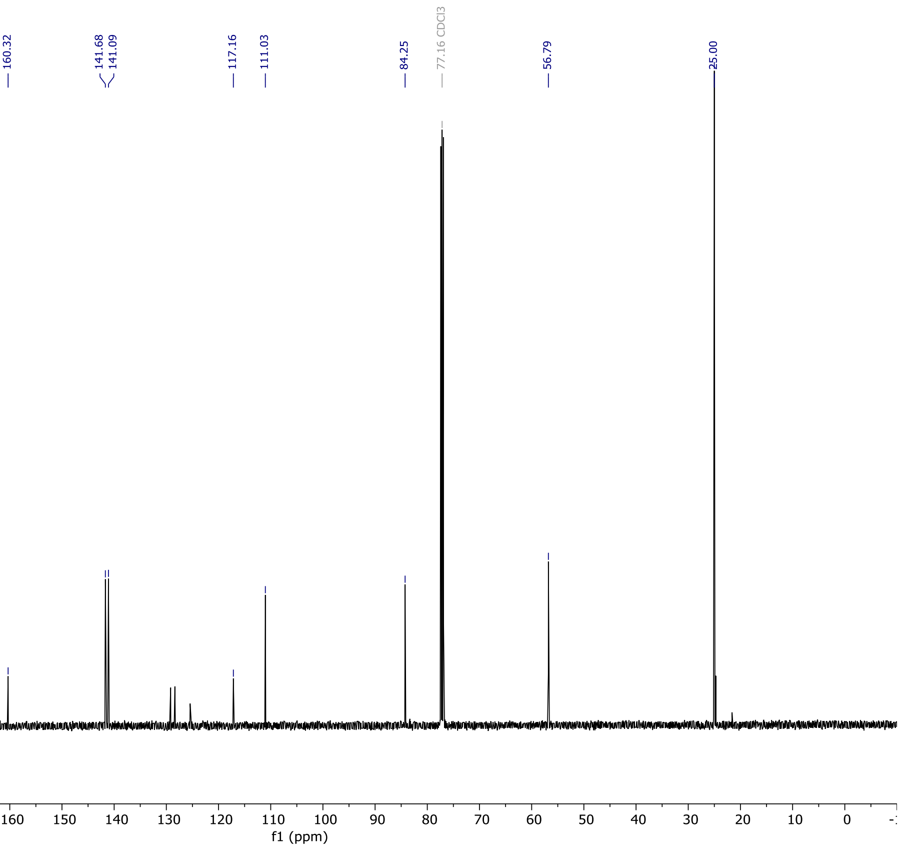

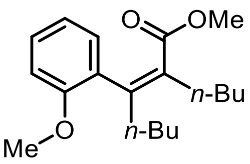

6a

CDCl$_3$, 500 MHz

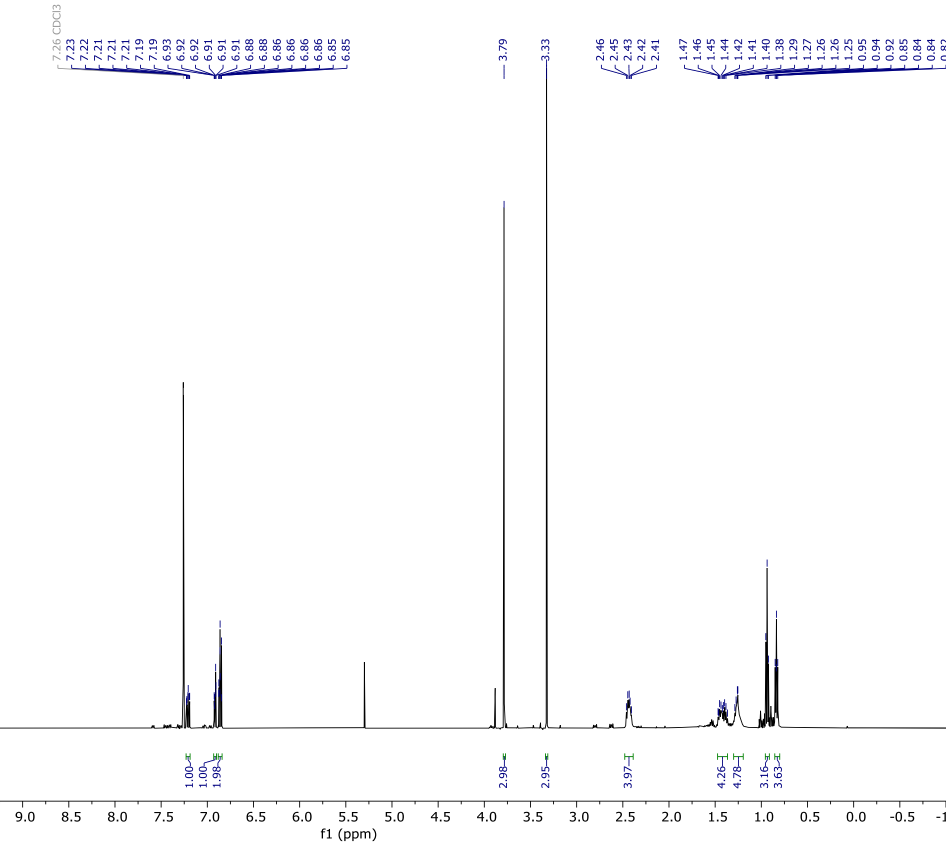

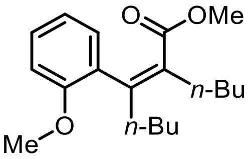

6a

CDCl$_3$, 125 MHz

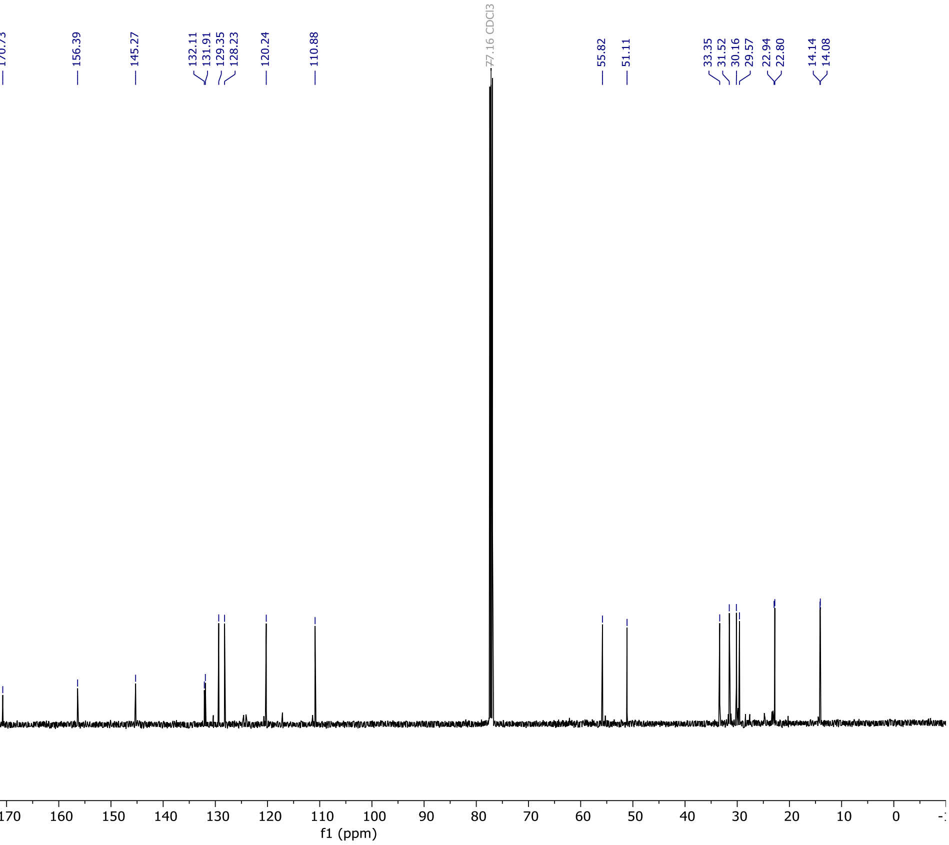

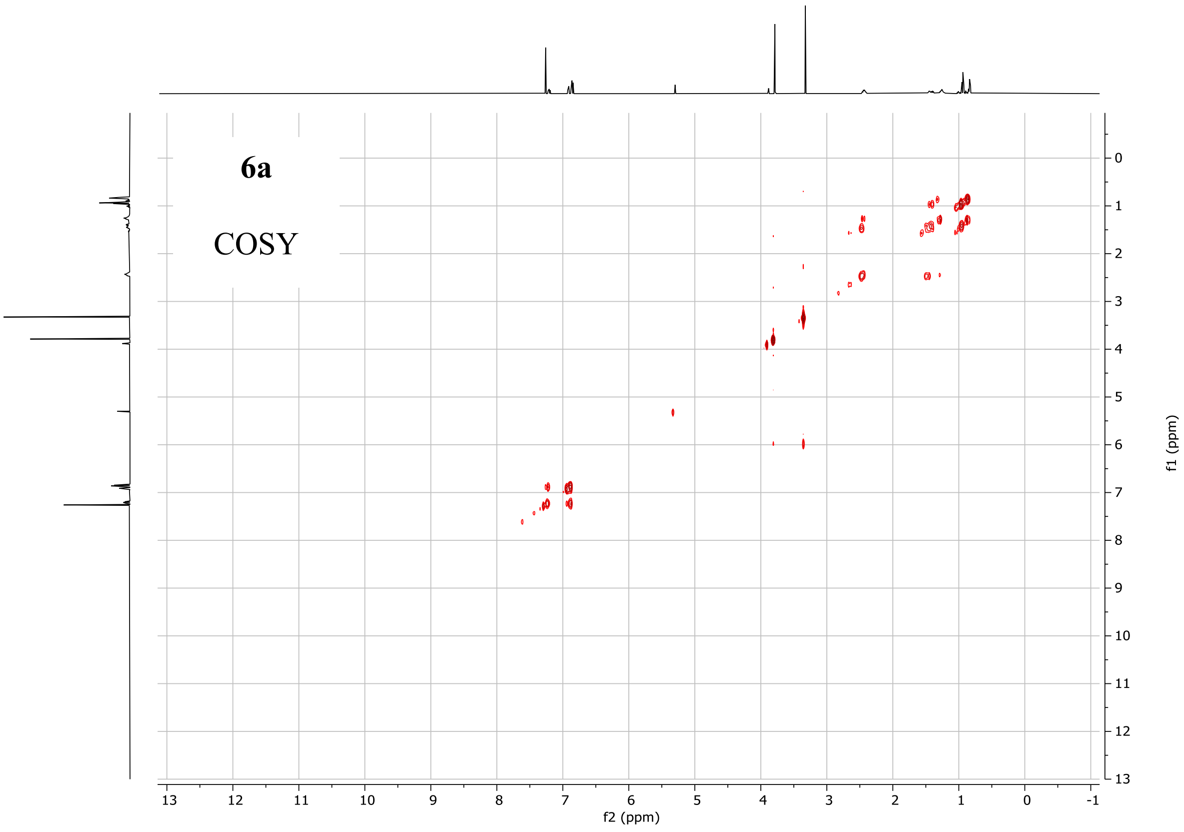
6a
COSY
f2 (ppm)
f1 (ppm)

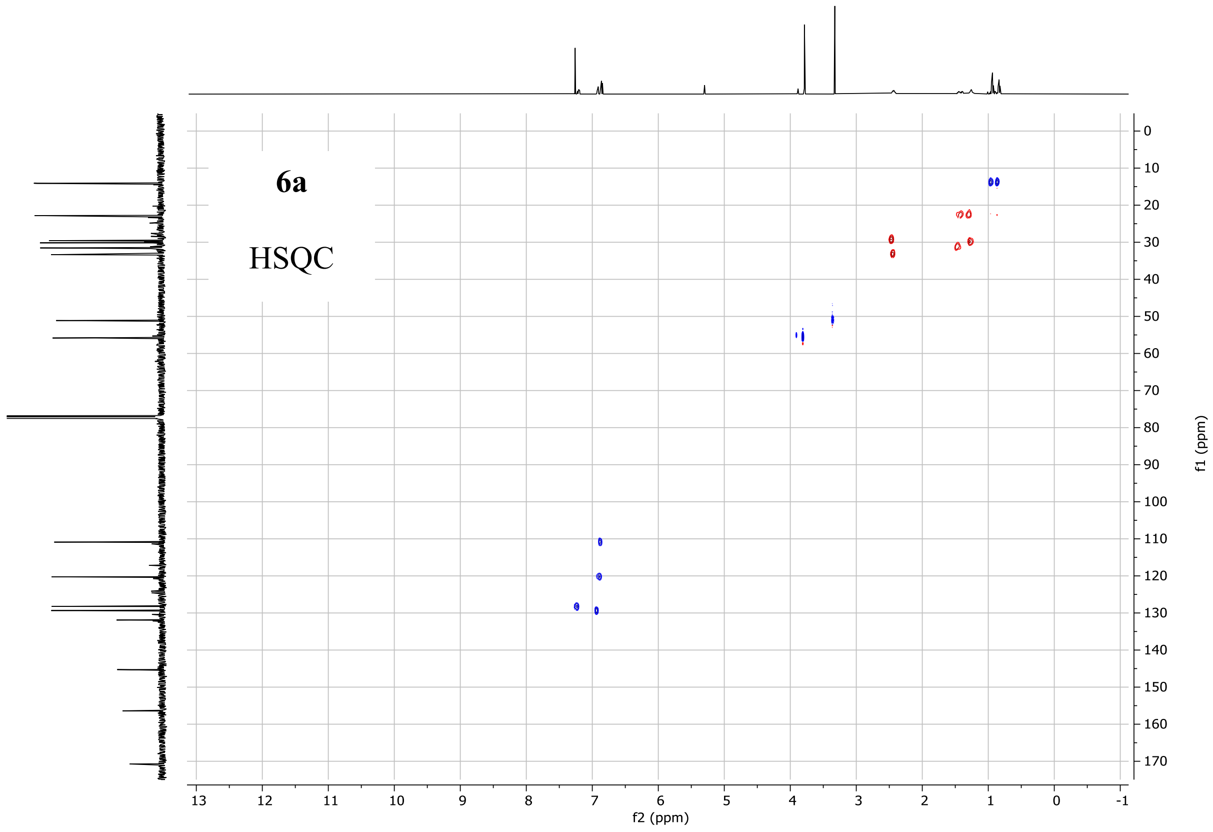
6a
HSQC
f2 (ppm)
f1 (ppm)

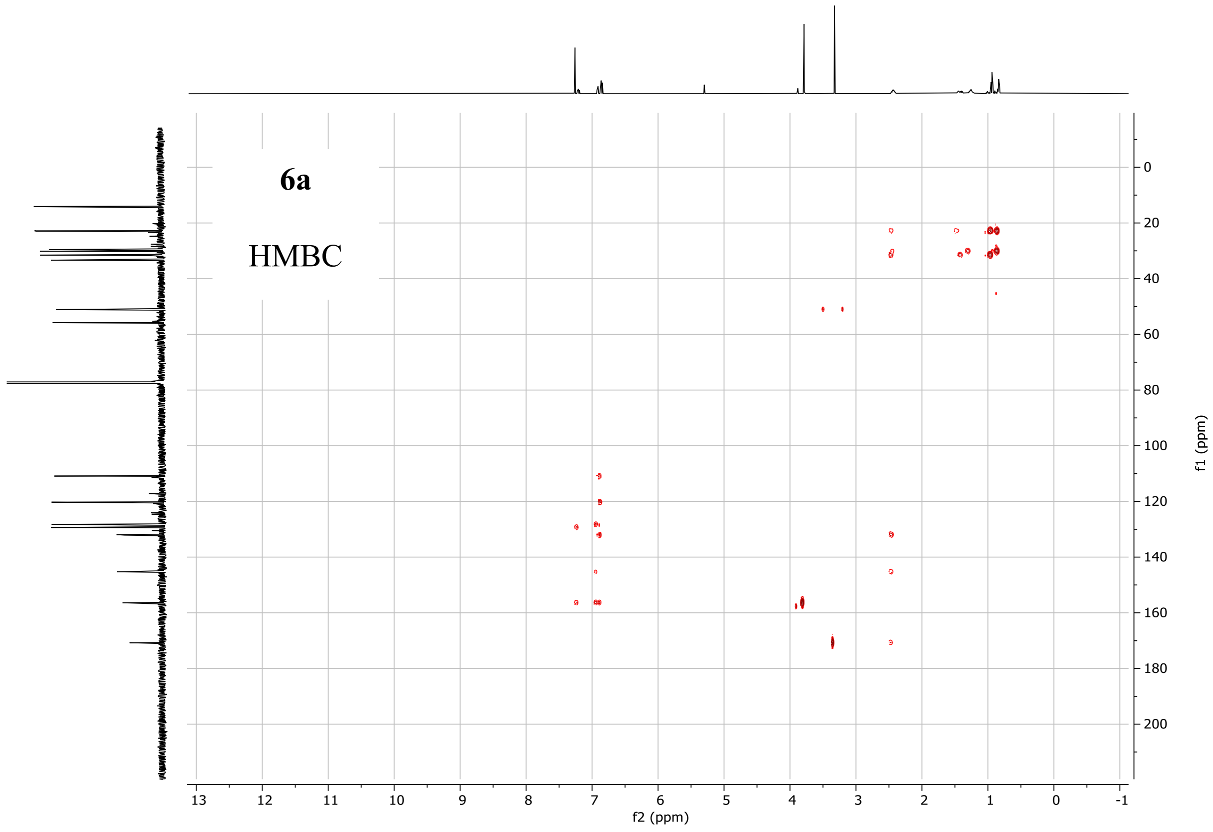
6a
HMBC
f1 (ppm)
f2 (ppm)

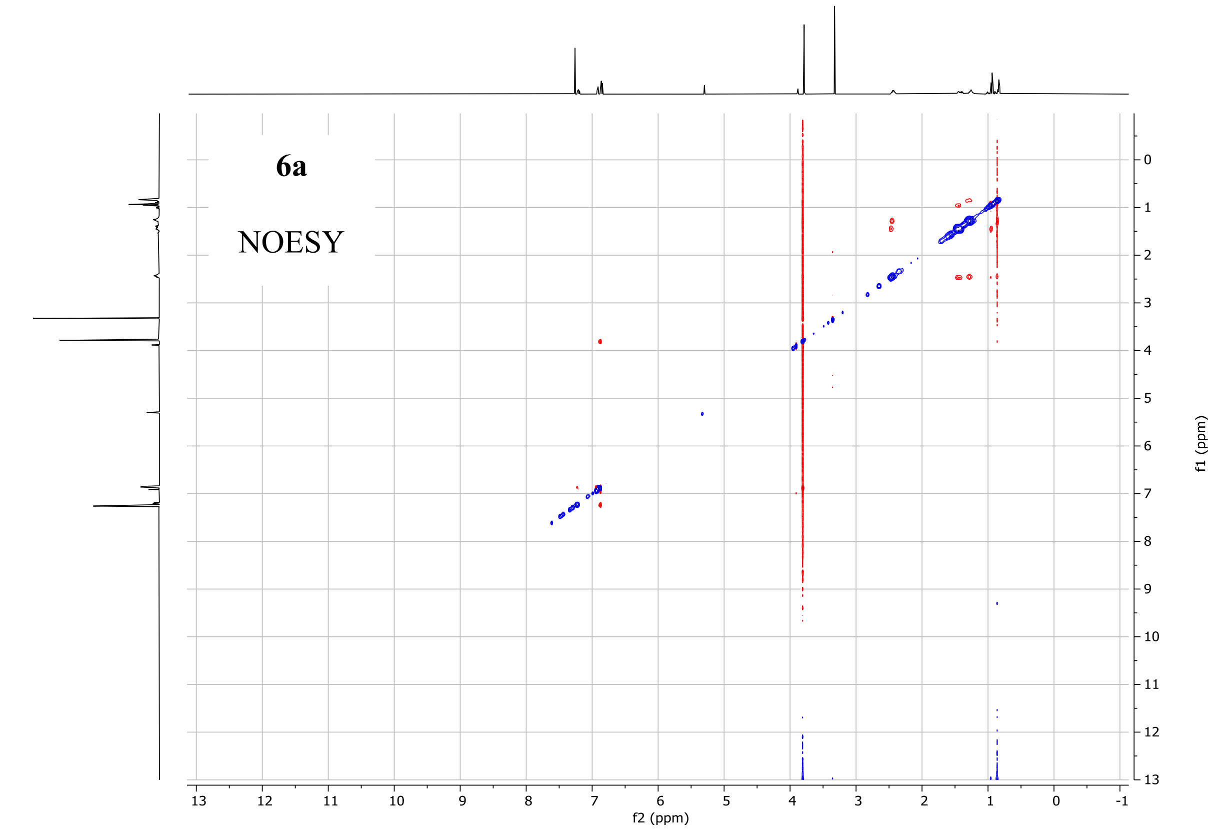
6a
NOESY
f1 (ppm)
f2 (ppm)

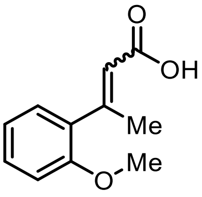

7u

CDCl$_3$, 500 MHz

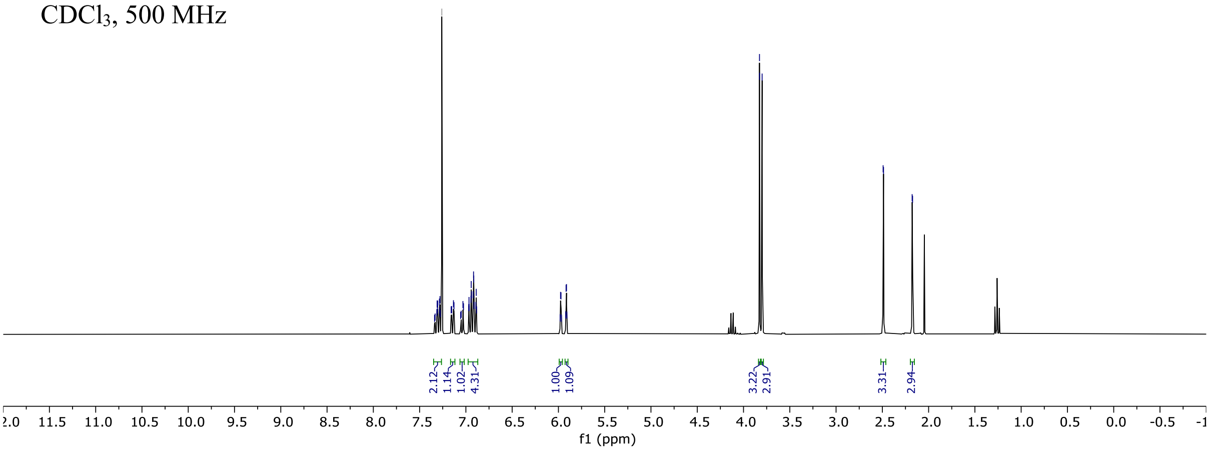

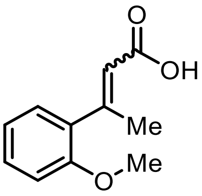

7u

CDCl$_3$, 125 MHz

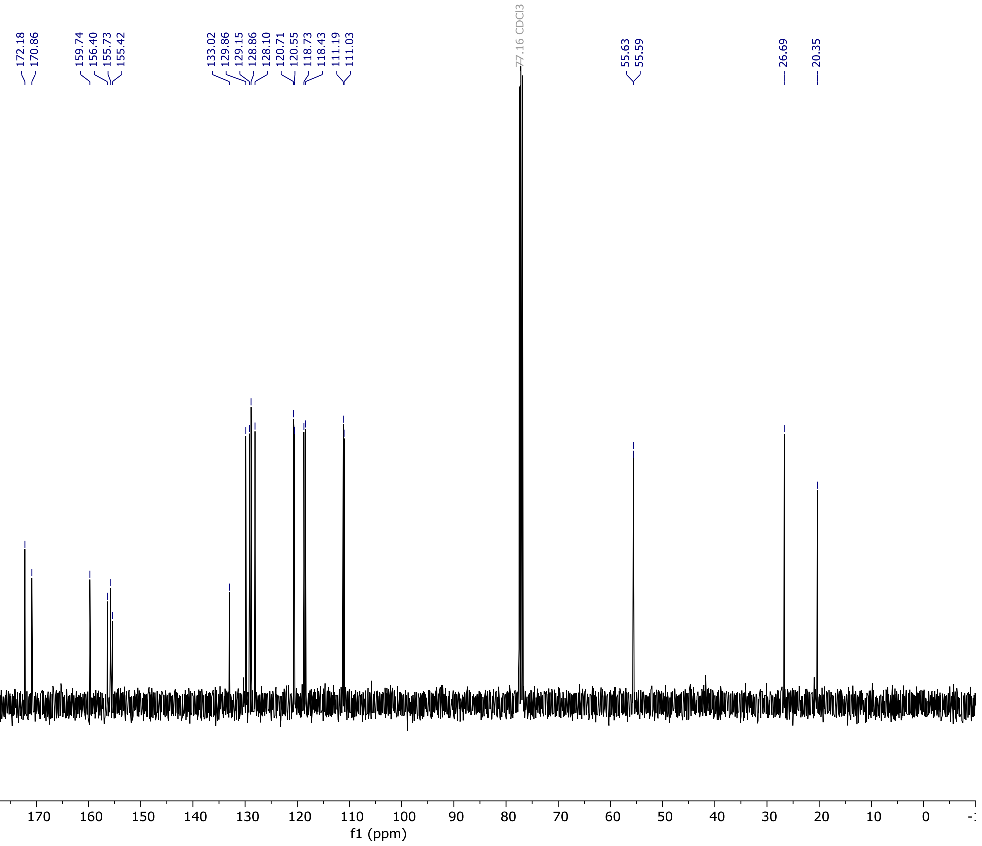

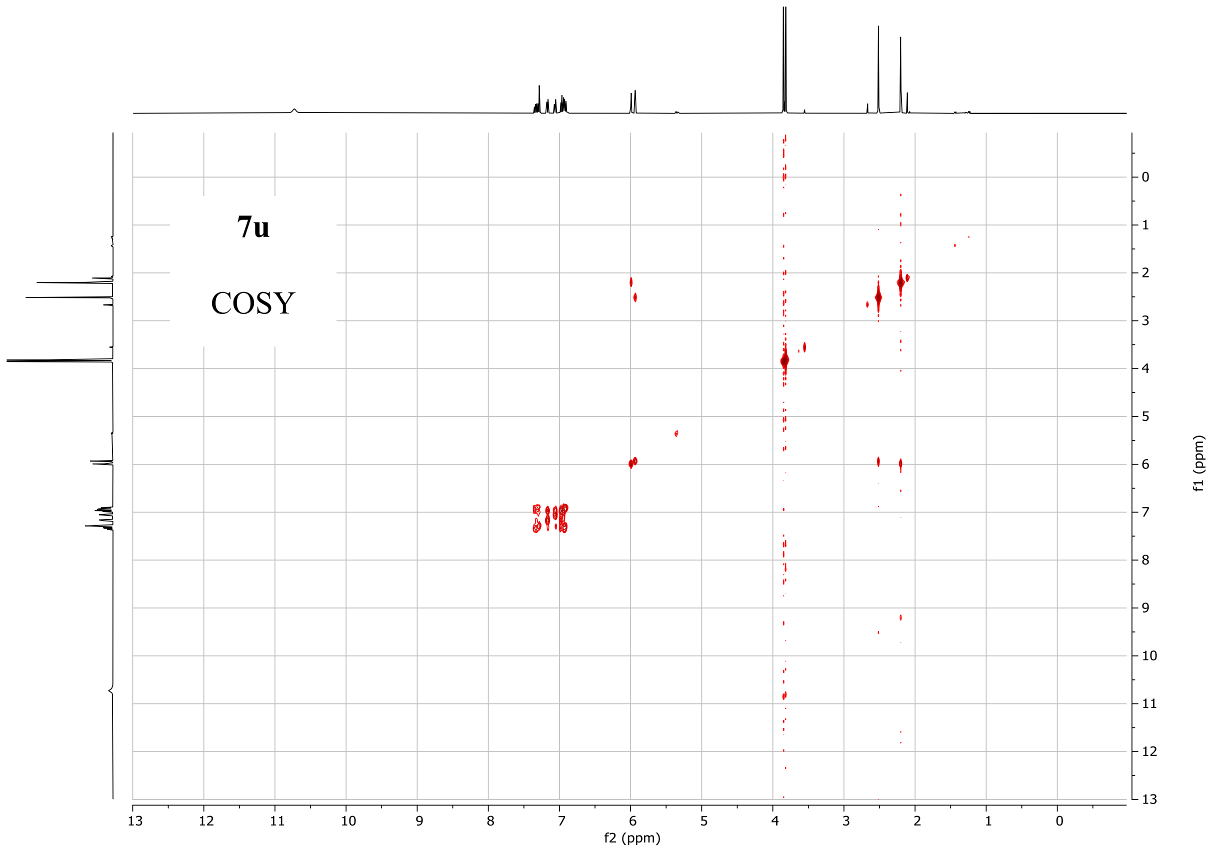
7u
COSY
f2 (ppm)
f1 (ppm)

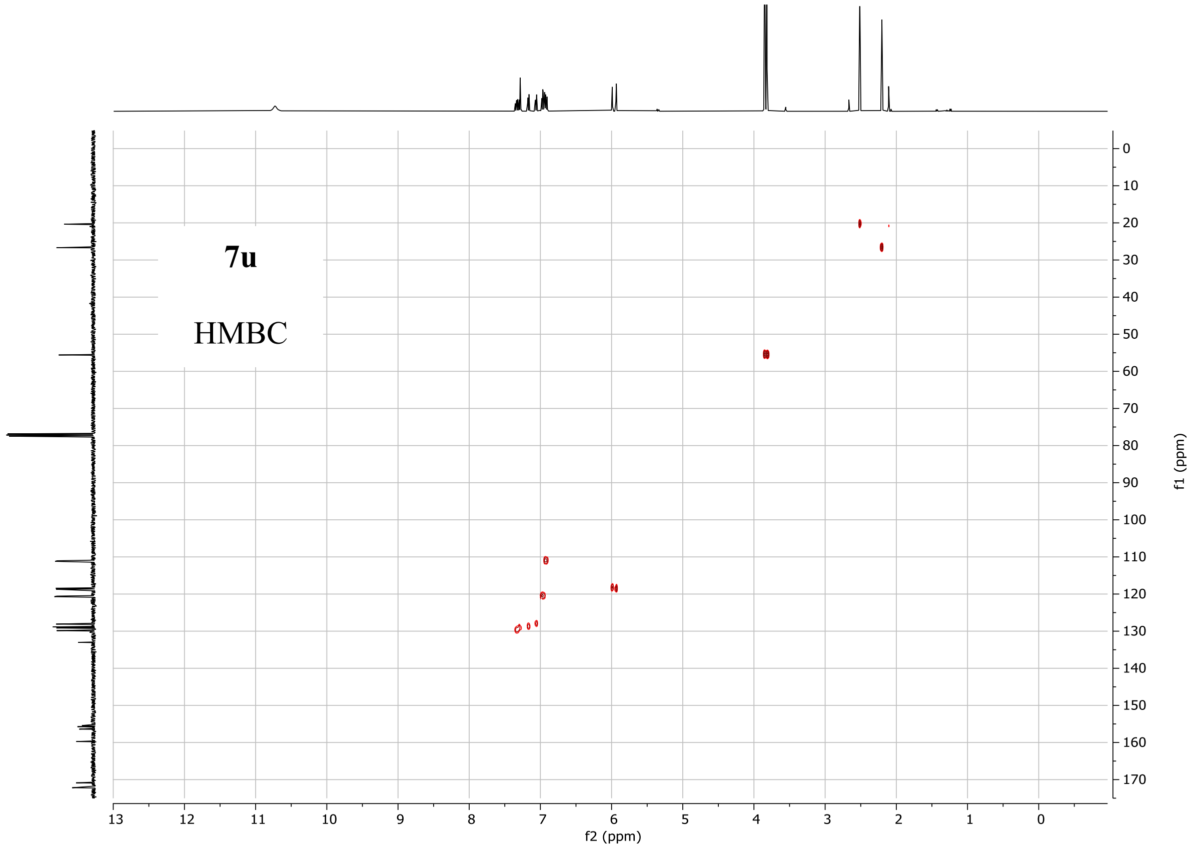
7u
HMBC
f2 (ppm)
f1 (ppm)

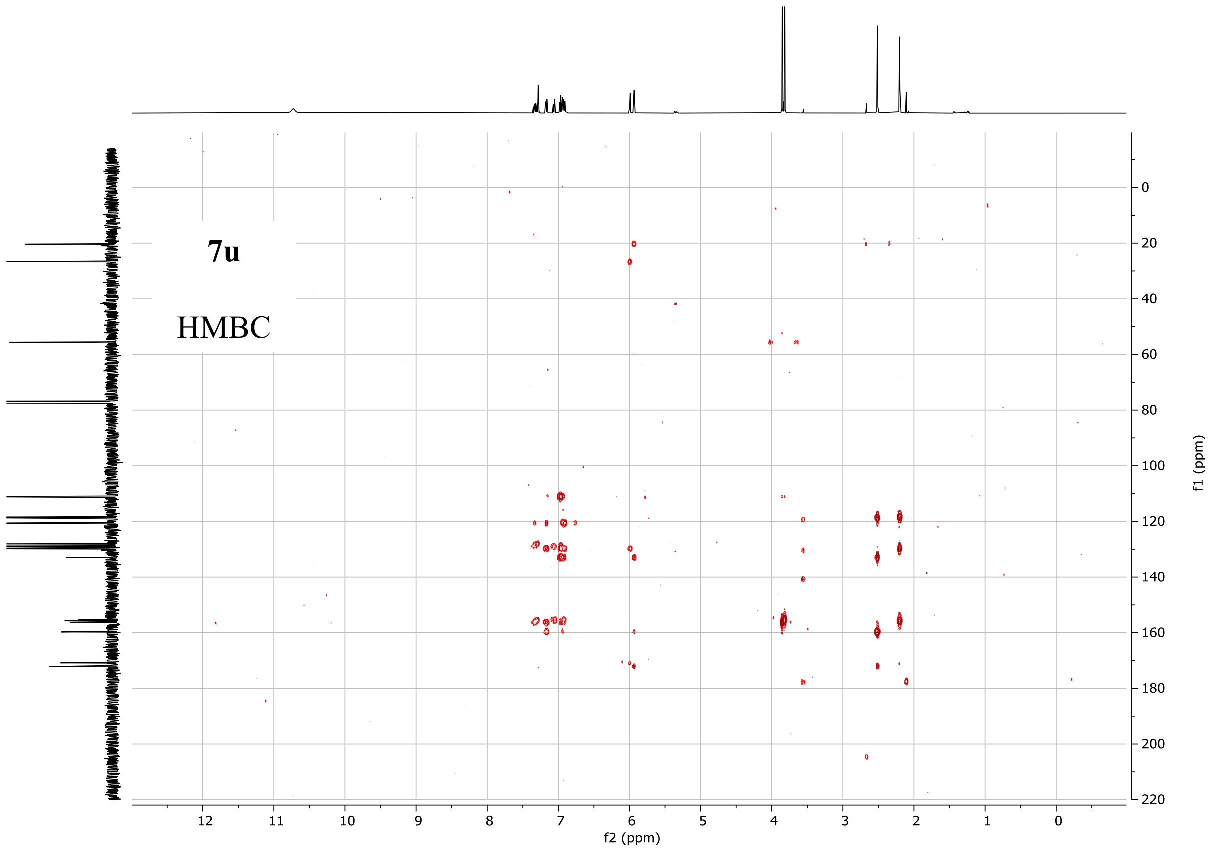
7u
HMBC
f2 (ppm)
f1 (ppm)
